# FluoSim: simulator of single molecule dynamics for fluorescence live-cell and super-resolution imaging of membrane proteins

**DOI:** 10.1038/s41598-020-75814-y

**Published:** 2020-11-17

**Authors:** Matthieu Lagardère, Ingrid Chamma, Emmanuel Bouilhol, Macha Nikolski, Olivier Thoumine

**Affiliations:** 1 CNRS, Interdisciplinary Institute for Neuroscience, IINS, UMR 5297, Univ. Bordeaux, 33000 Bordeaux, France; 2 CNRS, IBGC, UMR 5095, Univ. Bordeaux, 33000 Bordeaux, France

**Keywords:** Biophysics, Computational biology and bioinformatics, Neuroscience

## Abstract

Fluorescence live-cell and super-resolution microscopy methods have considerably advanced our understanding of the dynamics and mesoscale organization of macro-molecular complexes that drive cellular functions. However, different imaging techniques can provide quite disparate information about protein motion and organization, owing to their respective experimental ranges and limitations. To address these issues, we present here a robust computer program, called FluoSim, which is an interactive simulator of membrane protein dynamics for live-cell imaging methods including SPT, FRAP, PAF, and FCS, and super-resolution imaging techniques such as PALM, dSTORM, and uPAINT. FluoSim integrates diffusion coefficients, binding rates, and fluorophore photo-physics to calculate in real time the localization and intensity of thousands of independent molecules in 2D cellular geometries, providing simulated data directly comparable to actual experiments. FluoSim was thoroughly validated against experimental data obtained on the canonical neurexin-neuroligin adhesion complex at cell–cell contacts. This unified software allows one to model and predict membrane protein dynamics and localization at the ensemble and single molecule level, so as to reconcile imaging paradigms and quantitatively characterize protein behavior in complex cellular environments.

## Introduction

Critical cellular functions such as membrane adhesion, receptor-mediated signaling, or synaptic transmission, involve the diffusional trapping of specific molecules in sub-cellular compartments^1,2^. To quantitatively describe such molecular dynamics in living cells, several fluorescence imaging techniques are currently available^3,4^: (1) single particle tracking (SPT) which resolves the motion of individual proteins at camera frame rate; (2) photo-activation and photo-bleaching methods, namely PhotoActivation of Fluorescence (PAF) and Fluorescence Recovery After Photobleaching (FRAP) which infer protein turnover at the population level; and (3) Fluorescence Correlation Spectroscopy (FCS), which analyzes molecular dynamics by correlating intensity fluctuations. More recent approaches based on single molecule localization such as PhotoActivated Localization Microscopy (PALM)^5,6^, direct Stochastic Optical Reconstruction Microscopy (dSTORM)^7^, and Point Accumulation In Nanoscopic Topography (PAINT)^8^, yield images of protein distribution at improved resolution (below 50 nm), giving unprecedented information about the nanoscale organization of biological structures^9^.

Despite such progress in imaging power, many experimental parameters remain difficult to estimate or control, including protein expression levels, probe labeling density, potential fixation artifacts, spatial and temporal sampling of the recordings, and protein motion below the system resolution, such that results from different experimental paradigms are often difficult to reconcile^10^. Thus, there is a pressing need for computer simulators that could unify those different imaging modes in a unique framework, estimate their respective biases, and serve as a predictive tool for experimenters, with the aim to quantitatively decipher protein organization and dynamics in living cells. Several particle-based packages relying on Monte Carlo simulations already exist to predict random motion and multi-state reactions of biological molecules, but either they do not integrate fluorescence properties or are limited to a specific type of imaging mode, and are usually not performing real-time visualization^11–18^.

In this context, we provide here fast, robust, and user-friendly software (*FluoSim*) that allows real time simulation of membrane protein dynamics in live-cell imaging (SPT, FRAP, PAF, and FCS) and super-resolution (PALM, dSTORM, uPAINT) modalities. We also show that FluoSim can be further used to produce large virtual data sets for training deep neural networks for image reconstruction^19^. This software should thus be of great interest to a wide community specialized in imaging methods applied to cell biology and neuroscience, with the common aim to better understand membrane dynamics and organization in cells.

## Results

### General principle of FluoSim

**Figure 1:**
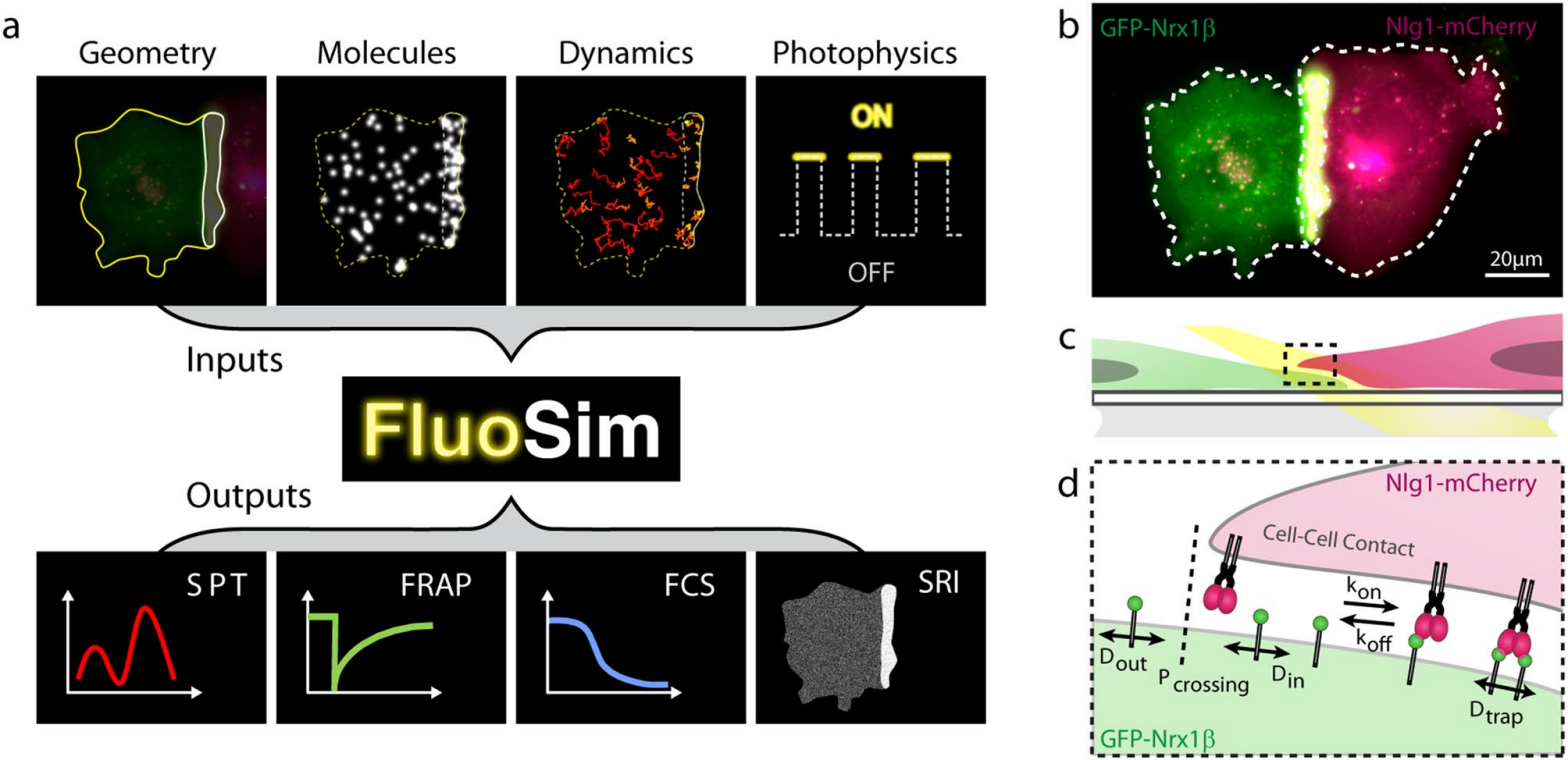
Schematics of the simulator and experimental system. (**a**) General principle of FluoSim. (**b**) Contact between two COS cells, one expressing GFP-Nrx1β (green) and the other expressing Nlg1-mCherry (magenta), resulting in molecule accumulation through adhesive interactions (yellow zone). (**c**) Diagram showing a zoomed section of the cell–cell interface at the coverslip; the yellow beam represents oblique laser illumination. (**d**) Cartoon of Nrx1β diffusional trapping by Nlg1 in a cell–cell contact.

The FluoSim interface looks like performing a real experiment: the user imports a 2D cellular geometry from a microscopy image, and populates it with a realistic number of molecules (a parameter which depends on protein expression level, cell surface area, and labeling density) (Fig. 1a, Supplemental software and accompanying user manual). Kinetic parameters characterizing the diffusion and trapping of molecules in cellular regions of interest (ROI), are entered as inputs (Table 1). At each time increment (typically ~ 1–100 ms, adjusted to sensor acquisition rates), the algorithm updates the instantaneous positions of all molecules based on random number generation. Photo-switching rates of organic fluorophores or fluorescent proteins attached to the proteins of interest, further determine the fluorescence intensity associated to each molecule over time. The algorithm is optimized to visualize in real time the cellular system and provide post-processing information including molecule trajectories, image stacks, and output graphs (i.e. histograms of diffusion coefficients, FRAP, PAF, and FCS curves). Examples of simulated data sets for a realistic range of parameter values are given in Fig. S1. Moreover, since the positions of molecules are known with near-infinite accuracy, the program can generate super-resolved images comparable to those obtained with PALM or dSTORM, after introducing additional parameters describing protein labeling density, fluorophore duty cycle, and localization precision of the system. Finally, various levels of noise were introduced in the simulator, including a Poisson shot noise characterizing intensity fluctuations of individual fluorescent molecules, and a Gaussian readout noise applied around the camera offset^20,21^. This approach led to the simulation of very realistic images that closely matched those obtained by Total Internal Reflection Fluorescence (TIRF) imaging of single GFP-tagged molecules immobilized on a glass substrate (Fig. S2). A complete view of the parameters used in each experimental paradigm is available from the individual examples provided in the software menu.

### Experimental system to validate FluoSim

To thoroughly validate FluoSim, we performed SPT, dSTORM, FRAP, and FCS experiments essentially on the canonical neurexin-neuroligin complex that mediates trans-synaptic adhesion in neurons^22^. We used COS-7 cells as a model expression system because they form large and flat lamellipodia that can be approximated as 2D environments for membrane diffusion. Cells were separately electroporated with recombinant GFP-tagged neurexin-1β (GFP-Nrx1β) and mCherry-tagged neuroligin-1 (Nlg1-mCherry), then cultured together for 24 h (Fig. 1b–d). Both molecules reached the cell membrane and accumulated at cell–cell contacts (GFP-Nrx1β enrichment = 3.9 ± 0.5, n = 20 cells), revealing adhesive interactions.

### Simulations of SPT experiments

**Figure 2:**
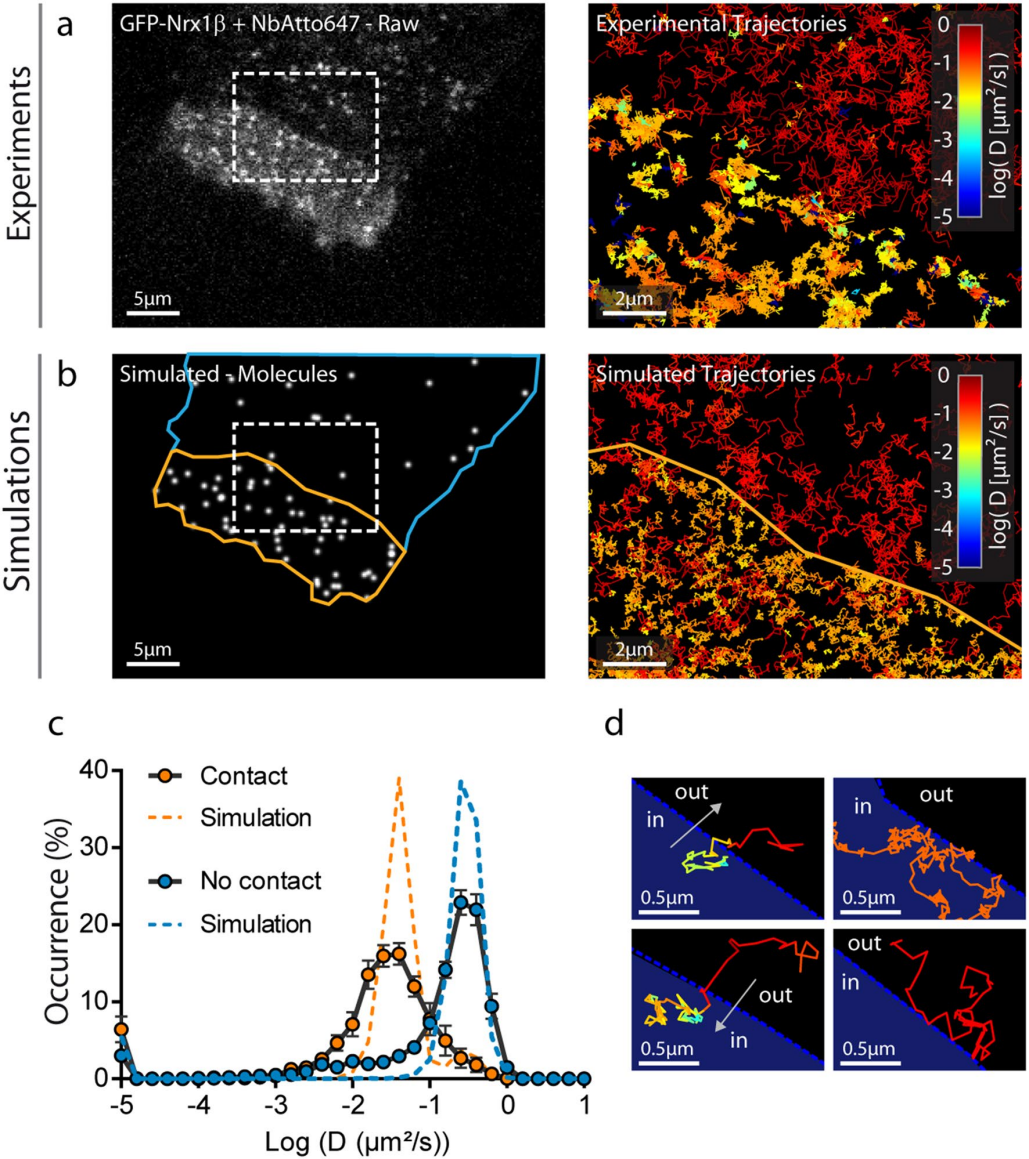
Fitting SPT experiments. (**a**) Raw image of a COS-7 cell expressing GFP-Nrx1β sparsely labeled with Atto647N-conjugated GFP nanobody (white signal). (**b**) Image of simulated molecules in the same geometry. Rectangles highlight the interface between the cellular region with freely diffusing GFP-Nrx1β molecules (blue outline), and the contact region with a cell expressing Nlg1-mcherry (yellow outline). On the right, a zoom on this ROI shows single molecule trajectories for both experiments and simulations. The diffusion coefficient expressed in log scale is color coded. (**c**) Distributions of GFP-Nrx1β diffusion coefficients for experiments (circles, average ± sem of 3 cells, 4145 trajectories in contact and 4867 outside contacts) and simulations (dashed lines, 5 repetitions, 8757 trajectories in contact and 2645 trajectories outside contacts) on a semi-log plot. The Spearman correlation coefficient comparing experiment and simulation was r = 0.85 (*P* < 0.001, n = 36 bins) for contact regions, and r = 0.81 (*P* < 0.001, n = 36 bins) for outside regions. (**d**) Representative examples of single molecule trajectories at the interface: (left) a molecule escapes the contact and diffuses out freely, or enters the contact and gets trapped; (right) a molecule stays stuck in the contact with low diffusion, or bounces on the contact without entering it.

First, we experimentally tracked single GFP-Nrx1β molecules labeled with Atto647N-conjugated anti-GFP nanobody at 50 Hz using uPAINT^8,23,24^. Nrx1β exhibited fast free diffusion outside the adhesive contact (D_out_ = 0.3 µm^2^/s), and was slowed down by ~ tenfold in the contact, reflecting the formation of Nrx1β-Nlg1 bonds between apposed membranes (D_trap_ = 0.04 µm^2^/s) (Fig. 2a,c). Nrx1β molecules often bounced at the contact border, revealing steric hindrance to penetrate the narrow cell–cell junction^25^, an effect which was described by a crossing probability (P_crossing_ < 1) (Fig. 2d). Next, we simulated Nrx1β diffusion using FluoSim (Fig. 2b). We used the diffusion coefficients obtained experimentally and defined a sparse number of molecules (250) corresponding to the number of experimental detections per frame, together with two kinetic rates describing the Nrx1β-Nlg1 interaction taken from the literature (k_on_ = 0.15 s^−1^ and k_off_ = 0.015 s^−1^)^26,27^. We also chose realistic photophysics parameters (k_on_^Fluo^ = 1 s^−1^ and k_off_^Fluo^ = 3 s^−1^) to match experimental trajectory number and duration, and single molecule rendering parameters for Atto647N (σ = 0.22 µm, FWHM = 0.53 µm). Using these parameters, FluoSim generated diffusion coefficient distribution curves inside and outside the contact that aligned well on experimental data (Fig. 2c). Experimental distributions were somewhat more spread than theoretical ones, most likely because of local membrane heterogeneities which are not accounted for in the model.

### Simulations of dSTORM experiments

**Figure 3:**
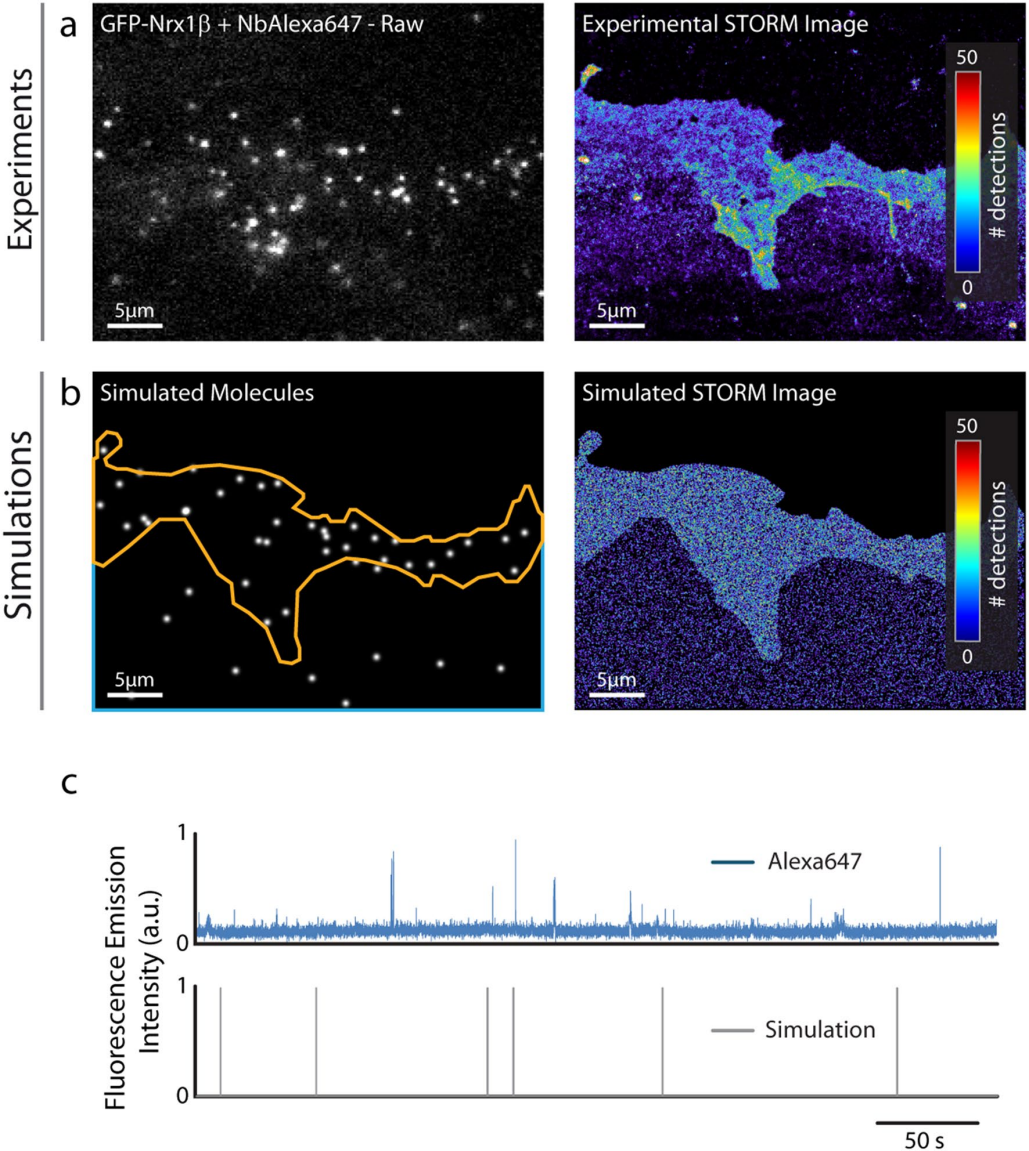
Fitting SRI experiments. (**a**) Representative single frame image of a STORM sequence performed on GFP-Nrx1β labeled with Alexa647-conjugated GFP nanobody, and corresponding super-resolved image generated from 3.57 × 10^6^ single molecule localizations (pixel size 32 nm, total acquisition time 1600 s). The number of molecules detected per pixel is color coded. (**b**) Simulated image showing single molecule fluorescence emission in the same cell geometry, and corresponding super-resolved map with a localization precision of 58 nm (FWHM). The total number of detections is 3.61 × 10^6^. (**c**) Fluorescence emission over time from an isolated Alexa647-conjugated GFP nanobody bound to the glass coverslip in a STORM sequence, and simulated emission of fluorescence of an immobile molecule obtained with switch-on and -off rates of 0.006 s^−1^ and 9.3 s^−1^, respectively.

To simulate dSTORM experiments that were experimentally performed on GFP-Nrx1β using saturating labeling with Alexa647-conjugated GFP nanobody (Fig. 3a), we introduced a relatively large number of molecules in the imported geometry (70,000, corresponding to the sum of experimental detections per frame integrated over the cell surface area), and set all diffusion coefficients to zero to mimic cell fixation. To simulate stochastic fluorescence emission of Alexa647^28^, we calculated the switch-on (k_on_^Fluo^ = 0.006 s^−1^) and switch-off (k_off_^Fluo^ = 9.3 s^−1^) rates of Alexa647 fluorophores from isolated Alexa647-conjugated nanobodies in dSTORM imaging conditions (Fig. 3c). We then simulated the accumulation of single molecule localizations for 80,000 frames, including a realistic localization precision (σ = 25 nm, FWHM = 58 nm), to mimic the experimental super-resolved maps of Nrx1β distribution (Fig. 3b). Simulated images had intensity values very similar to experimental ones, but as expected from an idealistic model, could not capture specific membrane features (e.g. ruffles) seen experimentally (also see Fig. S4 for a more homogeneous cell–cell contact).

### Training of deep neural networks for image reconstruction

Deep learning is becoming increasingly popular for image reconstruction in fluorescence microscopy^19,29,30^. Convolutional Neural Networks (CNNs) are especially relevant for image treatment and have to be trained using large exemplary data sets obtained either experimentally, or from simulations. In this context, we tested the ability of FluoSim to train deep CNNs based on simulated data. We generated large image sets of randomly distributed single molecules represented as Gaussian intensity profiles plus Poisson noise, together with their localization maps as ground truth, and trained the previously described CNN called Deep-STORM^19^. FluoSim-trained Deep-STORM network was able to faithfully reconstruct super-resolved maps of both simulated and experimentally-observed microtubules, from single molecule localization image stacks (Figs. S3-S4). Strikingly, the CNN worked well at both low and high single molecule density, thus offering a considerable gain in acquisition time (× 20) for an equivalent resolution^29^. FluoSim-trained Deep-STORM network also allowed the reconstruction of images of Nrx1β-Nlg1 contacts that were comparable to images obtained with PALM-Tracer^31^, or to images directly simulated by FluoSim (Fig. S5). FluoSim can therefore be used to train deep CNNs.

### Simulations of FRAP experiments

**Figure 4:**
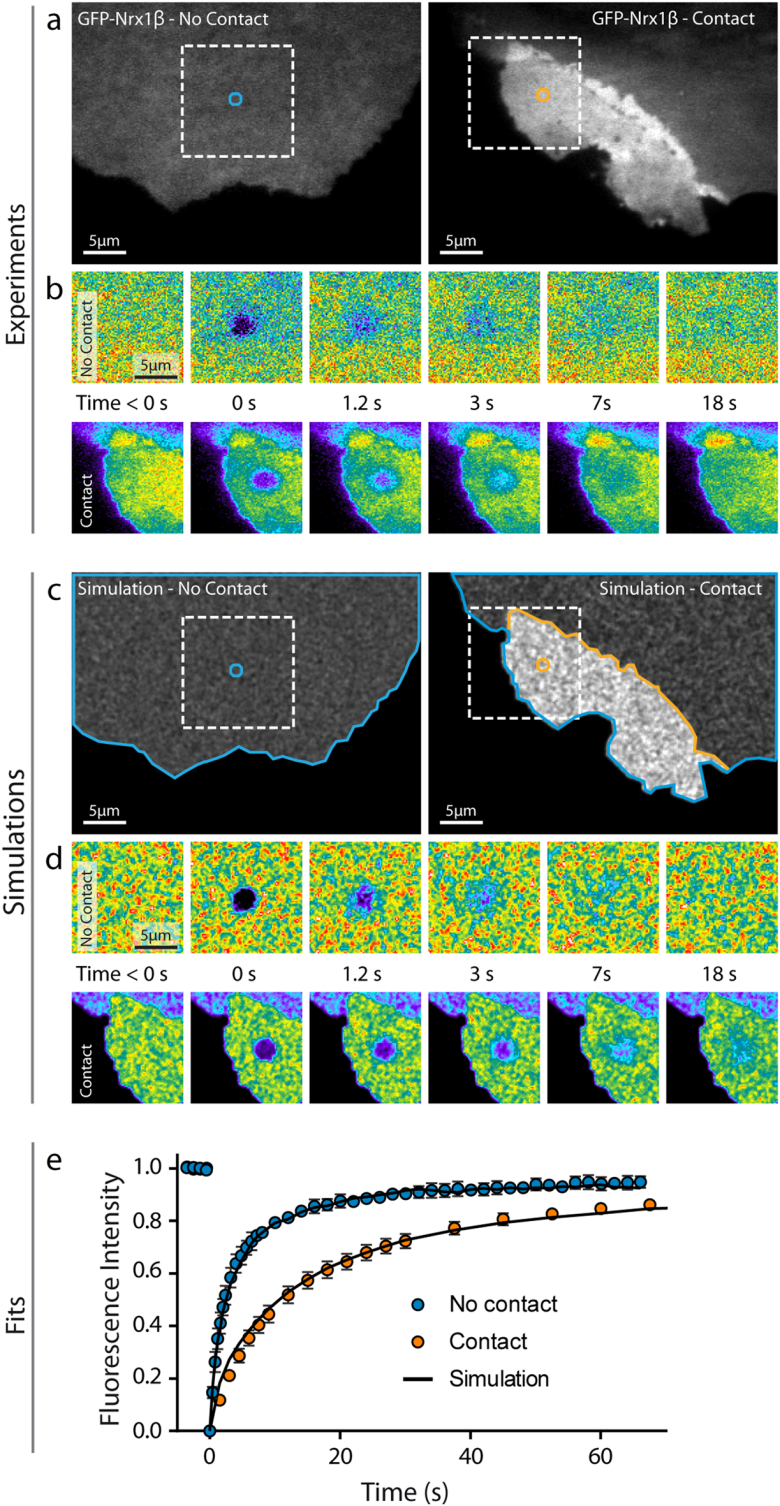
Fitting FRAP experiments. (**a**) Representative images of COS-7 cells expressing GFP-Nrx1β either not forming contact (left), or forming contact with another cell expressing Nlg1-mCherry (right). (**b**) Corresponding FRAP sequences on the zoomed square areas (bleached circle diameter = 2.8 µm). Intensity is color coded. (**c**,**d**) Simulated images of the same cell geometries filled with 150,000 fluorescent molecules, and corresponding FRAP sequences. (**e**) Normalized FRAP curves obtained by experiment (closed circles) outside (mean ± sem of 6 cells, 44 bleached regions) or inside contact regions (mean ± sem of 18 cells, 144 bleached regions) and corresponding simulations (solid curves, average of 30 repetitions each, sem < 1% mean, not shown). The Spearman correlation coefficient comparing experiment and simulation was r = 1.0 (*P* < 0.001, n = 21 time points) for contact regions, and r = 0.98 (*P* < 0.001, n = 43 time points) for outside regions.

To challenge the simulator against ensemble measurements, we performed FRAP experiments on GFP-Nrx1β expressed in COS-7 cells. GFP-Nrx1β accumulates at cell–cell contacts when the opposite cell expresses its molecular partner Nlg1 (Fig. 4a) and shows slower recovery in the adhesive contact as compared to membrane regions not in contact with other cells (Fig. 4b,e). To mimic these experiments, we introduced a large number of molecules in the simulator (up to 150,000) and generated fluorescence-like images by defining a Gaussian intensity profile for each GFP-tagged molecule (σ = 0.17 µm, FWHM = 0.47 µm). Taking kinetic parameters from the literature (k_on_ = 0.15 s^−1^ and k_off_ = 0.015 s^−1^) and an intermediate porosity (*P*_*crossing*_ = 0.3), the simulated images at steady state predicted Nrx1β enrichment in the contact area that matched experimental values (Fig. 4c,d). To induce local photo-bleaching, we chose a bleaching rate (k_off_^Bleach^ = 4.25 s^−1^) reproducing the initial drop of fluorescence observed experimentally (~ 75% in 400 ms). Using those coefficients plus the diffusion parameters determined from SPT, FRAP simulations accurately reproduced experimental data (Fig. 4e).

### Simulations of PAF experiments

**Figure 5:**
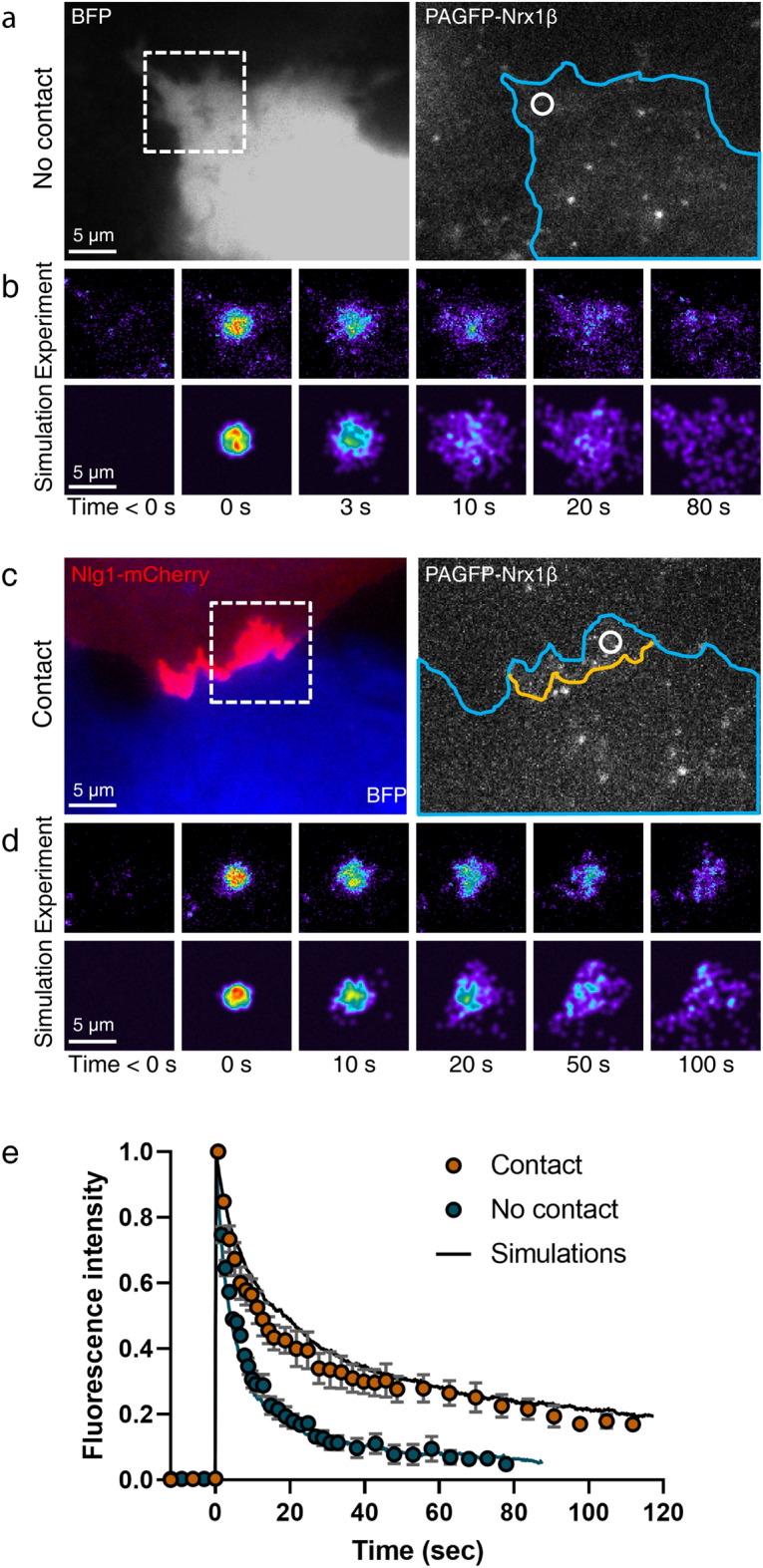
Fitting PAF experiments. (**a**,**c**) Representative images of COS-7 cells co-expressing BFP (blue) plus PAGFP-Nrx1β, and either not forming contact, or forming contact with another cell expressing Nlg1-mCherry (red). The cell geometry (blue contour) is entered in the simulator, together with the contact region (yellow line). (**b**,**d**) Corresponding PAF sequences on zoomed areas (dashed squares), where the photoactivated spot is indicated in white (circle of diameter = 2.8 µm). The experimental and simulated images are shown on top of each other for the indicated time points. Fluorescence intensity is color coded. (**e**) Graph showing the fluorescence intensity normalized between 0 and 1 for PAF experiments performed outside (blue circles) or inside (orange circles) contact regions (mean ± sem of 5 cells/1 PAF region per cell, and 3 cells/2 PAF regions per cell, respectively). The corresponding simulations (solid curves) represent the average of 20 repetitions (sem < 2% mean, not shown). The Spearman correlation coefficients comparing experiment and simulation was r = 0.998 for contact regions, and r = 0.995 for outside regions (*P* < 0.0001, n = 32 time points in both conditions).

PAF, which can be viewed as the mirror of a FRAP experiment, is also very popular to estimate membrane molecule dynamics in specific cellular compartments^32^. We thus confronted FluoSim with actual PAF experiments performed on COS-7 cells expressing Nrx1β fused to photoactivatable GFP (PAGFP-Nrx1β). Because PAGFP is non-fluorescent in the absence of photo-stimulation, cells were co-electroporated with BFP for detection. Characteristic adhesive contacts were formed between cells expressing PAGFP-Nrx1β, and co-cultured cells expressing Nlg1-mcherry (Fig. 5a,c). PAGFP-Nrx1β was turned on with a brief pulse of 405 nm laser and the fluorescence decay due to protein diffusion and turnover was recorded for 2 min (Fig. 5b,d). As expected from SPT and FRAP data, the fluorescence decay was slower for PAGFP-Nrx1β making contact with Nlg1-mcherry, compared to freely diffusing PAGFP-Nrx1β outside contact regions (Fig. 5e). In FluoSim, we introduced similar numbers of molecules, diffusion coefficients and kinetic rates as for FRAP. To match the sudden increase in PAGFP-Nrx1β fluorescence intensity at time zero, the photo-activation rate was raised from 0 to 1 s^−1^ for 300 ms. Given this set of parameters, the PAF simulations fit very well the experimental data (Fig. 5e).

### Simulations of FCS experiments

**Figure 6:**
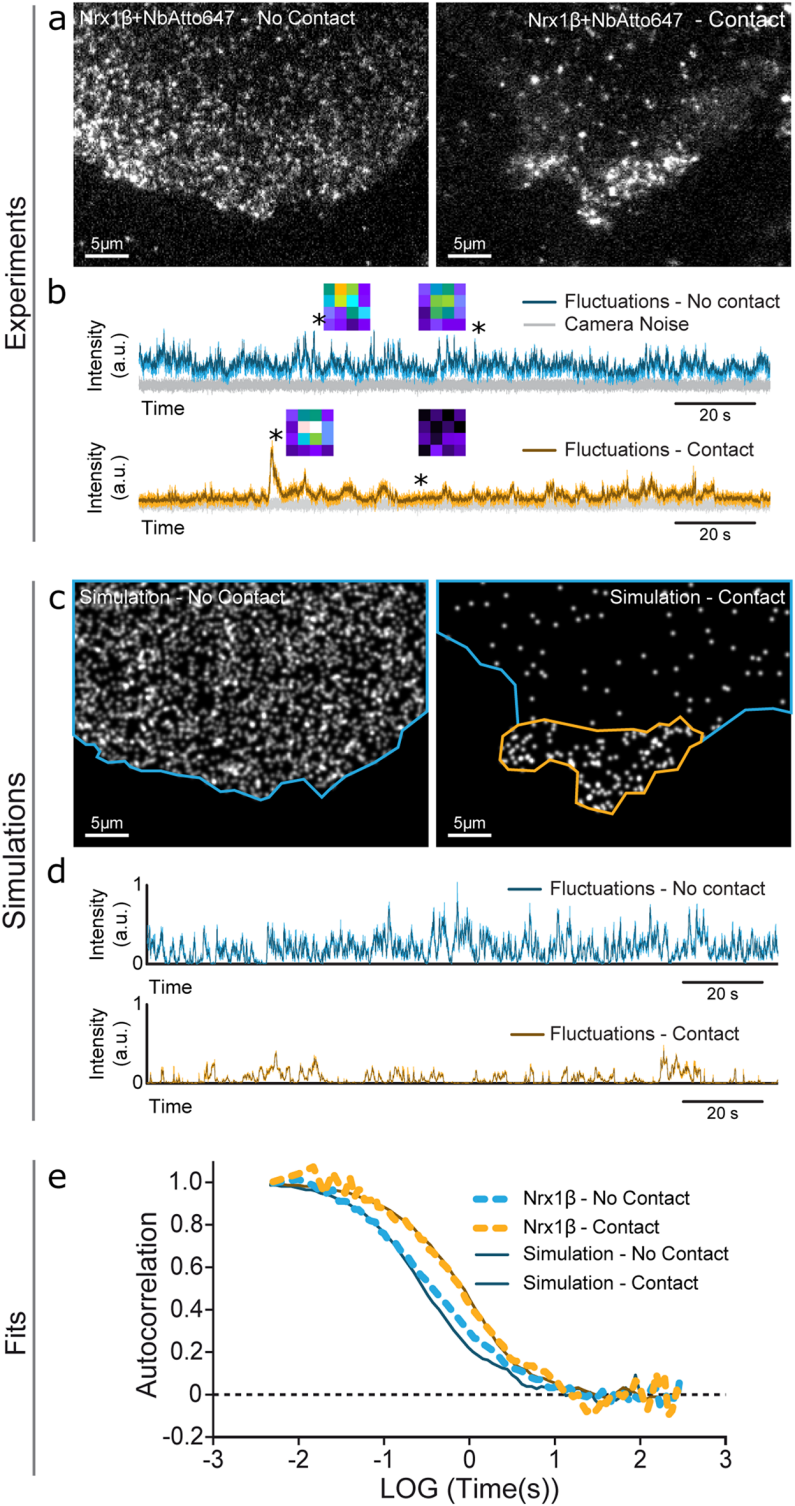
Fitting FCS experiments. (**a**) Images of Atto647N-conjugated GFP nanobody bound to COS cells expressing GFP-Nrx1β not forming contact (left), or forming contact with a cell expressing Nlg1-mCherry (right). A 642 nm focused laser beam of 0.6 µm FWHM was parked in contact or no-contact regions, and square images of the illuminated area (1 µm × 1 µm) were collected at 200 or 500 Hz, respectively. (**b**) Intensity fluctuations over time in the two regions (color), above the camera noise (grey). Insets represent images acquired at the indicated times (stars). Intensity is color coded. (**c**,**d**) Simulated images using the same cell geometries populated with 2500 and 200 molecules, respectively, and corresponding intensity fluctuations. (**e**) Normalized autocorrelation functions for FCS experiments (dashed curves) performed outside (2 cells/10 recordings) and inside contact regions (1 cell/3 recordings), and corresponding simulations (solid curves, average of 10 repetitions each).

To further validate the simulator in conditions of intermediate molecular density, we performed FCS experiments^33^ by recording intensity fluctuations over time for Atto647N-nanobody bound to GFP-Nrx1β, in a diffraction-limited laser spot (Fig. 6a,b). As expected from slower diffusion, the autocorrelation function obtained for Nrx1β in the adhesive contact was shifted to the right compared to the free region, with a small photo-bleaching bias due to longer residence times (Fig. 6e). Again, the simulations performed with an intermediary number of molecules introduced in FluoSim, matched experimental FCS curves with the same set of kinetic and photophysical parameters used for SPT, FRAP, and PAF (Fig. 6c–e).

## Discussion

In summary, FluoSim allows a prediction and comparison of membrane protein dynamics in a wide range of fluorescence cell imaging modes, with a precise control of the relevant kinetic, photo-physical, and acquisition parameters determining measurement outputs. The program is intended to help biologists adjust and interpret their experiments on a variety of cellular systems, and serve as a teaching resource in bio-imaging programs.

FluoSim reproduced a wide range of experimental results (SPT, FRAP, PAF, FCS, SRI) on the Nrx1β-Nlg1 membrane complex using a unique set of parameters extracted from published in-vitro studies and/or taken from our own measurements^26,27^, thereby giving strong credit to the correlative approach. The program is very fast and robust, and should be applicable to model a wide range of 2D-like dynamic molecular systems experiencing membrane diffusion and transient confinement, for example integrins at focal contacts in fibroblasts^34,35^, cadherins at cell–cell contacts^36–38^, neuronal adhesion proteins and neurotransmitter receptors at synapses^24,39,40^, and trapping of membrane molecules by lipid rafts or cytoskeletal interactions^41–43^.

Independently of changes in the fluorescence parameters, FluoSim can also be used to predict changes in the sub-cellular distribution of membrane proteins over time, induced by the modification of diffusion coefficients (D_out_, D_in_, D_trap_) or kinetic rates (k_on_, k_off_). For example, the effect of cross-linking surface receptors with antibodies can be simulated by a decrease in diffusion coefficients^44,45^. Moreover, changes in the parameters k_on_ and k_off_ in FluoSim can mimic the action of pharmacological agents, competing peptides, and chemo- or opto-genetic stimulations that modulate specific protein–protein interaction rates. For instance, cell-permeant peptides competing with glutamate receptor anchorage to scaffolding proteins rapidly decrease receptor density at synapses^46^, a process that is mimicked by changing the binding rates of receptors to their post-synaptic scaffold^45^. Similarly, long term potentiation (LTP) and long term depression (LTD) can be simulated by modulating the AMPA receptor/scaffold affinity, providing insight on the molecular mechanisms underlying synaptic plasticity^45,47^.

We believe that this quantitative simulation approach will be of great help to optimize experimental design, especially regarding the choice of various parameters such as fluorophores, control of laser powers, acquisition frame rate, and overall timing of the experiment with respect to the internal dynamics of the molecular system, thus replacing lengthy experimental adjustments and saving research time. The software can also be used to test the robustness and predictions of single molecule tracking algorithms that have been implemented those past years, e.g. SR-Tesseler and InferenceMap^48–50^. Compared to existing software such as PyFRAP, SuReSim, FERNET, or MCell^13–15^, FluoSim integrates many fluorescence modalities into a single program and achieves real-time display (Supplementary Table 1). In its present version, FluoSim is limited to Brownian motion and first order molecular reactions, but sub- or super-diffusive behaviors as well as more complex multi-state molecular reactions might be implemented on a case-by-case basis, depending on user needs (Table 1).

**Table 1:** Simulator parameters.

Category	Parameter	Notation	Unit/format
Geometry	Reference image		.TIFF, PNG, JPG, GIF
	Image pixel size		µm/pixel
	Region file		.rgn or .roi
	FRAP laser spot		2.8 µm
	FCS laser spot width	σ_FCS_	0.25 µm
Molecules	Number^a^		1–150,000
	Initial position		Uniform or estimated
Times	Simulation duration^a^		1000–100,000 frames (40–1600 s)
	Time step	*Δt*	2–100 ms
Diffusion coefficients	Outside contact	*D*_*out*_	0.3 µm^2^/s
	Inside contact	*D*_*in*_	0.3 µm^2^/s
	Trapped	*D*_*trap*_	0.04 µm^2^/s
	Crossing probability	*P*_*crossing*_	25–70%
Kinetics	Binding rate	*k*_*on*_	0.15 s^−1^
	Unbinding rate	*k*_*off*_	0.015 s^−1^
Photophysics	Switch-on rate^a^	*k*_*on*_^*Fluo*^	0.005–5 s^−1^
	Switch-off rate^a^	*k*_*off*_^*Fluo*^	0–10 s^−1^
	Bleaching rate^a^	*k*_*off*_^*Bleach*^	0.4–4 s^−1^
	Activation rate^a^	*k*_*off*_^*Activ*^	1–2 s^−1^
Export files	SPT or SRI images		.TIFF or multi-TIFF
	SPT trajectories		.trc
	SPT histogram		.txt
	FRAP curves		.txt
	FCS fluctuations and autocorrelation		.txt

^a^See the methods above for the specific molecule numbers and photo-physical parameters used in the various imaging modes (SPT, STORM, FRAP, PAF, FCS).

Finally, with the advent of single-molecule based super-resolution microscopy such as PALM and STORM, many new questions arise regarding the degree of labeling needed, and the number of single molecule localizations to accumulate in order to reconstruct a realistic image of biological structures, with the risk of finding artificial properties in under-sampling conditions^10^. By varying the number of molecules, type of fluorophore attached to them, localization precision, and length of the acquisition sequence, the software is able to determine the conditions for faithful detection of the biological sample. The capacity of FluoSim to mimic molecule dynamics might also be used in the near future to train the next generation of CNNs for the analysis of live imaging experiments^51^.

## Methods

### DNA plasmids

GFP-Nrx1β was a gift from M. Missler (Münster University, Germany). PA-GFP-Nrx1β was generated by amplifying PA-GFP^32^ (a gift of J. Lippincott-Schwartz, Janelia Research Campus, USA) with the two primers PAGFP-3F (5′-cgGCTAGCGGAGCAGGAatggtgagcaagggcgagg-3′) and PAGFP-4R (5′-cgGCTAGCTCCTGCTCCCTTGTACAGCTCGTCCATGC-3′) and inserting it into GFP-Nrx1β in place of GFP at NheI/NheI sites. Proper insert orientation was verified by restriction and sequencing. HA-tagged Nlg1 obtained from P. Scheiffele (Biozentrum, Basel) was used as a backbone to construct Nlg1-mCherry, by inserting mCherry intracellularly at position -21aa before the C-terminus. The HA-Nlg1 sequence was moved from the pNice vector into a pcDNA vector at the HindIII/ NotI sites. Two PCRs were performed on Nlg1: the first one from the KpnI site (located inside the Nlg1 sequence) to the insert position of mCherry (AgeI site added), and the other one from the mCherry site insertion (Nhe added) to the end of Nlg1 (NotI). The mCherry gene (AgeI/NheI) was obtained by PCR on pmCherry-N1 (Clontech). A four-fragment ligation was then done to obtain the final construct (HindIII-HA-Nlg1-AgeI-mCherry-NheI-Nlg1Cter-NotI). The plasmid for bacterial expression of the anti-GFP nanobody^52^ was a kind gift from A. Gautreau (Gif-sur-Yvette, France). The bacterial production of anti-GFP nanobody, purification, and conjugation to organic dyes (Atto647N or Alexa647), was described previously^24^.

### Cell culture and electroporation

COS-7 cells (ATCC) were cultured in Dulbecco's modified Eagle's medium (DMEM; GIBCO/BRL) supplemented with 10% fetal bovine serum (FBS), 100 units mL^−1^ penicillin, and 100 µg mL^−1^ streptomycin, in a 37 °C-5% CO_2_ atmosphere. One day before the experiments, cells were rinsed twice in warm PBS, trypsinized for 5 min, mixed in culture medium, and centrifuged for 5 min at 1000 rpm. The cell pellet was suspended in 100 µL electroporation medium and electroporated for either GFP-Nrx1β or NLG1-mCherry plasmids with the Amaxa Nucleofector system (Lonza), using 500,000 cells per cuvette and 3 µg DNA. Electroporated cells were mixed in culture medium, seeded on 18 mm glass coverslips at a concentration of 50,000–80,000 cells per coverslip, cultured in 12-well plates, and imaged 24–48 h after electroporation.

### Single molecule pull-down

Single molecule pull-down of GFP-Nrx1β molecules was performed essentially as described^53,54^. Briefly, cleaned glass coverslips (VWR) were treated with N-(2-aminoethyl)-3-aminopropyltrimethoxysilane (United Chemical Technologies, #A0700), then incubated with mPEG-succinimidyl valerate (SVA) containing a 1:100 fraction of biotin PEG-SVA (both from Laysan Bio MW 5000 Da). Coverslips were dried and stored at − 20 °C. Just before the experiment, coverslips were incubated with 1 µM NeutrAvidin for 5 min (Invitrogen, A2666), followed by 10 nM biotinylated anti-GFP antibody for 15 min (ABCAM, 6658), both diluted in T50 buffer (10 mM Tris-HCl, 50 mM NaCl, pH = 7.5). Two days before the experiment, HEK cells were transfected with GFP-Nrx1β using Lipofectamine (Invitrogen) in optiMEM buffer. Two hours before the experiment, cells were detached in Ca-free PBS (30 min, 37 °C), then centrifuged at 3000 rpm for 2 min. The cell pellet was dissolved in 100 μL lysis buffer containing 150 mM NaCl, 10 mM Tris-HCl, 1 mM EDTA, 1% Igepal, and protease inhibitor cocktail (Pierce). Samples were rotated for 2 h at 4 °C to solubilize proteins, then centrifuged at 14,000 rpm for 15 min to remove aggregates. The supernatant was kept on ice and diluted 1:200 in observation solution (135 mM NaCl, 5 mM KCl, 2 mM CaCl_2_, 1 mM MgCl_2_, 10 mM HEPES, 4 mM Trolox, 40 mM d-glucose, 0.03% Igepal, pH = 7.4). 100 µL of this mix was added to glass substrates for several minutes, then rinsed out with fresh solution when the molecular density reached about 100 per field of view (65 × 65 µm). Single GFP-Nrx1β molecules were observed with a 488 nm laser under TIRF illumination on an inverted Olympus microscope equipped with a 63 ×/1.45 NA objective and an EMCCD camera (Andor Ixon). GFP-Nrx1β molecules did not stick to a control coverslip containing no anti-GFP antibody, revealing binding specificity to the GFP tag. Acquisition sequences of 500 frames were recorded at 30 Hz, resulting in the photobleaching of nearly all immobilized GFP-Nrx1β molecules. Images were analyzed using Metamorph by measuring the average fluorescence intensity in small regions drawn around individual molecules.

### Single molecule tracking (uPAINT)

Universal point accumulation in nanoscale topography (uPAINT) experiments were carried out as reported^8^. Cells were mounted in Tyrode solution (15 mM d-glucose, 108 mM NaCl, 5 mM KCl, 2 mM MgCl_2_, 2 mM CaCl_2_ and 25 mM HEPES, pH 7.4) containing 1% globulin-free BSA (Sigma A7638) in an open Inox observation chamber (Life Imaging Services, Basel, Switzerland). The chamber was placed on a fully motorized inverted microscope (Nikon Ti-E Eclipse) equipped with perfect focus system, a thermostatic box (Life Imaging Services) providing air at 37 °C, and an APO TIRF 100 ×/1.49 NA oil immersion objective. GFP-and mCherry expressing cells were detected using a mercury lamp (Nikon Xcite) and the following filter sets (SemROCK): EGFP (Excitation: FF01-472/30; Dichroic: FF-495Di02; Emission: FF01-525/30) and mCherry (Excitation: FF01-543/22; Dichroic: FF-562Di02; Emission: FF01-593/40). Cells expressing GFP-Nrx1β were labeled using a low concentration of Atto647N-conjugated GFP nanobody (1 nM). A four-colour laser bench (405/488/561 nm lines, 100 mW each; Roper Scientific, and 1 W 642 nm line, MPB Communications Inc., Canada) is connected through an optical fiber to the Total Internal Reflection Fluorescence (TIRF) illumination arm of the microscope. Laser power was controlled through an acousto-optical tunable filter (AOTF) driven by the Metamorph software (Molecular Devices). Atto647N was excited with the 642 nm laser line (~ 2 mW at the objective front lens), through a four-band beam splitter (BS R405/488/561/635, SemRock). Samples were imaged by oblique laser illumination, allowing the excitation of individual Atto-conjugated ligands bound to the cell surface, without illuminating ligands in solution. Fluorescence was collected on an EMCCD camera with 16 µm pixel size (Evolve, Roper Scientific, Evry, France), using a FF01-676/29 nm emission filter (SemRock). Stacks of 4000 consecutive frames were obtained from each cell with an integration time of 20 ms, using the Nikon perfect focus system to avoid axial drift. Images were analyzed using PALM-Tracer, a program running on Metamorph and based on wavelet segmentation for molecule localization and simulated annealing algorithms for tracking (generously provided by JB Sibarita, Bordeaux)^55^. This program allows the tracking of localized molecules through successive images. Trajectories longer than 20 frames (400 ms) were selected. The diffusion coefficient, D, was calculated for each trajectory, from linear fits of the first 4 points of the mean square displacement (MSD) function versus time. Trajectories with displacement inferior to the pointing accuracy (~ 50 nm in uPAINT conditions) whose MSD function cannot be fitted are arbitrarily taken as D = 10^−5 ^µm^2^ s^−1^.

### dSTORM experiments

COS-7 cells expressing GFP-Nrx1β were surface-labeled with a high concentration (100 nM) of Alexa647-conjugated GFP Nanobody in Tyrode solution containing 1% globulin-free BSA (Sigma A7638) for 10 min, rinsed and fixed with 4% PFA-0.2% glutaraldehyde in PBS for 10 min at room temperature, and stored in PBS at 4 °C until imaging. For microtubule staining, COS-7 cells were rinsed twice in PBS, fixed using 4% PFA-20% sucrose for 15 min at room temperature, washed 3 times in PBS, and incubated with 50 mM NH_4_Cl in PBS for 10 min. After fixation, cells were washed 3 times in PBS, permeabilized using 0.2% Triton-X 100 for 10 min, washed in PBS, blocked in 1% BSA-PBS for 30 min, and incubated with anti-α-tubulin (Thermofisher MA1-80017, 1:500) overnight at 4 °C. The next day, cells were washed 3 times in PBS, and incubated 45 min with secondary goat-anti-rat-Alexa647 antibody (ThermoFisher A21247, 1:800) and kept in PBS before dSTORM imaging.

Cells were imaged in Tris‐HCl buffer (pH 7.5) containing 10% glycerol, 10% glucose, 0.5 mg/mL glucose oxidase (Sigma), 40 mg/mL catalase (Sigma C100-0.1% w/v), and 50 mM β-mercaptoethylamine (MEA) (Sigma M6500)^56^. The same microscope described for uPAINT was used. Pumping of Alexa647 dyes into their triplet state was performed for several seconds using ~ 60 mW of the 642 nm laser at the objective front lens. Then, a lower power (~ 30 mW) was applied to detect the stochastic emission of single-molecule fluorescence, which was collected using the same optics and detector as described above for uPAINT. 10–20 streams of 3000–4000 frames each were acquired at 50 Hz. To generate images intended to test deep CNN algorithms, a higher density of fluorescent molecules was generated by turning on the 405 nm laser power to 5 mW during acquisition. Multi-color 100-nm fluorescent beads (Tetraspeck, Invitrogen) were used to register long-term acquisitions and correct for lateral drift. The localization precision of our imaging system in STORM conditions is around 60 nm (FWHM). Stacks were analyzed using the PALM-Tracer program, allowing the reconstruction of a unique super-resolved image of 32 nm pixel size (zoom 5 compared to the original images) by summing the intensities of all localized single molecules (1 detection per frame is coded by an intensity value of 1).

### FRAP experiments and analysis

COS-7 cells expressing GFP-Nrx1β in co-culture with cells expressing Nlg1-mCherry were mounted in Tyrode solution, and observed under the same set-up used for uPAINT and STORM. The laser bench has a second optical fiber output connected to an illumination device containing two x/y galvanometric scanning mirrors (ILAS, Roper Instrument) steered by MetaMorph. It allows precise spatial and temporal control of the focused laser beam at any user-selected region of interest within the sample for targeted photo-bleaching. Switching between the two fibers for alternating between imaging and bleaching is performed in the ms time range using an AOTF. Oblique illumination was performed using the 491 nm beam at low power (0.3 mW at the front of the objective) to image GFP-Nrx1β molecules in the plasma membrane close to the substrate plane. After acquiring a 10 s baseline at 1 Hz frame rate, rapid selective photo-bleaching of 3–9 circular areas of diameter 2.8 µm was achieved at higher laser power (3 mW at the objective front lens), during 400 ms. Fluorescence recovery was then recorded immediately after the bleach sequence for 80 s. The recording period included three phases with decreasing frame rate ranging from 2 to 0.1 Hz. Observational photo-bleaching was kept very low, as assessed by observing control unbleached areas nearby. FRAP curves were obtained by computing the average intensity in the photobleached area, after background subtraction, and normalized between 1 (baseline) and 0 (time zero after photo-bleaching).

### PAF experiments and analysis

COS-7 cells co-expressing BFP and PAGFP-Nrx1β in co-culture with cells expressing Nlg1-mCherry were mounted in Tyrode solution, and observed under the microscope as described for FRAP experiments. Oblique illumination was carried out using the 491 nm beam at low power (0.3 mW at the front of the objective) to image membrane PAGFP-Nrx1β molecules close to the substrate. After acquiring a 12 s baseline at 0.3 Hz frame rate, photo-activation of 1–2 circular areas of diameter 2.8 µm was achieved with the focused 405 nm laser beam (1.5 mW at the objective front lens), during 300 ms. Fluorescence decay was then recorded for 80–100 s, with decreasing frame rate ranging from 2 to 0.1 Hz. PAF curves were normalized between 0 and 1, by dividing the intensity in the photoactivated area over time by the maximal intensity recorded immediately after the photo-activation pulse, after subtracting the baseline from both values.

### FCS experiments and analysis

COS-7 cells expressing GFP-Nrx1β were surface-labeled at an intermediate concentration (10 nM) of Atto647N-conjugated GFP nanobody for 5 min at 37 °C, then mounted in Tyrode solution on the same microscope. The 642 nm laser beam was parked on a region of interest, either on the free cell membrane, or in the cell–cell contact area. The laser was kept at low power (3% of 100 mW, i.e. ~ 0.2 mW at the objective front lens) to avoid photo-bleaching the organic dye. Using glass coverslips coated with polylysine and higher concentrations of Atto647N-conjugated nanobody (100 nM) to provide a uniform density, we independently measured the Gaussian intensity profile of the laser beam, which gave a standard deviation σ_FCS_ = 0.26 µm (FWHM = 0.6 µm). Intensity fluctuations due to Nrx1β molecules entering and leaving the laser spot by membrane diffusion, were detected using a second camera (Hamamatsu Orca Flash 4.0) on the opposite port of the microscope. A square area of 16 × 16 pixels (1 µm × 1 µm) centered on the laser spot was chosen, and streams of 60,000 images at binning 4 were acquired through the Hokawo software (Hamamatsu) at 200 Hz or 500 Hz, for the study of cell–cell contacts or free regions, respectively. Control stacks with the laser off were acquired to record the camera readout noise. The integrated intensity of each image was read from the multi-TIFF stacks, from which the average camera noise was subtracted, and the autocorrelation function was computed using custom-made routines written in C++, and normalized by its first value.

### FluoSim simulations

A general description of the FluoSim algorithm and the list of simulations performed to match the experiments, together with the precise set of parameters used in each imaging mode, is available in the Supplementary material.

## Supplementary information

Supplementary Information 1.

Supplementary Information 2.

Supplementary Information 3.

Supplementary Information 4.

## Data Availability

The authors declare that all data supporting the findings of this study are available within the paper and its supplementary information files. The software FluoSim (v1.0) is released under a GNU GPL v3 license as supplementary material accompanying this manuscript, and as an archive that can be downloaded at https://www.iins.u-bordeaux.fr/SOFTWARE. The FluoSim (v1.0) release and its source code are available at https://github.com/mlagardere/FluoSim under the same license.
