# Supplementary material for: FluoSim: simulator of single molecule dynamics for fluorescence live-cell and super-resolution imaging of membrane proteins
Source: Sci Rep. 2020 Nov 17;10:19954. doi: 10.1038/s41598-020-75814-y (PMC7672080; doi:10.1038/s41598-020-75814-y)
Supplement: Supplementary file 1 — Supplementary Information 1. [file 41598_2020_75814_MOESM1_ESM.pdf]

FluoSim

Title: FluoSim: simulator of single molecule dynamics for fluorescence live-cell and super-resolution imaging of membrane proteins

Authors: Matthieu Lagardère[1], Ingrid Chamma[1], Emmanuel Bouilhol[2], Macha Nikolski[2], Olivier Thoumine[1]*

*Corresponding author: olivier.thoumine@u-bordeaux.fr

Affiliations:

1. Univ. Bordeaux, CNRS, Interdisciplinary Institute for Neuroscience, IINS, UMR 5297, F-33000 Bordeaux, France
2. Univ. Bordeaux, CNRS, IBGC, UMR 5095, F-33000 Bordeaux, France

FluoSim User Manual

(Version 1.0 – September 21, 2020)

Design and development:

Matthieu Lagardère and Olivier Thoumine
Interdisciplinary Institute for Neuroscience (UMR 5297)
CNRS - University of Bordeaux
France

matthieu.lagardere.pro@gmail.com

olivier.thoumine@u-bordeaux.fr

Table of contents

1. About FluoSim 5
2. Program installation and execution 5
3. Program overview 6
3.1 File format 6
3.2 Interface overview 7
4. Defining cellular geometry and sub-cellular regions 10
4.1 Cellular modeling 10
4.2 Importing cell images 11
4.3 Importing existing geometries 12
4.4 Drawing a new geometry 12
4.5 Managing geometries 13
4.6 Simulation Length Scale 13
5. Entering molecules 14
5.1 Setting the number of molecules 14
5.2 Reaching steady state 14
6. Image rendering 15
6.1 Geometry and image rendering 15
6.2 Particle rendering 15
7. Kinetic parameters 16
7.1 Diffusion-trapping model 16
7.2 Kinetic interface 17
8. Simulation progress 18
8.1 Simulation progress interface 18
8.2 Simulation Time Step 18
9. Photophysics 19
9.1 Photophysics model 19
9.2 Photophysics applications 19
10. Plotting system 20
11. Live Experiments 20
11.1 FRAP 20
11.2 PAF 21
12. Recorded experiments 22

12.1 General experimental parameters 22
12.2 Simulation outputs 23
12.3 SPT experiments 25
12.4 FRAP experiments 26
12.5 PAF experiments 27
12.6 FCS experiments 28
12.7 SRI experiments 29
12.8 DRUG experiments 30
13. Analysis of SPT experiments 31
13.1 Importing trajectories 31
13.2 Trajectory rendering 32
13.3 Trajectory filtering 32
13.4 Advanced parameters 32
13.5 Exporting Data 33
14. FluoSim projects 34
15. Examples 34
15.1 SPT Experiment 34
15.3 FRAP experiment 38
15.4 PAF experiment 40
15.5 FCS Experiment 43
15.6 SRI Experiment 46
15.7 Single molecule pull-down 49
15.8 LTP 51
16. Rights to use and code accessibility 53

FluoSim

1. About FluoSim

FluoSim is a computer software with a graphic user interface designed to mimic and quantitatively analyze fluorescence microscopy experiments. FluoSim is built around a core algorithm which uses Monte Carlo methods and agent-based modeling, to perform high speed calculations that update the coordinates and fluorescence intensities of thousands of molecules in real time, within virtual 2D geometries. FluoSim is capable of simulating a wide range of fluorescence live cell imaging experiments such as Single Particle Tracking (SPT), Fluorescence Correlation Spectroscopy (FCS), and ensemble measurements like Fluorescence Recovery After Photobleaching (FRAP) or PhotoActivation of Fluorescence (PAF) experiments, as well as Super-Resolution Imaging (SRI) such as PALM or STORM. FluoSim is intended to help biologists in the design, prediction and analysis of their experiments, and to serve as a teaching resource in bio-imaging courses.

To download a high definition movie demonstrating the various imaging modalities of FluoSim, please go to the following website: http://www.iins.u-bordeaux.fr/SOFTWARE

Key words: Monte Carlo Methods, Brownian Dynamics, Diffusion-Trapping, Photophysics, Single Particle Tracking, Fluorescence Recovery After Photobleaching, Fluorescence Correlation Spectroscopy, Super Resolution Imaging.

2. Program installation and execution

The software is provided as a compressed file (FluoSim.zip). The archive contains the executable file (FluoSim.exe) which is used to start the software, the associated libraries (*.dll), and several folders containing either data to import (Cell Samples), application examples, or simulation output files. To install the program, just unzip the archive and place its content in a folder on your computer. A shortcut can be manually created and placed wherever it is convenient on your computer. To start FluoSim, you can double click on the manually created shortcut or the execution file contained in the installation folder (**FluoSim.exe**).

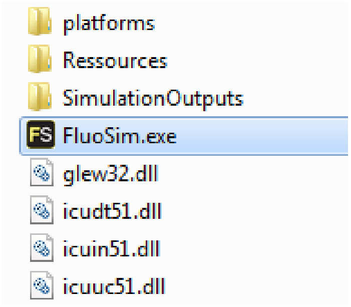

Figure 1 **FluoSim executable file**

Computer requirements

FluoSim is designed for personal computers equipped with Windows XP, Windows 7.0, or Windows 10 Operating Systems (OS). It runs on both 32- and 64-bits systems. The program is not compatible with MacOS or Linux.

A Graphics Processing Unit (GPU) compatible with OpenGL 3.2 is needed. Video cards (AMD or NVidia) released after 2007 are expected to be compatible. See web pages of AMD GPU or NVidia GPU for in depth information. Intel Graphic Chipsets released after 2012 should be compatible. A performing GPU is preferable for optimal performance.

A computer mouse with a scroll wheel to zoom in and out on the displayed image is recommended.

3. Program overview

3.1 File format

FluoSim uses several file formats to import and export data. These formats can be classified in 6 categories: geometry format, image format, project format, trajectory format, intensity format and histogram format.

3.1.1 Geometry Format

FluoSim can import two geometry file formats: the MetaMorph® (Molecular Devices) and the ImageJ geometry formats, but can only export the MetaMorph geometry format.

The **MetaMorph geometry file** (.rgn) is a plain ASCII text file which defines one geometry per line. These lines are divided into 8 sections separated by comas and labeled with integer values from 0 to 7. The most important sections are the first one (label '0') and the seventh (label '6'), which respectively define the type and the coordinates of the geometry in pixels. More information on this format can be found on the following web page: http://mdc.custhelp.com/app/answers/detail/a_id/19937/~/meta-imaging-series%C2%AE-region-file-format

The **ImageJ geometry format** is a binary file (.roi) and hence cannot be read or written with a text editor. The file starts with a 64 bytes long header and is followed by the coordinates of the geometry in pixels. For more information, the layout of the format and a decoder class written in java are given on the following webpage: https://imagej.nih.gov/ij/developer/source/ij/io/RoiDecoder.java.html
To export the current ROI in ImageJ, use "File/Save As/Selection…" accessible from the menu bar.

3.1.2 Image Formats

FluoSim can import the main image file formats: bmp, jpeg, tif, gif, png.

At the end of an SRI simulation, in the EXPERIMENT mode, FluoSim exports a super-resolved image containing all single molecule localizations in 16-bit depth tiff image. In any EXPERIMENT mode (SPT, FRAP, PAF, FCS, DRUG), FluoSim can export a stack of 16-bit depth images in MULTI-TIFF format, as a result of the simulation.

3.1.3 Trajectory Formats

FluoSim imports and exports trajectory files (.trc). Those files possess 6 tab-separated columns used to specify one or more trajectories. Trajectories are identified by a unique index. Each point of the trajectory has a Time Stamp indicating the frame in which it has been recorded. The coordinates of the trajectory points are expressed in pixels (px).

Column 1	Column 2	Column 3	Column 4	Column 5	Column 6
Trajectory Index [ ]	Time Stamp [frame]	X-coordinate [px]	Y-coordinate [px]	Unused [ ]	Intensity [a.u.]

3.1.4 FluoSim project format

FluoSim uses project files (.pro) to save and load simulation projects. They are plain ASCII text files and consist in pairs of key-values. The key indicates which property is affected by the associated value. For example, the following line can be found in a project file: "[simulationParams.pixel_size] 0.16". This

line states that the property "simulationParams.pixel_size" (the simulation pixel size) is equal to 0.160 µm.

Column 1	Column 2
Key	Value

Remark: the user is not expected to modify these files. But saving these files together with the simulated data is a convenient way to keep track of the parameters used during the simulation.

3.1.5 Other Formats

Average Intensities

FluoSim can export as a text file (.txt) the recorded intensities measured in the activated ROIs. This format uses two tab-separated columns, one representing the time and the other the average intensity.

Column 1	Column 2
Time Stamp [s]	Average Intensity [ ]

Correlogram

FluoSim can also export in a text file (.txt) the correlograms recorded during the FCS experiments. These correlograms possess two columns, one representing the time delay between the original signal and the translated signal in log scale, and a second column which contains the associated autocorrelation function.

Column 1	Column 2
Log(Time Delay [s])	Correlation [ ]

3.2 Interface overview

The FluoSim interface is constituted of a Menu Bar, a Drawing Panel, a Graphic Window, a Display Window and a Parameter Panel as shown in the figure below.

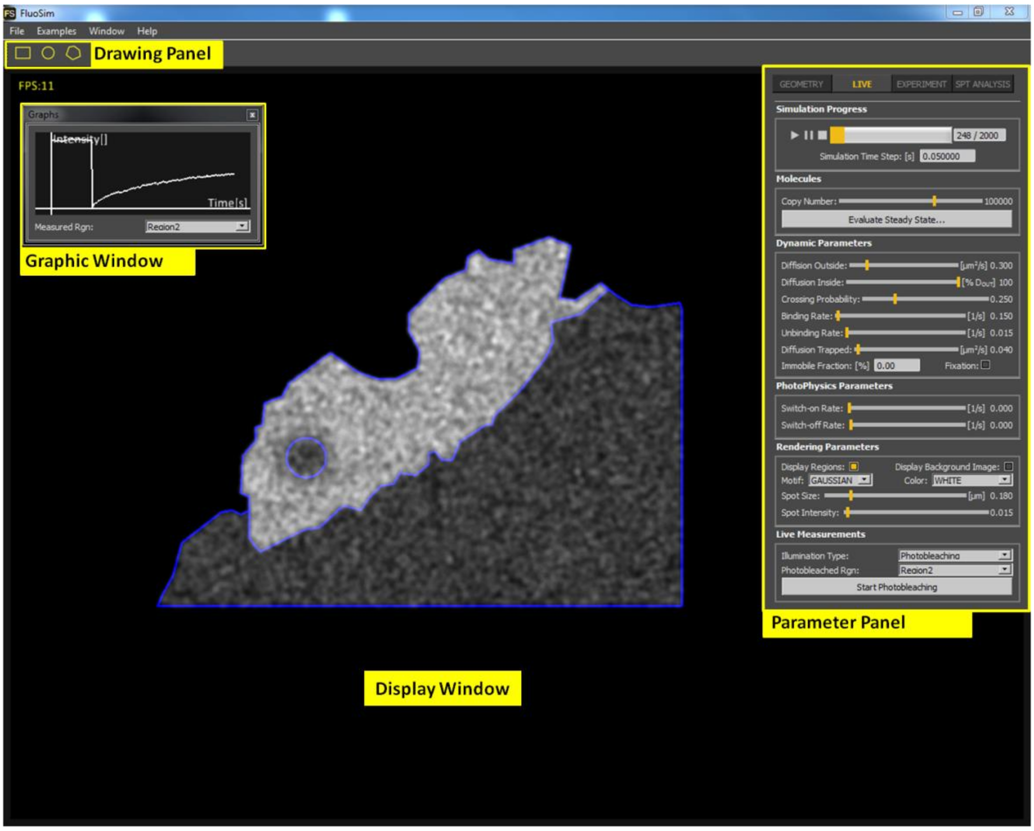

Figure 2 **General FluoSim Interface**

Menu Bar: is used to import or export projects, load examples, hide or display panels and access useful information about FluoSim. A function called Capture Screen saves an image of the Display window as a file called *screencapture.bmp* and is exported by default in the Simulation Output folder.

Drawing Panel: allows the user to draw geometries which will be used during the simulation. Three types of geometries are accessible: rectangle, ellipse and polygon.

Graphic Window: displays a plot which is updated in live mode. Any geometry can be used for measurements and the plot displays the average intensity (i.e. the number of molecules in the ROI divided by the surface area px^2), over time.

Display Window: is used to display the state of the simulation. This is an interactive panel: the user can zoom in/out and grab the window to change the field of view.

Parameter Panel: is made of 4 different tabs: GEOMETRY, LIVE, EXPERIMENT and SPT ANALYSIS which are shown respectively in Figures 2-5 below. Through these tabs, the user has access to the parameters of the simulation. These parameters will be described in the next sections.

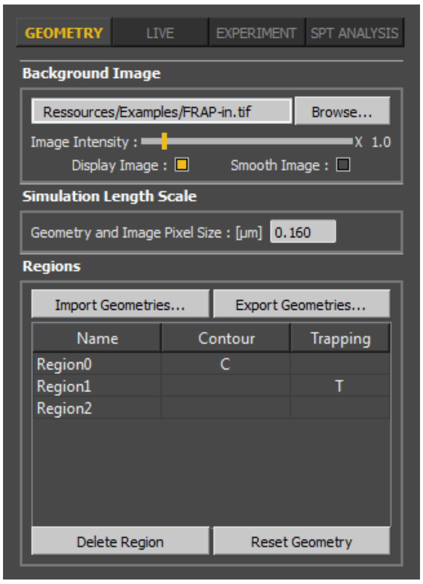

Figure 3 Parameter Panel - GEOMETRY Menu

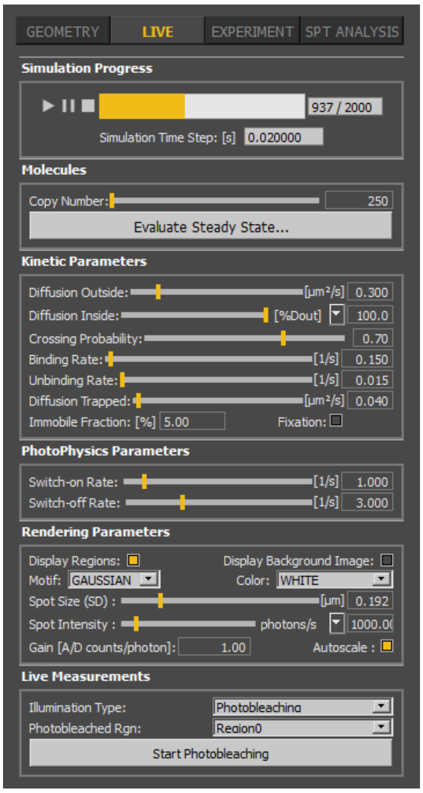

Figure 4 Parameter Panel - LIVE Menu

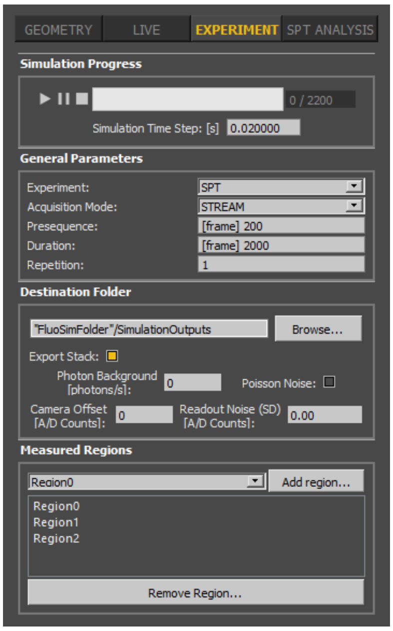

Figure 5 Parameter Panel - EXPERIMENT Menu

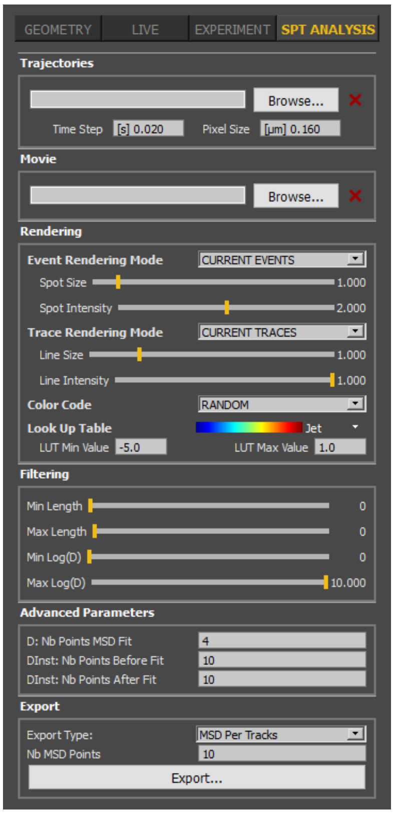

Figure 6 Parameter Panel - SPT ANALYSIS Menu

4. Defining cellular geometry and sub-cellular regions

4.1 Cellular modeling

4.1.1 FluoSim region types

FluoSim uses three types of region: a unique c**ontour region**, one or several **membrane compartments** and one or several **measurement regions**: regions of interest (ROIs) in which measurements can be performed.

The **Contour region** defines the overall outline of the cell. This region is not permeable to molecules i.e. they bounce on the border and hence are always confined inside the geometry. Far from the boundaries, molecules diffuse freely, and their diffusion coefficient is written D_{out} (Diffusion outside) to recall that they are outside of any specific membrane sub-compartment.

Remark: The Contour region (the first region in the "Regions" list of the geometry panel in FluoSim) contains all the molecules in the cell system and all other regions.

The **Membrane compartments** correspond to areas inside the Cell where molecules can interact with other molecular species, which usually slows down their mobility. Hence, when a molecule enters a membrane compartment its diffusion coefficient takes a new value called D_{in} (Diffusion inside). To model interactions (for example with scaffolding proteins or extracellular matrix components), membrane compartments have the ability to trap the molecules. Two kinetic rates are used to describe these interactions: the binding rate (k_{on}) and the unbinding rate (k_{off}), in units of sec^{-1}. Once a molecule is trapped, its coefficient of diffusion is set to D_{trap}. If D_{trap} is set to 0, then trapped molecules are immobilized, otherwise they continue to diffuse. Trapping regions are naturally permeable, although the probability to enter these regions can be reduced due to steric hindrance (see the **Crossing Probability** parameter).

Remark: Although regions can be drawn anywhere on the screen, even outside of the Contour region, they have to be entirely included in the Contour region since they are considered as cellular sub-compartments, and thus belong to the cell. Since molecules are not able to leave the Contour region, their dynamics will only be affected if membrane compartments are included in the Contour region.

The **Measurement regions** define regions used to get information from the system (intensity or positions of the molecules) or to perform photobleaching or photoactivation as in FRAP and PAF experiments, respectively. These regions do not modify the intrinsic dynamics of the molecules. They also have to be included in the Contour region, but they can be drawn anywhere within these boundaries, even at the crossing between a free region and a trapping region.

Remark: The contour and membrane compartments can also be used to perform measurements. If used so, they also keep their kinetic properties and hence still affect the movement of molecules.

4.1.2 Defining a Region Type

The type of a region is defined through the region table situated in the Geometry Tab. The region table has three columns: the first column specifies the region names, the second column indicates with the letter "C" the contour region, the third column indicates with the letter "T" if the region is a trapping region. No letter in both second and third column indicates that the region is not a cellular compartment, i.e. it is a basic region which has no effect on the molecular dynamics but can still be

used to perform measurements. The first region in the list is always the contour region. To change the Contour region, you have to delete all the regions in the list above the region you want to become the new Contour region. To delete a region, select it: it should now be highlighted (orange text and white background), and press the “Delete Region” button. To change the trapping state of a region, double click on the corresponding box, the letter ‘T’ will appear or disappear depending on the region state: ‘T’ if the region can trap molecules, or empty otherwise.

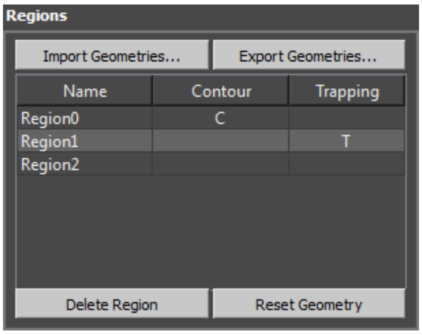

Figure 7 Region Panel

4.2 Importing cell images

FluoSim can import and display existing images. To import an image, click on the *Browse background image* button and select the directory of interest.

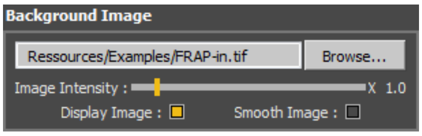

Figure 8 Background image panel

For example, you can choose in the “FluoSim\CellSamples” directory the image called “*GFP-Nrx1b-Nlg1mcherry-merge.bmp*” representing two COS cells, one expressing GFP-Nrx1β (green), the other expressing Nlg1-mCherry (red), making an adhesive contact together (yellow).

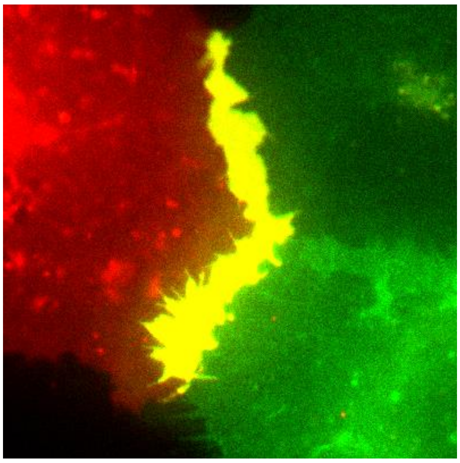

Figure 9 Adhesive contact between COS cells expressing GFP-Nrx1β and Nlg1-mCherry.

Alternatively, you might want to create your own cellular system so as to compare simulations to real experiments. In that case, open an image of your own (see compatible formats in the File Formats Section 3.1) from a different folder using the Browser. The selected image will appear on the screen. You can use the mouse pointer to position the cell on the screen and the mouse scrolling wheel to

zoom in and out. You can also adjust the *Image intensity* using the dedicated slider (amplification varies from 0 to 10). It is possible to show or hide the background image by checking the display image checkbox. Finally, the background image can be smoothed using bilinear interpolation by checking the Smooth image checkbox.

4.3 Importing existing geometries

Geometries can be directly imported from geometry files. FluoSim can deal with two file formats: Metamorph geometry file format **(.rgn)** and ImageJ geometry file format **(.roi)** (see section 3.1 File format for more information).

To import an already saved geometry, just click on the **Import geometries** button on the geometry tabs (see Figure 7). This action will open a window which allows you to select the file containing the geometry you want to import. Any newly imported region will be added to the existing regions and become visible in the region tabs. By default, these new regions will be measurement regions and hence will not influence the dynamics of the molecules. For example, open the pre-saved *COS Nrx-Nlg contact.rg*n file contained in the sample folder, which will highlight the cell contour (Region 0), the cell-cell contact (Region 1, Trapped), and the free region (Region 2, not Trapped).

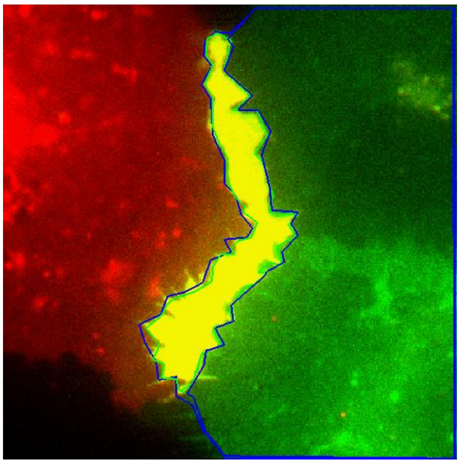

Figure 10 **Adhesive contact image and regions**

Remark: A Metamorph geometry file (.rgn) can contain more than one region contrary to an ImageJ geometry file (.roi) which has only one region per file.

4.4 Drawing a new geometry

Alternatively, you can draw ROIs using the *ToolBox* on the top menu. This Toolbox allows you to draw 3 types of geometries: **rectangle**, **circle** and **polygon**. You can start to draw a geometry by left clicking on the corresponding icon.

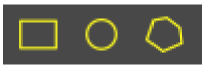

Figure 11 **Geometry Toolbox**

To draw a **rectangle**, left click to set a first corner on the screen, then move the cursor while maintaining the mouse button pressed, the cursor position defines the opposite corner position and hence the rectangle geometry. Release the mouse button to set the geometry.

To draw a **circle**, left click to set the center on the screen and move your cursor while maintaining the mouse button pressed to change its radius. The geometry is set by releasing the mouse button.

While drawing a **polygon**, left-click to define the first vertex on the screen, the next left clicks will set the other corners of the polygon, to conclude the drawing click around the first vertex to loop the geometry. During the drawing you can delete the last vertex that has been drawn with a right click.

Remark: since the working space is not discretized in FluoSim, it can be tedious to draw geometries whose vertices perfectly overlap. If you encounter such difficulties and you need overlapping vertices, you should consider drawing these regions in another imaging software such as Metamorph of ImageJ that work with pixel-based images and then import them in FluoSim (see section 4.3 Importing existing geometries for more information).

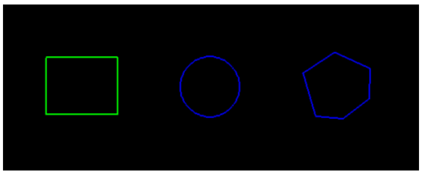

Figure 12 **Geometry Types**

4.5 Managing geometries

If selected in the region table, a region can be deleted by clicking on the delete region button (see Figure 7). The reset geometry button is used to delete the whole set of regions. Once the geometries have been created, you can export them in a file using the export geometries button. A browser window is then opened to let you choose the destination folder, the file name and the file format being used. Two formats are accessible: Metamorph File Format (.rgn) and Scalable Vector Graphics (.svg) format.

4.6 Simulation Length Scale

The **Simulation Length Scale** is of utmost importance for calibrating distances. It is defined through the pixel size parameter and corresponds to the pixel size (expressed in µm) of the reference image being used to create the geometry.

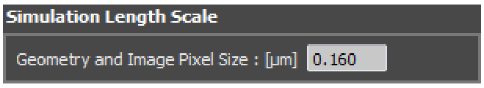

Figure 13 **Simulation Length Scale**

Remark: the pixel size of an image depends on the pixel size and the binning mode of the camera used to acquire the image, as well as on the magnification of the microscope objective. For example, for an image acquired with a 100x objective and a camera pixel size of 16 µm without any binning (binning = 1), the image pixel size is 0.16 µm.

PITFALL: Any mistake made on the pixel size parameter will have dramatic consequences on the simulated trajectories since the length scale will be wrong.

Remark: All the geometric objects of FluoSim (images and regions) are expressed in pixels, but physical parameters are defined using micrometers, i.e. diffusion coefficients are expressed in µm²/s and not in px²/s. Then two length units coexist in FluoSim: pixels and micrometers. This is the role of the Simulation Length Scale to provide a way to convert one unit into the other.

5. Entering molecules

5.1 Setting the number of molecules

You can set the number of molecules in the cell by moving the slider Copy number (from 0-150,000). This will populate the entire cell area. Molecules are initially created at random locations within the cell contour. You can always reset the molecular system by pulling the slider Copy number to zero, then adding molecules again.

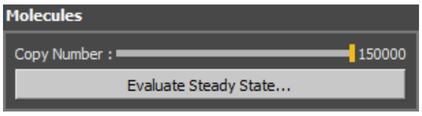

Figure 14 **Particle System Interface**

5.2 Reaching steady state

If kinetic properties are not uniform in the virtual cell (e.g. existence of compartments with different diffusion coefficient, crossing probability ≠ 1, or trapping), this initial random state will not correspond to the dynamic equilibrium of the system (i.e. as many molecules are entering and leaving the trapped areas per unit of time). To reach the binding steady state, you can perform a long-enough simulation and wait until the density of molecules in trapping areas stays constant over time, by checking the intensity using the plotting system (but it is difficult to estimate *a priori* the time to reach this equilibrium).

For convenience, we have implemented in FluoSim a way to evaluate the steady state by offering a possibility to place the molecules non-uniformly. To this aim, the algorithm gives prevalence for regions exhibiting trapping and/or low diffusion coefficient (parameters which contribute to retain molecules in a compartment). You can use this method by clicking on the **Evaluate steady state** box, this will let the program place molecules automatically. You may notice that the crossing probability also plays a role, i.e. regions with low crossing probability will be populated with lower molecular density than the rest of the cell. For more details on the calculation of the steady-state density distribution, see the Online Methods.

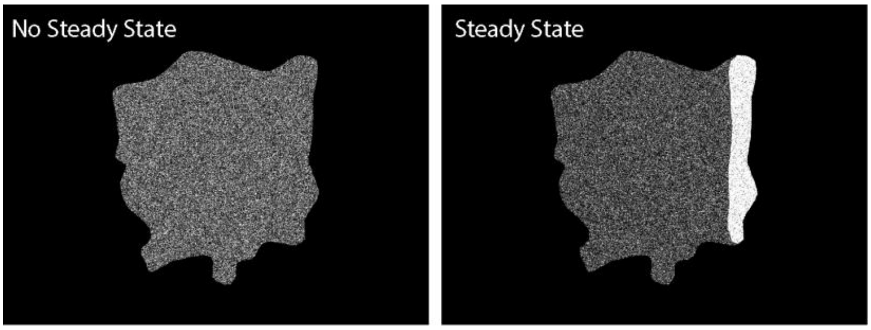

Figure 15 **Reaching Steady State**

PITFALL: For very low porosity parameters (crossing probability < 0.2), this calculation does not fully agree with the actual steady state being reached by the system. Hence one might want to perform an additional pre-sequence to reach the real equilibrium.

Remark: Choosing binding and unbinding rates both equal to 0 leads to an indeterminate form in the evaluation of the steady state distribution of molecules. In that case, we made the arbitrary choice to populate the system with as many trapped molecules as freely diffusing molecules in all trapping regions.

6. Image rendering

The rendering of the simulated images can be modified by several options accessible in the Rendering Parameters panel presented below:

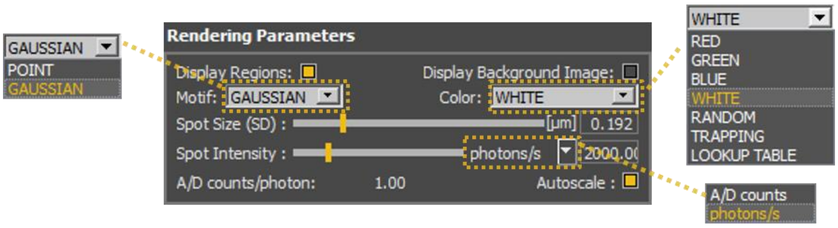

Figure 16 Rendering Parameters Interface

6.1 Geometry and image rendering

For better visualization of the simulation you can show or hide the reference image and the ROIs by checking or unchecking respectively the "Display regions" or the "Display Background Image" checkboxes.

6.2 Particle rendering

6.2.1 Particle motif

The motif of the molecules can be modified with the Motif combo box. FluoSim allows you to choose between two representations: **Point** and **Gaussian**. The **Point** representation is not affected by the zoom level: particles are always displayed on the screen as single pixels. This representation is the most accurate and allows you to observe what happens at the molecular level.

Alternatively, in the **Gaussian** representation, a point spread function (PSF) is used to mimic diffraction through the optical microscope, which depends on the objective numerical aperture and wavelength of fluorescence emission. In FluoSim, the PSF is approximated by a Gaussian intensity profile with a standard deviation (SD), σ in the order of λ/(2 x N.A.), where λ is the emission wavelength of the fluorophore, and N.A. is the numerical aperture of the objective. σ is entered in the interface as the Spot Size parameter. The full width at half maximum (FWHM) of the intensity profile is then equal to 2σ √(2.ln2). Using this kind of representation fluorescence-like images can be generated.

In the Gaussian representation, the Spot Intensity parameter gives the peak value of the Gaussian intensity profile of a single molecule. Depending on the unit selected in the associated combo box ([A/D Counts] or [Photons/s]), it represents either the peak intensity as read on the virtual camera coded on a 16-bit scale (0-65535 grey levels), or the peak emission rate of the fluorophore expressed in photons per second. When the Spot Intensity is expressed in units of photons/s, one has to specify the conversion rate, i.e. the gain in [A/D Counts Per photon], which gives the number of grey levels that will be read on the virtual camera chip per incoming photon. This parameter models the camera measurement chain from photon collection (quantum efficiency) and amplification (EMCCD gain) to conversion (Analog Digital Convertor). This parameter is disabled when the Spot Intensity is expressed in A/D Counts, since it is no longer into account during fluorophore rendering. When the Spot Intensity unit is changed, the program modifies the Spot Intensity value so that the resulting intensity on the camera (in A/D Counts) remains the same (when the Spot Intensity value range associated with the new unit allows it).

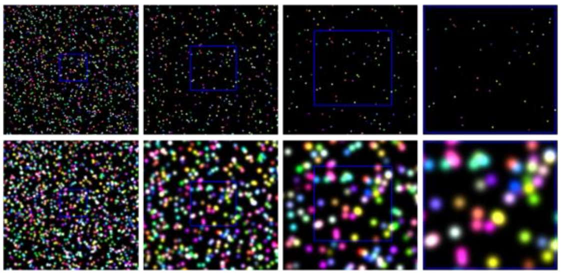

Figure 17 **Motif comparison: point vs Gaussian at different zoom levels**

6.2.2 Particle Color

The color associated with the molecules can be accessed with the Color combo box. It lets you choose between several colors (white, red, green and blue) or 3 rendering modes (random color, trapped-state color and look-up table mode).

Random color: each molecule has a unique color which is kept during the simulation.

Trapped state color: the molecule color depends on its trapping state: molecules trapped in a membrane compartment appear in red, while freely moving molecules appear in green.

Look-up table mode: is only available in the Gaussian representation, the particle color represents the intensity of the fluorescence emission, i.e. hot colors (orange-red) represent high intensity, while cold colors (violet-blue) represent low intensity. Thus, upon accumulation of molecules in a trapping area, the ROI will progressively become redder compared to ROIs where molecules diffuse freely.

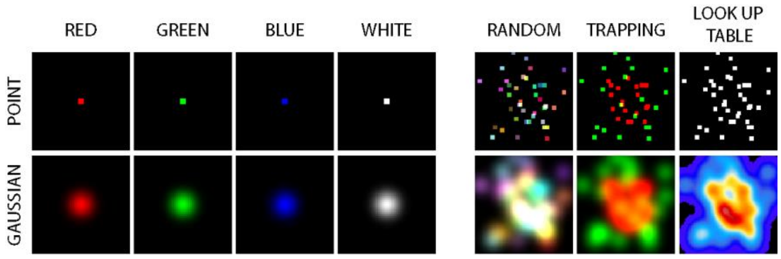

Figure 18 **Particle Color and representation in point vs Gaussian rendering modes**

7. Kinetic parameters

7.1 Diffusion-trapping model

In FluoSim, the cell membrane is modelled with a 2D Euclidean Surface on which molecules can diffuse (with coefficient D_{out}). These molecules can interact with an infinite pool of binding partners in specific areas named membrane compartments. In these areas, even before any reaction occurs, the mobility of the molecules might be reduced (D_{in}). Due to steric hindrance, molecules may have difficulty to enter these areas and hence may bounce on their boundary (Bouncing Probability). Molecules inside membrane compartments can react and form complexes which may diffuse more slowly ($D_{trapped}$).

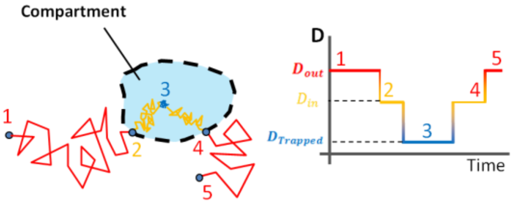

Figure 19 **FluoSim trapping model**

Below is the list of the kinetic parameters used in FluoSim:

Diffusion Outside (D_{out}): diffusion coefficient of molecules situated within the cell contour but outside of any membrane compartment.
Diffusion Inside (D_{in}): diffusion coefficient of molecules inside membrane compartment in the free diffusing state (i.e. not trapped). In the FluoSim interface, this parameter is either expressed as a percentage of D_{out} or in µm²/s depending on the unit selected on the combo box.
Diffusion Trapped (D_{trap}): diffusion coefficient of molecules inside a membrane compartment in the trapped state.
Binding rate (k_{on}): for a molecule inside a membrane compartment, this is the transition rate between the free-state and the trapped-state. Formally, k_{on} is defined by the relation: $k_{on}\delta t = P(trapped, t + \delta t|\overline{trapped}, t)$ and is expressed in sec^{-1}.
Unbinding rate (k_{off}): is the transition rate between the trapped-state and the free-state. It is expressed in sec^{-1}. Formally, k_{off} is defined by the relation : $k_{off}\delta t = P(\overline{trapped}, t + \delta t|trapped, t)$.
Crossing Probability. We first define a **Bouncing Probability** which is the probability that a free diffusing molecule bounces on the membrane compartment boundary when its displacement vector intersects the region geometry. When bounced, the molecule displacement vector is reflected, this new displacement vector is then used to compute the next position. The **Crossing Probability** is simply equal to 1 - Bouncing Probability: this is the probability that a freely diffusing molecule whose displacement vector crosses a membrane compartment boundary is not reflected by the boundary.
Immobile Fraction: gives the percentage of molecules which are immobilized. It may correspond to non-specific detections or internalized molecules. These molecules should not be mistaken with trapped molecules which can exchange between diffusive and trapped states contrary to immobilized molecules. Hence, the only way to release an immobilized molecule is by reducing the immobile fraction.
Fixation: To mimic sample fixation (e.g. by aldehydes), a treatment which immobilizes molecules in the cell, one can press on the Fixation checkbox: this will set all the diffusion coefficients to zero, and keep the binding/unbinding rates as defined previously. This is particularly relevant for the SRI mode which is generally preformed using fixed cells. By calculating only the fluorophore photophysics, and not the molecule dynamics, this Fixation function greatly accelerates the SRI performance.

7.2 Kinetic interface

Kinetic parameters are modified using the Kinetic Parameters interface accessible in the Live Experiments Tab and shown below.

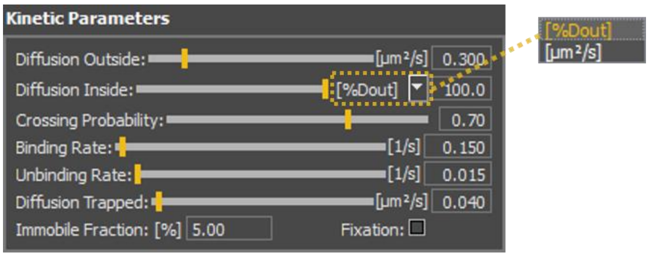

Figure 20 Kinetic Parameters Interface

8. Simulation progress

Because of the intrinsic nature of Brownian motion, the position of molecules cannot be calculated at every instant. We use the solution of the diffusion equation to make discrete jumps in time and compute the new positions and intensities of all molecules at each time step. The length scale of the x and y displacements is $\sqrt{2D\Delta t}$, which is multiplied by random numbers chosen in a normal distribution (see Online Methods).

8.1 Simulation progress interface

You can start, pause and stop the simulation progress at any moment by clicking respectively on the play, pause and stop icons. Stopping the simulation will reinitialize all the measurement probes, resulting in the clearing of the graph and the recorded data. You can set at any time the total number of steps to be simulated by changing the value in the step counter box. If you feel that the simulation is finishing too fast, just increase the total number of steps of the simulation by adding one or two zeros. Once started, you can follow the duration of the simulation with the progress bar and the step counter.

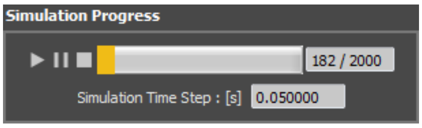

Figure 21 Simulation Progress Interface

8.2 Simulation Time Step

The **simulation time step** parameter (Δt) represents the duration of a jump: this is the time separating two calculated states in the simulator. The default Time Step is 20 ms (0.020 s) which is taken from a typical camera frame rate for SPT experiments. You can increase the time increment a bit (say to 50 ms or 0.1 s) if you want the simulation to run faster (e.g. for FRAP or PAF experiments). For FCS experiments, the time increment is usually reduced to values on the order of 1 ms, to be able to resolve the characteristic time for fast diffusing molecules to cross a small ROI corresponding to the size of a laser spot (typically 0.3 µm).

PITFALL: The distance travelled by a molecule within a time increment Δt has to remain small compared to the dimensions of the cell and its ROIs, e.g. for a diffusion coefficient D = 0.5 µm²/s and a simulation time step Δt = 0.1 s, the characteristic travelled distance is r = $\sqrt{2D\Delta t}$ ~ 0.3 µm.

Remark: the **simulation time step** should not be mistaken with the **computing time step,** which is the average duration it takes for the processor and the GPU to compute one jump and display the image on the monitor. The computing time step is not a fixed value and depends on several parameters, i.e. the number of molecules, the crossing probability, the rendering mode (longer for Gaussian than for point representation), and increases with the diffusion coefficient and simulation time step since the number of crossings and reflections increases with the distance travelled by molecules. The inverse of the computing time step is the number of simulated **frames per second (FPS)** shown in the upper left corner of the display window.

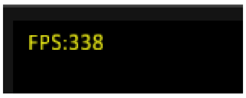

Figure 22 Simulation Frame Rate

9. Photophysics

9.1 Photophysics model

Photophysics of the fluorophores bound to molecules is taken into account through two photo-switching rates dictating the transitions between emitting and non-emitting fluorescence states. These rates are called respectively Switch-on rate (k_{on}^{Fluo}) and Switch-off rate (k_{off}^{Fluo}), and are expressed in s^{-1}.

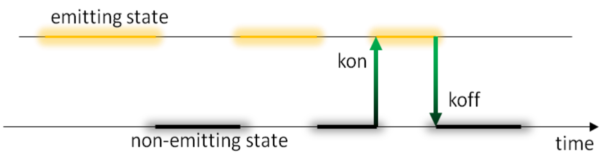

Figure 23 **Photophysics model**

For a given simulation time step Δt, the product k_{on}^{Fluo}.Δt is equal to the probability to transit from the non-emitting state to the emitting state. The inverse: $1/k_{on}^{Fluo}$ is equal to the average time the fluorophores spend in the non-emitting state. Similarly, the product k_{off}^{Fluo}.Δt is equal to the probability to transit from the emitting state to the non-emitting state. The inverse $1/k_{off}^{Fluo}$ is equal to the average time the fluorophores spend in the emitting state. You can change these rates between the values 0.001 and 10 s^{-1} using the two sliders: photophysics **switch-on rate** and **switch-off rate**. If the switch-on rate is zero, photobleaching is irreversible, while if the switch-on rate is non-zero, fluorophores can come back to an emitting state after having switched off (reversible photo-blinking).

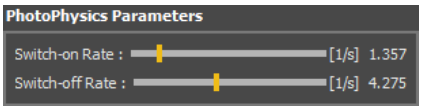

Figure 24 **Photophysics Parameter Interface**

9.2 Photophysics applications

You can simulate a simple photobleaching situation by increasing the bleaching rate while keeping the switch-on rate to zero, with or without molecule diffusion. Inversely, once all molecules are in the dark state, you can simulate a photoactivation by increasing the switch-on rate, while keeping the switch-off rate to zero. Now, if both rates have non-zero values, you will observe back and forth photo-conversion of fluorophores, such as in **Single Particle Tracking PhotoActivation Localization Microscopy (Spt-PALM)** experiments. The switch-on rate can be seen as being proportional to the 405 nm laser used to photoactivate PA-GFP or mEOS2 molecules, for example, while the switch-off rate can be seen as being proportional to the excitation laser power (i.e. 488 nm for PA-GFP, 561 nm for mEOS2). Usually, the switch-on rate is fairly low, to activate only a subset of molecules, while the switch-off rate is high, since strong laser power is commonly used to get the highest possible signal-to-noise ratio at maximal frame rate. Once the two parameters are set, a regime of steady-state fluorophore density is reached.

10. Plotting system

FluoSim has its own plotting system, which allows you to follow the evolution of critical properties of the system while the simulation is running. To display the FluoSim plot, click on the Show/Hide Plot action in the Window menu accessible from the FluoSim menu bar.

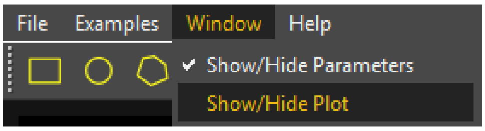

Figure 25 **Display and hide FluoSim plot**

Two axes of a graph should appear (intensity is in arbitrary units versus time in seconds). The graph is active only when a simulation is started. When you stop a simulation, the time goes back to zero and the graph is reinitialized. When you press the pause button, the graph stays still. You can choose the measured region at the bottom of the plot window. Choose Region 0, which represents the whole cell. Play a simulation, the graph plots the number of molecules in the whole system, divided by the area of the cell. Move the slider Copy number, you should see the step-by-step changes in the intensity on the graph. Now, choose region 1 or 2, corresponding to smaller sub-cellular compartments defined as trapping areas. Play a simulation. The intensity now represents the number of molecules in this ROI divided by the area of the ROI. Temporal fluctuations represent the fluxes of molecules entering and leaving the ROI over time. If you decrease the number of molecules to very low values (Copy number in the range of 100-1000 depending on the region size), you should see quantal steps revealing single molecules entering and leaving the ROI.

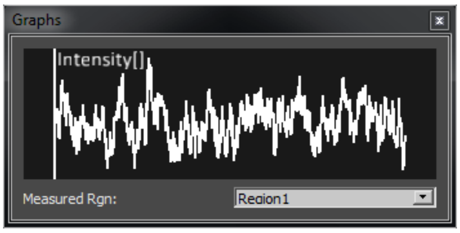

Figure 26 **Plotting Window**

11. Live Experiments

11.1 FRAP

To mimic **Fluorescence Recovery After Photobleaching** in real time, select the Photobleaching **Illumination type** under the menu Live measurements. Enter a large number of molecules to reach an almost full coverage of the cell surface (100,000-150,000). Unless you are studying very fast diffusing processes where you need good temporal resolution, you can in general increase the time step to 0.1-0.2 sec, so as to gain time in the simulations. Also select the ROIs to be photobleached (Photobleached Rgn). You have to select the same ROI in the graph to be able to follow the fluorescence recovery. Open the graph window and run the simulation. Once the intensity in the ROI has reached a steady state, click on the button Start Photobleaching.

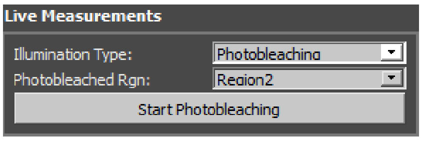

Figure 27 **Live Experiment: Photobleaching Interface**

The intensity goes to zero in one frame, and gradually recovers. The number of unbleached molecules repopulating the area through a combination of diffusion and trapping is plotted over time. You can play on the diffusion coefficients, binding rates, and immobile fraction, to see how the curve is affected.

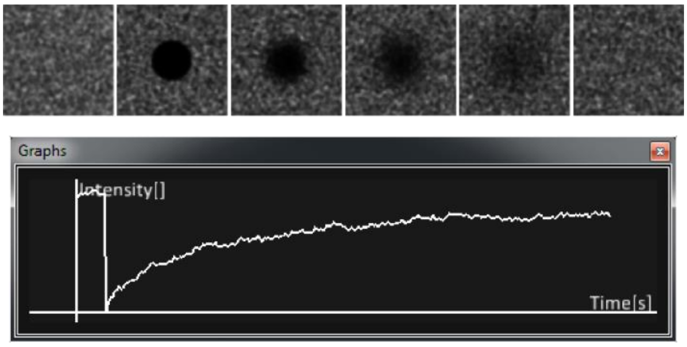

Figure 28 **Simulated FRAP Experiment**

11.2 PAF

PhotoActivation of Fluorescence (PAF), which is basically the mirror of a FRAP experiment, can also be mimicked. Initially no molecules are fluorescent, until one briefly activates them in a specific compartment. Thus, place all molecules in the dark state by increasing the switch-off rate to its maximal value (10 sec^{-1}), while keeping the switch-on rate to zero. When this is done, stop the simulation, and put the switch-off rate back to zero (while the switch-on rate stays at zero). Under the window Live measurements, select the Photoactivation **Illumination type**, and select the ROI where photoactivation will be performed.

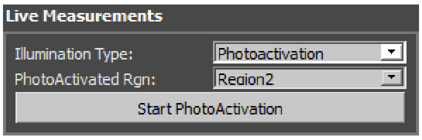

Figure 29 **Live Experiment: Photoactivation Interface**

Open the graph window, select the region to be photo-activated, play the simulation, and click on the button Start PhotoActivation. The intensity goes to maximum in one frame as all the fluorophores are turned on, then gradually decreases, as molecules detach and diffuse out of the ROI.

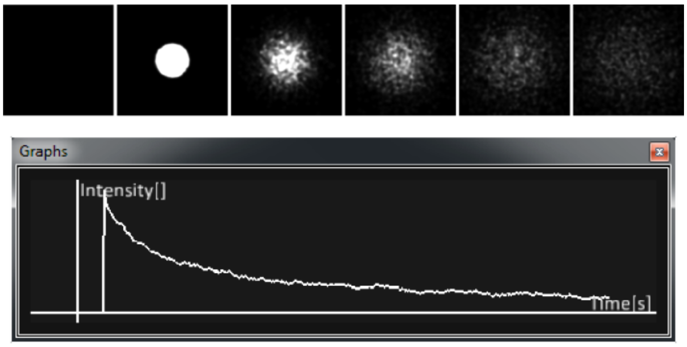

Figure 30 **Simulated PAF Experiment**

12. Recorded experiments

Once all the parameters have been adjusted in the LIVE Mode and yield realistic results, you can use the EXPERIMENT Mode to perform virtual experiments and save the simulated data and compare them to actual experiments. The initial state of the system at the beginning of the recorded experiment is the exact same state as the one you have left in the LIVE mode. Indeed, the positions of the molecules, their trapping and fluorescence states are conserved in the EXPERIMENT mode and so are the simulation parameters like the pixel size, the simulation time step and the kinetic parameters.

Several parameters need to be specified before being able to perform a recorded experiment. Some parameters, as the experiment duration or the number of repetitions, are common to all the experiments while others parameters are specific to a given experiment. You can find below a list and a description of these parameters:

12.1 General experimental parameters

Below is given the interface used to modify the General Parameters of the simulations in the EXPERIMENT mode:

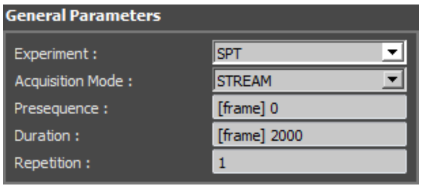

Figure 31 **General parameters**

Experiment Type

You can choose between 6 types of experiments: SPT, FRAP, PAF, FCS, SRI and DRUG. The combo-box shown below is used to modify the type of experiment being performed:

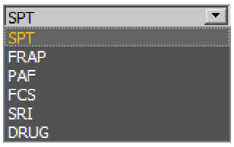

Figure 32 **Experiment Types**

Acquisition mode

Two acquisition modes are accessible: Stream and Timelapse modes. In the Stream mode, the recording sampling period is equal to the simulation time step, i.e. the algorithm performs a recording at every single time step. As an example, if you perform a SPT experiment with a duration of 2000 frames in Stream Mode, 2000 successive displacements will be computed and all the molecule positions will be recorded during these 2000 frames. As a result, if no photobleaching was set (i.e. k_{off}^{Fluo} =0), the recorded trajectories will be 2000 frames long, while if there is photobleaching (i.e. k_{off}^{Fluo} > 0), trajectories will be shorter. The Stream mode is well suited for SPT and FCS experiments since one needs to capture the rapid fluctuations of the molecule positions and intensities. SRI also needs to be performed in stream mode, to record all molecule localizations.

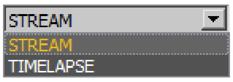

Figure 33 **Acquisition Modes**

In the Timelapse mode, computations are still performed at every single time step, but recordings are performed at a lower frequency. Hence, the recording sampling period has to be a multiple of the simulation time step. This sampling mode may be interesting to use in the FRAP and PAF experiments, where dynamics is usually slow. In the Timelapse mode, The Sampling Period parameter is accessible and can be modified in the interface.

Experiment duration and pre-sequence

A recorded experiment consists of two phases: a pre-sequence and a recording phase. The pre-sequence is used to reach the steady state: during this phase, the simulator updates the positions and states of the molecules but does not perform any recording.

Presequence (N_{pres}): corresponds to the number of frames being simulated before the recordings actually start.

Remark: if you consider that your system is already at equilibrium, e.g. if you previously clicked on the Evaluate Steady state button, you can skip this phase by setting the pre-sequence value to 0.

Duration (N): this is the number of frames during which the simulation and the recording are performed.

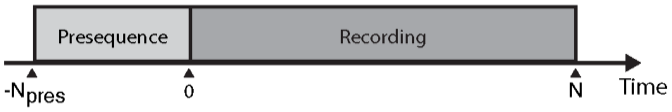

Figure 34 **Experiment Timeline**

Repetitions

Due to the intrinsic stochasticity of the simulated processes, the exported results might slightly differ from one simulation to the next. The variability originates from the initial state of the simulation (positions, trapping and fluorescence states) and from the randomness of the Brownian motion and the state transitions. Hence, FluoSim gives you the possibility to repeat several times the same experiment. The number of repetitions being simulated is entered as a repetition parameter.

Pitfall: although the first repetition starts with the same state as the one left in Live Mode, the next repetitions begin with a state computed using the Evaluate Steady State method. This is because the simulation state at the end of a repetition might not be the steady state and hence cannot be used as an initial condition for the next repetition.

12.2 Simulation outputs

Destination Folder

You can select the destination folder in which the simulated data files will be saved by clicking on the Browse button. By default, the simulations will be exported in the folder called “Simulations output”.

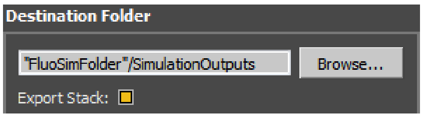

Figure 35 **Destination Folder**

Simulation Stack Export

For all the imaging modes described below i.e. SPT, FRAP, PAF, FCS, and DRUG, but not for SRI which is specific, one can export a movie of the simulation as a multipage tiff but only if a background image has been loaded and the export stack box has been clicked. The background image is used to describe the field of view from the virtual camera chip, and hence, all the images exported in the stack have the same definition and position as the background image, making it compulsory. At the end of the simulation, a multi-TIFF stack of images entitled “Stack.tiff” is created in the chosen Destination folder (by default Simulation Outputs).

In this mode, the rendering pipeline is modified to produce realistic fluorescence images using several sources of noise which are given below. Note that photon related offset and noise are only available when the Spot Intensity is expressed in Photons/s.

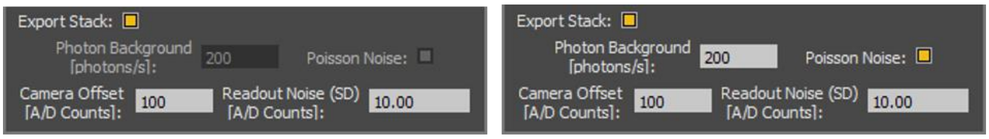

Figure 36 Simulation Stack Export Interface, Spot Intensity in A/D counts per sec (left) and in photons/s (right)

Only available when Spot Intensity’s unit is in Photon/s:

Photon Background [photon/s]: corresponds to background photons which could originate from auto-fluorescent molecules or parasite light. This photon background level is set uniformly on the virtual camera sensor and is added to the incoming photons collected on each pixel. The Photon Background level is expressed in photon per sec (per pixel), hence the number of background photons collected on a single pixel during one frame is equal to the Photon Background times the Simulation Time Step (Δt).

Poisson Noise: models the stochastic emission of photons and can be either enabled or disabled depending on the associated check box state. When enabled, once the fluorescent molecules have been rendered and the Photon Background level has been added, hence resulting on an image containing the mean number of photons associated with the overlapping Gaussian profiles and the background, FluoSim generates for each pixel a random number following a Poisson distribution of mean (and variance) equal to the original pixel value (i.e. the mean photon number collected by the pixel), thereby producing a new intermediate noisy image.

(in Live Panel) Gain [A/D Counts Per Photon]: is used to transform the image in photons (possibly noised with Poisson) into an image in A/D Counts: a 16-bit image similar to what would be obtained on a fluorescence microscope camera.

Always available:

Camera Offset [A/D Counts]: gives the number of A/D Counts added to each pixel after the conversion from photons to A/D counts has been made, and before the pixel readout.

Readout Noise (SD) [A/D Counts]: models the uncertainty introduced during the readout of a single pixel value. This noise is implemented as a Gaussian distribution and is defined by its standard deviation expressed in A/D counts. Hence, each pixel value of the image is summed up with a different random number which follows a normal distribution of 0 mean and of standard deviation SD as defined via the interface.

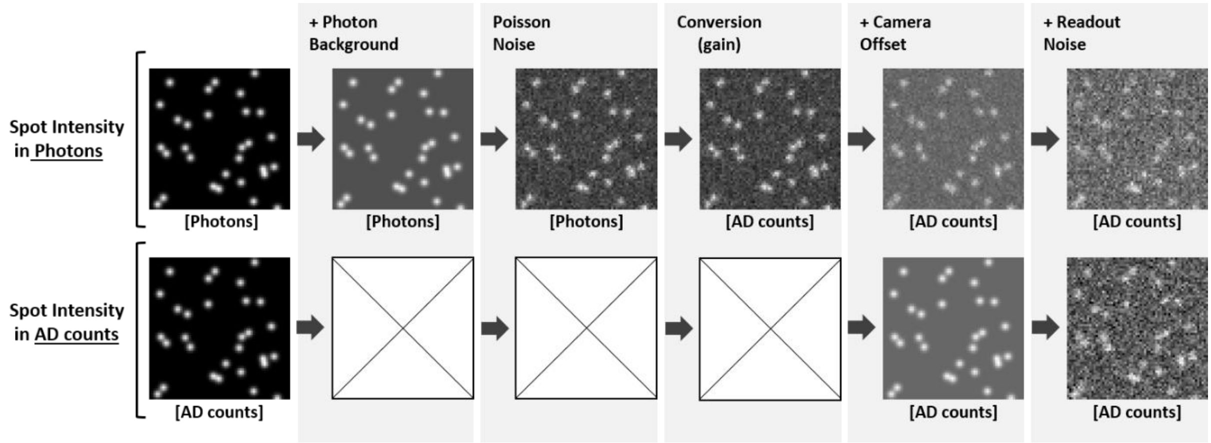

Figure 37 Rendering Pipeline with offsets and noise sources

We give below the formulae giving the value (in A/D Counts) of the pixel (i,j) of an image of the exported stack:

If Spot Intensity is in Photon/s:

$$\mathrm{pixel(i,j)} = \mathrm{ADCounts\_PerPhoton} * \mathrm{Poisson(Signal(i,j)} + \mathrm{PhotonBackground)} + \mathrm{CameraOffset} + \mathrm{Normal(0, ReadoutNoise)}$$

where Signal(i,j) corresponds to the total number of photons emitted by the fluorophores and collected by the pixel at coordinate (i,j).

If Spot Intensity is in A/D Counts:

$$\mathrm{pixel(i,j)} = \mathrm{Signal(i,j)} + \mathrm{CameraOffset} + \mathrm{Normal(0, ReadoutNoise)}$$

where Signal(i,j) corresponds to the total intensity in A/D Counts associated with the emitted photons by the fluorophores and collected by the pixel at coordinate (i,j).

Simulation Outputs Names

The name and nature of the exported files depend on the experiment type being simulated. A rapid description of these files is given for each experiment below.

12.3 SPT experiments

Principle

During a SPT experiment, films displaying the movement of sparse fluorescent molecules are recorded. Thanks to their low spatial density, molecules can be localized and their trajectories can be reconstructed. These trajectories can then be used to investigate the dynamics of the molecules. To perform the analysis, different strategies can be employed like calculating the Mean Square Displacement (MSD) and fitting it with a linear function of time, as done here.

FluoSim SPT experiments do not generate raw images but directly provide the trajectories of the fluorescent molecules which are tracked during the simulation. The molecules being localized (and hence tracked) are those inside a **recorded region** and in the fluorescent state.

Specific Parameters

Recorded Regions are the regions in which the trajectories will be recorded. The list of recorded regions is visible in the Recorded Region tabs. To add a region to the list, select a region in the comboBox and then click on the "Add Region…" button next to it. The region now appears in the recorded region list. To remove a region from the list, select its name in the list and click on the "Remove Region…" button.

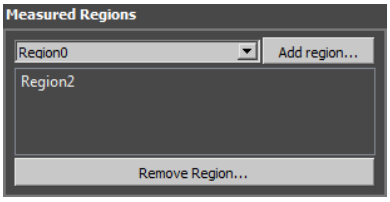

Figure 38 **Recorded Regions**

Outputs

At the end of an SPT simulation, the simulator exports one file per recorded region and per repetition in the destination folder (Simulation outputs). These files contain the trajectories of the fluorescent molecules being tracked during the simulation. Their name starts with "trace " followed by "_rgn" and the index of the recorded region followed by "_rep" and the index of the repetition. For example the file "trace_rgn0_rep0.txt" corresponds to the recorded trajectories inside the region 0 during the first repetition.

12.4 FRAP experiments

Principle

FRAP is a fluorescence-based ensemble technique used to assess the dynamic properties of molecules. This technique works by altering the fluorescence in specific regions of the cell using photobleaching and by measuring the recovery of fluorescence intensity in the photobleached regions as a function of time, which depends on the rate at which adjacent fluorescent molecules repopulate the photobleached regions. These recordings usually display three phases: an initial baseline which corresponds to a steady fluorescence in the regions, a sharp loss of fluorescence during the photobleaching process, and a progressive recovery to the fluorescence steady state.

To reproduce this situation in FluoSim, after having simulated the pre-sequence, the simulator starts to record a baseline in the **Recorded Regions**, then performs the photobleaching of the **Bleached Region** during several time steps (**Bleaching duration**) at a given **Bleaching Rate**. Finally the simulator continues to record the fluorescence recovery until the end of the simulation.

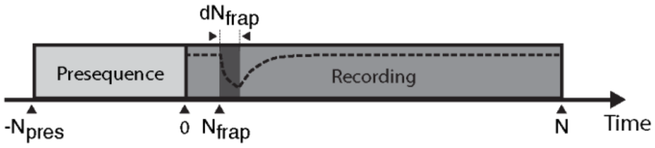

Figure 39 **FRAP Experiment Timeline**

Parameters

Bleaching Starting frame (N_{frap}): corresponds to the index of the first frame being photobleached.

Bleaching Duration (dN_{frap}): gives the number of frame being photobleached.

Bleaching Rate (k_{off}^{bleach}): corresponds to the rate at which molecules inside photobleached regions get bleached by the laser. This is the probability that a non-photobleached molecule gets bleached per unit of time i.e. $k_{off}^{bleach}.\delta t = P(\text{bleached}, t + \delta t | \overline{\text{bleached}}, t)$.

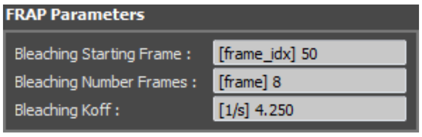

Figure 40 **FRAP Parameters**

Recorded Regions: are the regions in which the fluorescence intensity will be measured. The list of recorded regions is visible in the Recorded Region tabs. To add a region to the list, select a region in the comboBox and then click on the "Add Region…" button next to it. The region now appears in the recorded region list. To remove a region from the list, select its name and click on the "Remove Region…" button.

Bleached Regions : are the regions in which molecules get photobleached. Similarly to what has been described for the Recorded Region tabs you can add, delete and visualize the bleached regions in the Bleached region tab.

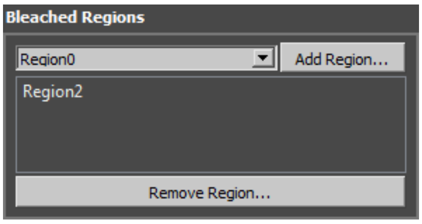

Figure 4136 **Bleached Regions**

Outputs

At the end of the simulations, output files are saved in the destination folder. There is one text file per region and per repetition. Their name starts by "averageIntensity" followed by "_rgn" and the index of the recorded region followed by "_rep" and the index of the repetition. For example the file "averageIntensity_rgn0_rep0.txt" corresponds to the recorded intensity inside region 0 during the first repetition.

Important remark: To significantly accelerate the FRAP, PAF, and FCS simulations, especially if your computer is not equipped with a performant graphics card, you can choose to image molecules using the point representation (do this in LIVE mode, then go back to the EXPERIMENT mode). The program will gain a lot of time by not displaying Gaussian profiles on the screen, while all the position intensity calculations remain the same.

12.5 PAF experiments

Principle

As with FRAP, PAF experiments are used to assess the dynamics of a large number of molecules. This technique relies on the photoactivation of molecules contained in a given region of interest. Due to their mobility, molecules leave the region; by recording the decay in fluorescence intensity as a function of time, one can measure the rate at which molecules escape the region. These recordings usually display three phases: an initial plateau which corresponds to the absence of fluorescence, a sharp increase of fluorescence during the photoactivation process, and a progressive loss of fluorescence as the molecules leave the region.

To reproduce this situation in FluoSim, after having simulated the pre-sequence, the simulator starts to record a baseline in the **Recorded Regions**, then performs the photoactivation in the

Photoactivated Regions during several time steps (**Activation duration**) at a given **Activation Rate**. Finally, the simulator continues to record until the end of the simulation. Below is the PAF Experiment timeline:

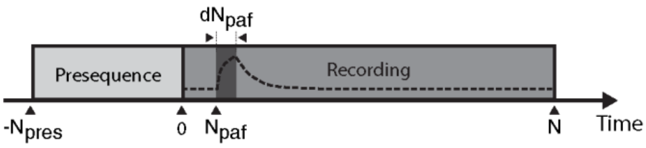

Figure 42 **PAF Experiment Timeline**

Parameters

Activation starting frame (N_{paf}): corresponds to the index of the first frame being photoactivated.

Activation duration (dN_{paf}): gives the number of frames being photoactivated.

Activation Rate ($\mathbf{k_{on}^{activation}}$) : corresponds to the switch-on rate at which molecules inside photoactivated regions get activated. This is the probability that a non-fluorescent molecule gets photoactivated per unit of time i.e. $k_{on}^{activation}.\delta t = P(activated, t + \delta t \mid \overline{activated}, t)$.

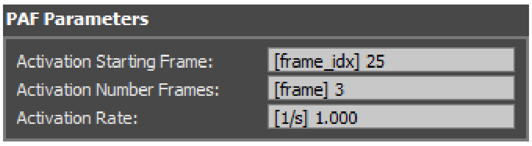

Figure 43 **PAF Parameters**

Recorded Regions: are the regions in which the fluorescence intensity will be measured. The list of recorded regions is visible in the Recorded Region tabs. To add a region to the list, select a region in the comboBox and then click on the "Add Region..." button next to it. The region now appears in the recorded region list. To remove a region from the list, select its name and click on the "Remove Region..." button.

Photoactivated Regions: are the regions in which molecules get photoactivated. Similarly to what was described for the Recorded Region tabs you can add, delete, and visualize the activated regions in the Photoactivated region tab.

Outputs

At the end of the simulations, output files are saved in the destination folder. There is one text file per region and per repetition. Their names start by "averageIntensity" followed by "_rgn" and the index of the recorded region followed by "_rep" and the index of the repetition. For example, the file "averageIntensity_rgn0_rep0.txt" corresponds to the recorded intensity inside the region 0 during the first repetition.

12.6 FCS experiments

Principle

In an FCS experiment, the fluctuation of fluorescence inside a small detection volume is recorded as a function of time. These rapid fluctuations originate from the movement of the molecules which can enter and leave the detection volume. The time span during which a molecule remains in the detection volume is related to its dynamic properties and can be obtained from the time-correlation of the fluctuations. Hence by studying the fluctuations one can infer on the dynamics of the studied molecules. Usually, the detection volume is a focused laser beam which can be approximated by a 3D Gaussian volume. When used to investigate the dynamics of membrane proteins, the detection volume

is a 2D Gaussian profile. In FluoSim, two modes of illumination are available to perform FCS: 1/ a uniform illumination mode in which all fluorescent molecules in the recorded region are detected with the same intensity; and 2/ a Gaussian illumination mode in which molecules are detected only in a 2D Gaussian beam mimicking a focused laser beam. In this mode, molecules are bleached proportionally to the local beam intensity so that the bleaching rate depends on the molecule position in the Gaussian profile, centered on the region centroid.

Simulation
In the LIVE mode, populate the cell with relatively few molecules (say 500-3000). In the menu EXPERIMENT, select the FCS experiment. Choose a small time increment (typically 1-5 ms), and a number of steps spanning several time decades (~100,000). Click on the stream acquisition mode. Under the window Measured Regions, enter the ROI where measurement will be performed.

Parameters
The **Enable Gaussian Beam** checkbox is used to switch between these two illumination modes. If no Gaussian beam is set, the intensity of the molecules is homogeneous in the recorded region and no photobleaching occurs. If a Gaussian beam is used, several parameters have to be defined:
Beam Sigma ($\boldsymbol{\sigma_{\mathrm{beam}}}$): corresponds to the standard deviation of the intensity distribution of the Gaussian beam.
Beam Max Intensity ($\mathbf{I_0}$): is the maximum intensity of the Gaussian beam at its center.
Beam Bleaching Rate ($\mathbf{k_0}$)*:* corresponds to the switch-off rate where the intensity is maximal (i.e. at the center of the beam). The beam Intensity distribution is given by the relation: $I(r) = I_0 e^{-\frac{r^2}{2\sigma^2}}$ and the switch-off rate distribution by: $k(\boldsymbol{r}) = k_0 e^{-\frac{\boldsymbol{r}^2}{2\sigma^2}}$, where **r** designates the molecule position vector from the center of the beam.

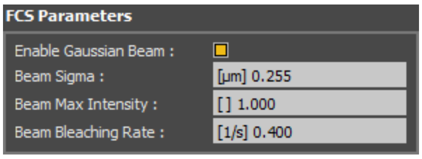

Figure 44 **FCS Parameters**

Outputs
Two files are exported in the destination folder per recorded region and per repetition: the intensity fluctuations as a function of time, and the computed auto-correlation function.
The name of the intensity file starts by "averageIntensity" followed by "_rgn" and the index of the recorded region followed by "_rep" and the index of the repetition.
The name of the auto-correlation file starts with "correlation" and is followed by "_rgn" and the index of the recorded region followed by "_rep" and the index of the repetition.

12.7 SRI experiments

Principle
Super Resolution Imaging (SRI) does not investigate the dynamics of molecules but aims at generating super-resolution images with finer details than classical imaging techniques limited by optical diffraction. Different strategies can be used to generate these images, in particular single molecule localization techniques. In dSTORM, the stochastic blinking of specific fluorophores such as Alexa dyes is used to obtain stacks of images capturing sparse fluorescent molecules. These image stacks are then

analyzed to retrieve the molecules localizations and create the super-resolution image with a smaller pixel size. To produce such images, different rendering methods can be used: localizations can be displayed as single pixels or as Gaussian intensity profiles with finer spatial distribution than the molecule diffraction spot. FluoSim uses a pixel-based rendering mode, hence each detection is associated with a single pixel.

Parameters

Intensity: associated with a single detection. The default value is 1, but one can increase it to 10 or 100 to increase the grey level scale on the displayed image.

Zoom: determines the size of the super-resolved image: it corresponds to the ratio between the pixel size of the reference low-resolution image and that of the super-resolved image (e.g. with a zoom of 5, a low resolution 0.16 µm pixel size becomes 32 nm in super-resolution).

Localization Precision: quantifies the inaccuracy of the localization process, and corresponds to the standard deviation of the Gaussian distribution of detections around the actual position of the molecule (e.g. σ = 25 nm, FWHM ≈ 60 nm).

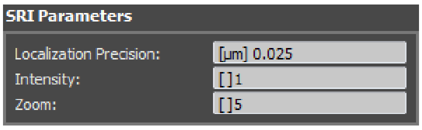

Figure 45 **SRI Parameters**

Output

At the end of the SRI simulation, a single 16-bit image is generated which contains the sum of all molecule localizations throughout time. FluoSim exports one TIFF image per repetition cycle in the destination folder (Simulation Outputs). The name of the image is SRI_screen_capture_rep0.tiff, where "rep" is the repetition number.

12.8 DRUG experiments

Principle

Independently of changes in the fluorescence parameters, FluoSim can also be used to predict changes in the sub-cellular distribution of membrane proteins over time, induced by the modification of diffusion coefficients (D_{out}, D_{in}, D_{trap}) or kinetic rates (k_{on}, k_{off}). This mode is intended to mimic for example the effect of crosslinking molecules like antibodies that decrease diffusion coefficients of membrane proteins, or the action of pharmacological agents, competing peptides, and chemogenetic or optogenetic stimulations that modulate specific protein-protein interaction rates. Such types of modifications in dynamic parameters during the course of a simulation have collectively been called DRUG. After you have defined your cell geometry, number of molecules, and basal dynamic parameters in the LIVE menu, choose the Experiment mode called DRUG in the General parameters of the EXPERIMENT menu. Choose the parameter you want to change (only one parameter is allowed at a time), its final value, and the time during the simulation at which you want the step change to be applied.

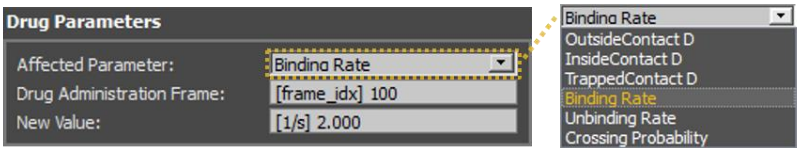

Figure 46 **Drug Experiment parameters**

Parameters

Affected Parameter: corresponds to the Dynamic Parameter affected by the drug. This value has to be chosen is the following list: {OutsideContact D, InsideContact D, TrappedContact D, Binding Rate, Unbinding Rate, Crossing Probability}.

Drug Administration Frame: gives the frame index during which the drug is administrated and hence the Affected Parameter is modified.

New Value: corresponds to the new value taken by the Affected Parameter after the drug has been administrated.

Output

At the end of the simulations, the output files are saved in the destination folder. There is one text file per region and per repetition. Their names start by "averageIntensity" followed by "_rgn" and the index of the recorded region followed by "_rep" and the index of the repetition. For example, the file "averageIntensity_rgn0_rep0.txt" corresponds to the recorded intensity inside the region 0 during the first repetition.

13. Analysis of SPT experiments

The simulator is intrinsically designed for single molecule tracking such as in SPT-PALM or uPAINT experiments. A dedicated post-processing module is thus provided to analyze in details the results of the SPT simulations (SPT ANALYSIS).

13.1 Importing trajectories

Load a previously recorded .trc file using the Browse button. (For more information on the .trc format see the paragraph 3.1.3 *Trajectory Formats*).

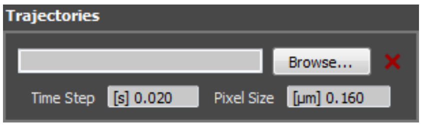

Figure 47 **Trajectory Loading Interface**

The localization events and reconnected trajectories appear on the screen, in an image stack which can be played back and forth using the orange slider at the bottom. In the bottom left of the image, the frame number is indicated, as well as the current number of trajectories and the number of localization events after filtering.

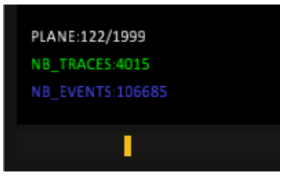

Figure 48 **Frame selection slider and trajectory file information**

Since the trajectory files data are expressed in pixels and frames, FluoSim needs to know the size of a pixel and the duration separating two consecutive frames to compute the MSDs and diffusion coefficients. To this purpose, two parameters are used: the Time Step duration (in sec) and the Pixel Size (in µm). These parameters are accessible via two fields in the interface.

13.2 Trajectory rendering

Several rendering modes are accessible, for both the molecules (**spot size and spot intensity**) and their trajectories (**line size and line intensity**), with different color codes (black and white, random, diffusion, instant diffusion, and length). Whereas in random mode, each trajectory and its fluorescent events are associated with a unique random color, in black-and-white mode they are rendered in white. In the diffusion and length color modes, **Look Up Tables (LUTs)** are used to represent the properties of the recorded trajectories. Several LUTs can be selected in the dedicated comboBox, which code for the trajectory properties. In this case, properties with values smaller than the **LUT Min Value** correspond to the first color in the LUT, while values higher than the **LUT Max Value** are associated with its last color. Values between the two are linearly mapped with the index of the colors contained in the LUT. In the **diffusion mode**, the trajectory diffusion coefficients are computed in log scale before being mapped. In the **length mode**, the trajectory duration (in time frames) is used for the mapping. The method used to compute the instant and global diffusion coefficients will be explained in section 13.4 below.

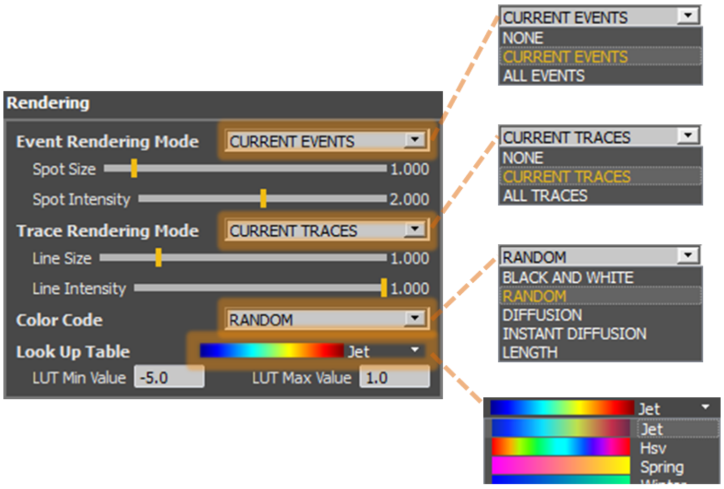

Figure 437 Trajectory rendering interface

13.3 Trajectory filtering

FluoSim allows you to filter the trajectories in function of their diffusion coefficient and their duration. It can be used to visualize more or less mobile molecules or short versus long trajectories, and select them for further analysis and data export. This is done by adjusting the **Minimum and Maximum diffusion coefficients** expressed in log scale (Log D), and the **Minimum and Maximum Length parameters**, respectively.

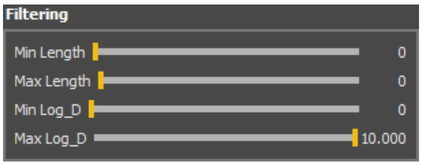

Figure 50 Trajectory filtering interface

13.4 Advanced parameters

Diffusion: the diffusion coefficients are calculated from the **Mean Squared Displacement (MSD)** function associated with trajectories lasting more than 10 frames. A linear fit is performed on the first points of the MSD to retrieve the diffusion coefficient. The number of points being fitted is by default equal to 4 but can be changed using the **D Nb Points Fit** parameter. These points corresponds to the

MSD associated with the displacements occurring during one frame to D Nb Points Fit frames: {MSD(1), MSD(2),…,MSD(D Nb Points Fit)}.

Instant Diffusion: these diffusion coefficients correspond to local diffusion coefficients which are evaluated along the trajectories, in the vicinity of each localization, to quantify changes in the diffusive behavior. To calculate these coefficients, a window centered on each position is used so that only sub-trajectories are considered. The length of these sub-trajectories is determined by the parameters **DInst Nb Points Before Fit** and **DInst Nb Points After Fit** which specify the number of time points considered before and after the actual position, respectively, to calculate the instant diffusion coefficient. Hence the window is made of DInst Nb Points Before Fit + DInst Nb Points After Fit + 1 positions (i.e. DInst Nb Points Before Fit + DInst Nb Points After Fit displacements). These sub-trajectories are then analyzed using the MSD method to extract a diffusion coefficient by fitting the first D Nb Points.

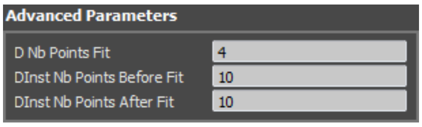

Figure 51 **Advanced parameters**

13.5 Exporting Data

Properties per track:

FluoSim gives you the possibility to export the properties associated with the trajectories: their instantaneous and global coefficient of diffusion and their MSD.

Diffusion per track: a single file containing all diffusion coefficients is generated, with one value per line. The diffusion coefficients are calculated from the MSD function associated with trajectories lasting more than 10 frames. A linear fit is performed on the first points (i.e. D Nb Points Fit points).

MSD per track: a file containing the MSD is exported, each line corresponding to a single trajectory. The first column contains the index of the trajectory, the second the trajectory length, the third the number of MSD points being exported and the next columns give the MSD points. Hence a MSD file contains N+3 columns, where N stands for the number of MSD values being exported which can be set using the parameter **Nb MSD points**.

Instant diffusion per track: a single file containing all the instant diffusion coefficients is generated, each line corresponding to a single trajectory. The first column contains the index of the trajectory, the second column the trajectory length and the next columns give the instant diffusion coefficients. Since these coefficients are deduced from the analysis of sub-trajectories, when such sub-trajectory cannot be formed due to a lack of points (missing points before or after the considered position) the coefficient is set to -1.

Histograms:

FluoSim allows you to export histograms of the trajectory length and of the global or instantaneous diffusion coefficients as text files permitting comparison with actual experiments. The first column of these files gives the bins being used and the second column the number of occurrences associated with these bins: it corresponds to the number of values comprised between two consecutive bins. The range of the histogram can be adjusted using the values **Min Diffusion and Max Diffusion** and the number of bins is determined by the parameter **Nb Intervals**.

Diffusion Histograms: the first column of the exported file contains the Diffusion Coefficient in log scale, and the second column contains the number of trajectories in each bin.

Instant Diffusion Histograms; the first column of the exported file contains the Instant Diffusion Coefficient in log scale and the second column contains the number of sub-trajectories associated with each bin.
Length Histograms: the first column of the exported file contains the trajectory length (in time frames), and the second column contains the number of trajectories associated with these bins.

Filtered Trajectories:
Trajectories Export: FluoSim can save the filtered trajectories in different formats, which have been described in the File Formats section (3.1).
Screenshot: a TIFF file containing a copy image of the display window is exported.

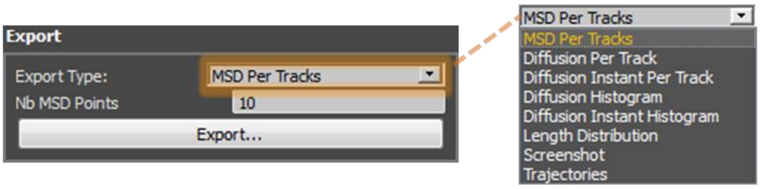

Figure 52 **Export interface**

14. FluoSim projects

FluoSim uses **project files (.pro)** to save and retrieve the simulation parameters. These files contain the region geometries and their types, the dynamic and photophysical parameters, the rendering parameters and the Live and Recorded Experiment characteristics (Experiment Type, pre-sequence, duration,…). You can load or save a project from the File Menu by clicking respectively on "Load Project" or "Save Project.

15. Examples

In this section, we go through a few examples of the experiment types which can be simulated with FluoSim. These examples containing pre-defined sets of parameters that are saved as project files in the Examples directory contained in the installation folder. They can be directly loaded in FluoSim via the Examples menu, or opened via the command: File > Load Project > Resources > Examples. Alternatively, the projects can be entered manually by starting with a new project and importing the images and regions contained in the Resources directory, and defining the parameters one by one as described below. They all refer to the experimental system described in the accompanying paper, namely COS-7 cells making heterologous adhesions between neurexin-1β and neuroligin-1.

15.1 SPT Experiment

This example demonstrates how to perform the SPT simulation which has been used to fit the experimental data obtained on membrane neurexin1β sparsely labeled with Atto-674-conjugated anti-GFP nanobody and making contact - or not- with Neuroligin1-mcherry present in co-cultured COS-7 cells.

1) Load the SPT project

Select the **SPT Example** from the menu **Examples > SPT Experiment or File > Load Project > Resources > Examples > SPT**. This project opens in the Geometry section where regions are displayed in blue. 3 regions appear in the region tab. Region0 is the contour region (C), Region1 represents the trapping area (T), and Region2 represents the rest of the cell where molecules diffuse freely and is used for

measurement (no letter). Alternatively, start a new project directly from the GEOMETRY menu by clicking on Import Geometries and choosing the SPT.rgn from the directory FluoSim\Resources\Examples. Also import from the same folder the corresponding background image SPT.tif and click on the box Display Image. You should now see the three regions super-imposed on the background image as shown below.

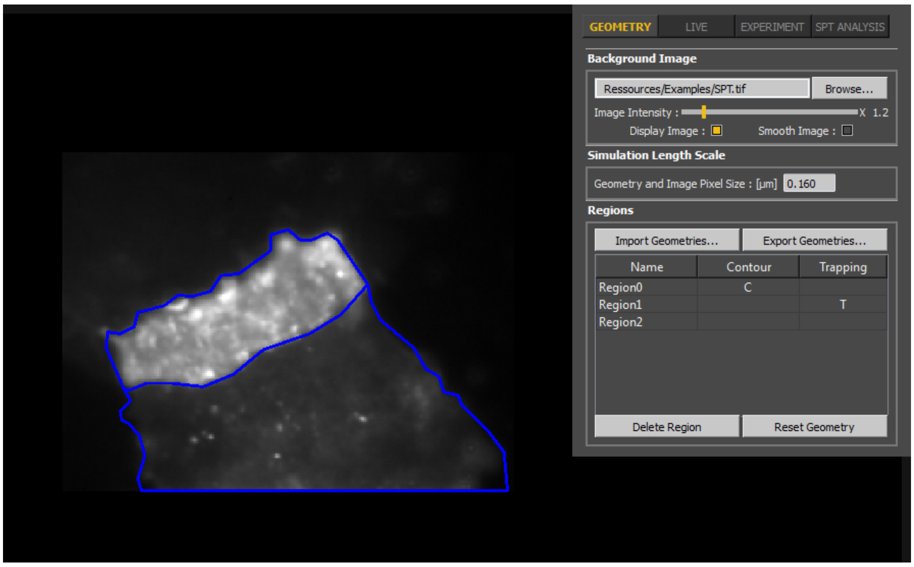

2) Simulation parameters live

Go to the LIVE section to visualize the molecules and check the simulation parameters which should be filled if you opened the SPT example, or enter them yourself one by one. The parameters corresponding to the situation that best fit our SPT experiments, are: Number of steps = 2200; Simulation Time Step= 0.02 s; Copy Number = 250 molecules; Diffusion Outside = 0.3 µm²/s; Diffusion Inside = 0.3 µm²/s; Crossing Probability = 0.7; Binding rate = 0.15 s^{-1}; Unbinding rate = 0.015 s^{-1}; Diffusion Trapped = 0.04 µm²/s, Immobile Fraction = 5%, No fixation; Switch-on Rate = 1 s^{-1}; Switch-off Rate = 3 s^{-1}; Motif: GAUSSIAN; Color: WHITE; Spot size = 0.192 µm; Spot Intensity = 5000 grey levels. When a project is loaded, a Steady State evaluation has been performed by default. If you entered the project manually, click now on the Evaluate Steady State button. Select the Color TRAPPING to visualize bound Nrx1β molecules in red, and freely diffusing molecules in green. Check by pressing the Play button that molecules are indeed enriched in the trapping area and that they undergo reversible photo-switching, diffusing fast in Region 3, and more slowly in Region 2.

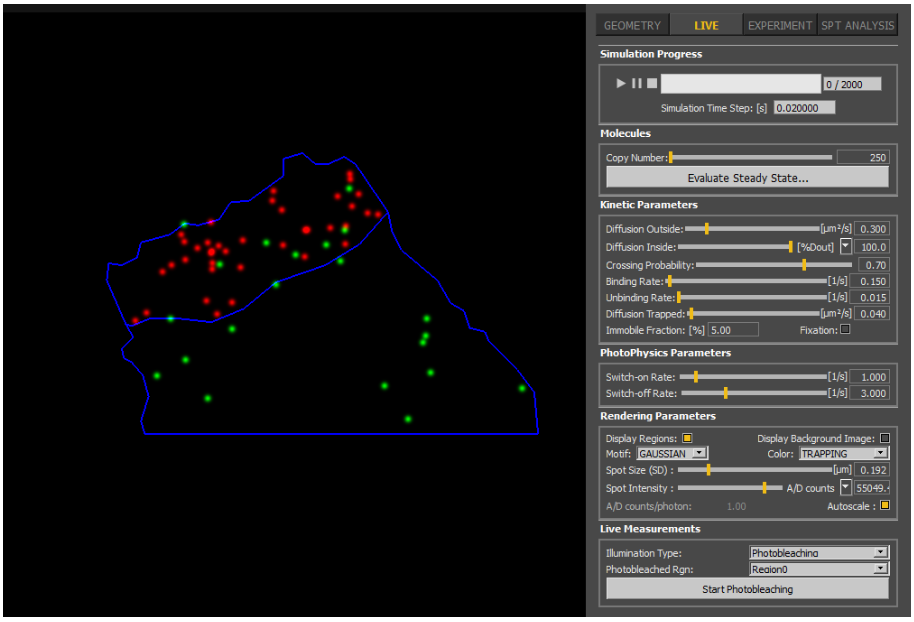

3) Simulate an SPT Experiment

Go to the EXPERIMENT section. Trajectories present in the 3 regions will be recorded. They correspond to the whole set of molecules (Region0), those diffusing inside (Region1) and outside of the contact (Region2). If you defined the project manually, select the following parameters: Experiment SPT, Acquisition Mode: STREAM, Presequence: 200 frames; Duration 2200 frames, Repetition: 1. Also Add one by one the three Measured regions: Region 0, Region1, and Region 2. You can launch the simulation by clicking on the play button. The default destination folder to save the trajectories should the “Simulation Outputs” folder contained in the installation directory. At the end of the simulation, trajectories will be exported as a .trc file into this folder. Alternatively, if you want to introduce noise in the system, choose the option Export Stack (see section 12.2). In the LIVE menu under Rendering Parameters, choose a peak signal in photons/sec (e.g. 2000) with a specific gain in counts/photon (e.g. 20). In the EXPERIMENT menu, select Poisson Noise with a background level in Photons/sec (e.g. 200). Finally, you can add a camera offset with a readout noise, both in grey levels (e.g. 500 and 10 A/D counts, respectively). Then, manually analyze the intensity fluctuations in the region of interest after opening the Exported stack in an another software. This option can be useful to test the capacities of single molecule tracking software, or to generate large datasets for the training of deep learning algortithms for image reconstruction.

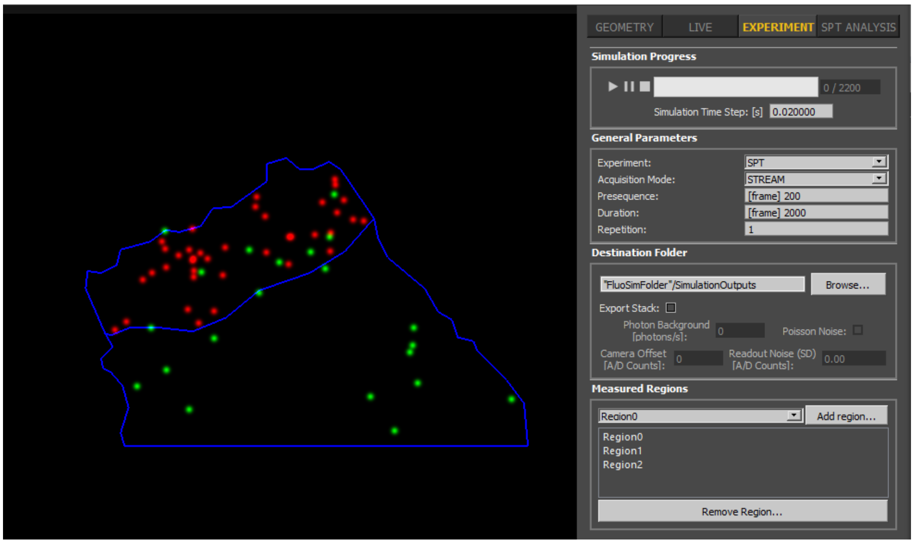

4) Visualize trajectory and export histograms

Go to the SPT analysis section. Load one of the trajectory files (.trc) that have been saved in the destination folder after the SPT simulation. Three files should be present, corresponding to the trajectories recorded in the three regions: full cell, contact region, and outside region. First load the full cell region (rgn0 file).

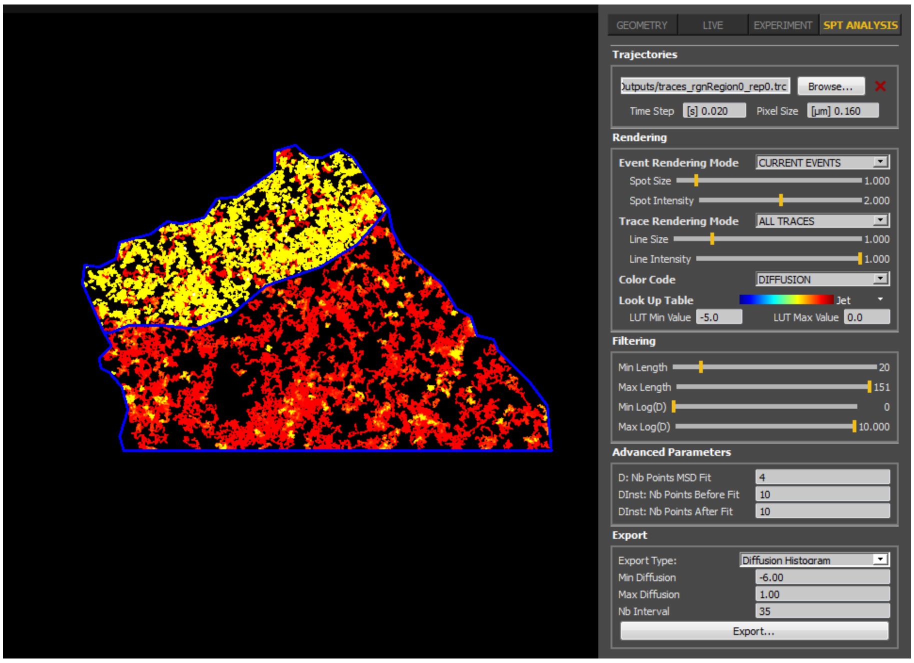

If you export the diffusion coefficient histogram with 35 bins, with minimum Log(D)= -6 and maximum Log(D) = 1, a file called DHist_n35_mn-6_mx1.txt should appear in the Simulation Output folder. Opening this file in Excel and plotting it should give you the following graph, where the x-axis if the diffusion coefficient in logarithmic scale and the y-axis is the number of trajectories. One can distinguish three peaks, the first one corresponding to the pool of freely mobile molecules (Log(D) = -

0.5, the second one to pool of molecules trapped in the cell-cell contact (log(D) = -1.4, and the third one to immobile molecules (log(D) = -5).

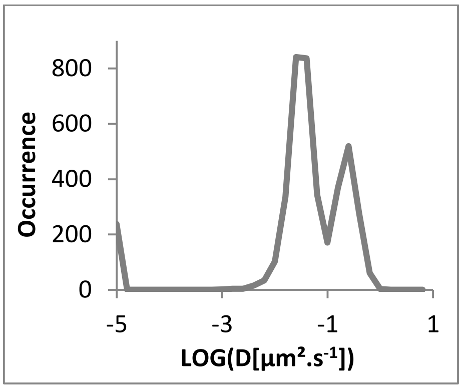

15.3 FRAP experiment

This example demonstrates how to perform the FRAP simulation which has been used to fit the experimental data obtained on GFP-Neurexin1β making contact – or not – with Neuroligin1-mCherry expressing cells.

1) Load a FRAP project

Select the **FRAP Example** from the **Examples > FRAP-Experiment > Contact or open File > Load Project > Resources > Examples > FRAP (in or out)**. This project is opened in the GEOMETRY section where regions are displayed. 3 regions appear in the region tab. Region0 is the contour region (C), Region1 is the trapping area (T): this is our cell-cell contact. The circular Region 2 in the contact will be photobleached and used to measure the fluorescence recovery. Alternatively, start a new project directly from the GEOMETRY menu by clicking on Import Geometries and choosing the FRAP-in.rgn or FRAP-out.rgn from the directory FluoSim\Resources\Examples. Also import from the same folder the corresponding background image FRAP-in.tif or FRAP-out.in, respectively, and click on the box Display Image. For FRAP-in, you should now see the three regions super-imposed on the background image as shown below.

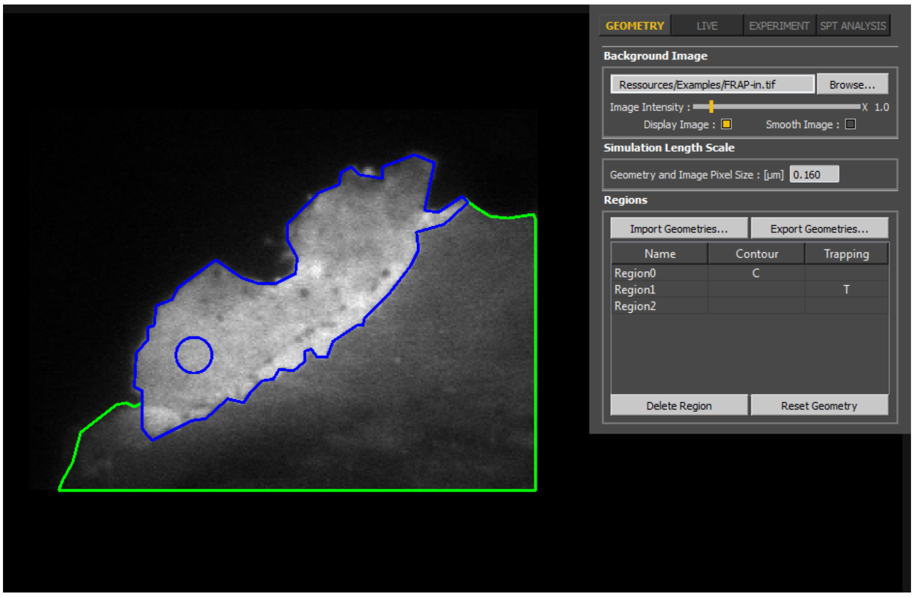

2) Check the FRAP experiment live

Go to the LIVE section to visualize the molecules and check the simulation parameters which should be filled if you opened the FRAP example, or enter them yourself one by one. These parameters correspond to the situation which has been used to fit our FRAP experiments, and are: Number of steps = 1000; Simulation Time Step= 0.1 s; Copy Number = 100,000 molecules; Diffusion Outside = 0.3 µm²/s; Diffusion Inside = 0.3 µm²/s; Crossing Probability = 0.25; Binding rate = 0.15 s^{-1}; Unbinding rate = 0.015 s^{-1}; Diffusion Trapped = 0.04 µm²/s, Immobile Fraction = 0%, No fixation; Switch-on Rate = 0 s^{-1}; Switch-off Rate = 0 s^{-1}; Motif: GAUSSIAN; Color: WHITE. Since FRAP is an ensemble measurement, many molecules (100,000 copies) have be introduced here to limit the intensity fluctuations. A Steady State Evaluation has been performed by default, resulting in a ~3-fold molecular enrichment in the cell-cell contact. If you defined the project manually, click now on the Evaluate Steady State button. A graph showing the Intensity in Region2 over time should appear on the left corner, otherwise select Window > ShowPlot. Under Live Measurements, select Illumination Type: Photobleaching and Photobleached Rgn: Region2. Click on the Play button to start the simulation. By pressing the button Start Photobleaching located at the bottom of the parameter panel, the fluorescence should drop to zero in one frame in the circular region, then slowly recover.

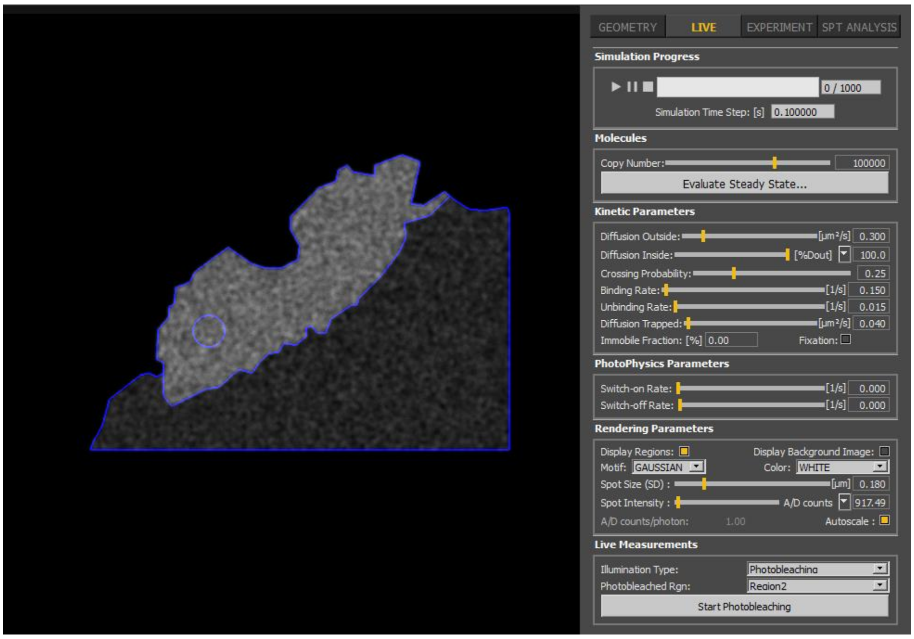

3) Simulate a FRAP experiment

Go to the EXPERIMENT section. If you defined the project manually, select the following parameters: Experiment FRAP, Acquisition Mode: STREAM, Presequence: 100 frames; Duration 1200 frames, Repetition: 1. Also add the Measured Region 2. Since experimentally 75% of the initial fluorescence is bleached during the 400 ms laser exposition, the "Bleaching Number Frames" is set to 4 (Δt=0.1 s) and the "Bleaching Rate" to 4.25 s^{-1}. Region2 will be bleached (see Bleaching Region Tab) and measured (see Measured Region Tab). You can launch the simulation by clicking on the play button. At the end, intensity files will be exported in the destination folder "Simulation Outputs".

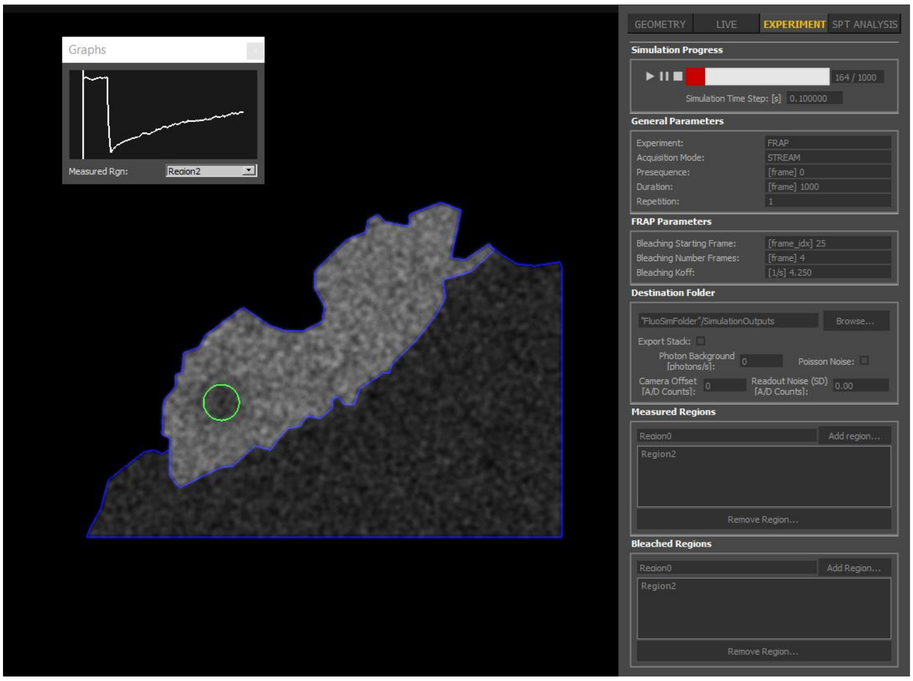

You can then open the simulation output graph in an Excel file. The x axis is the time in sec, and the y axis is the fluorescence intensity (number of molecules divided by the surface area of the recorded region).

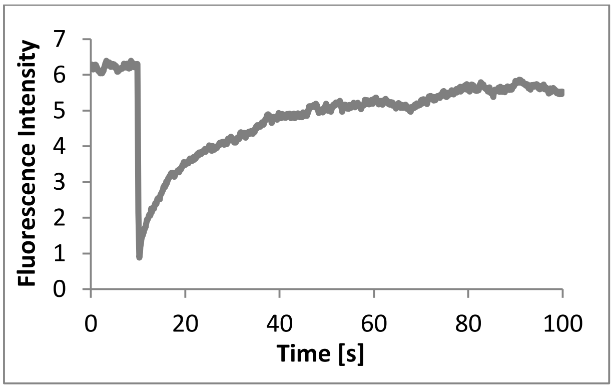

15.4 PAF experiment

This example demonstrates how to perform the PAF simulation which has been used to fit the experimental data obtained on PAGFP-Neurexin1β making contact – or not – with Neuroligin1-mCherry expressing cells.

1) Load a PAF project

Select the **PAF Example** from the **Example > PAF-Contact Experiment menu or File > Load Project > Resources > Examples > PAF (in or out)**. This project is opened in the GEOMETRY section where regions are displayed. 3 regions appear in the region tab. Region0 is the contour region (C), Region1 is the trapping area (T): this is our cell-cell contact. The circular Region 2 in the contact will be photoactivated and used to measure the fluorescence decay. Alternatively, start a new project directly from the GEOMETRY menu by clicking on Import Geometries and choosing the PAF-in.rgn or PAF-out.rgn from the directory FluoSim\Resources\Examples. Also import from the same folder the corresponding

background image PAF.tif and click on the box Display Image. For PAF-in, you should now see the three regions super-imposed on the background image as shown below.

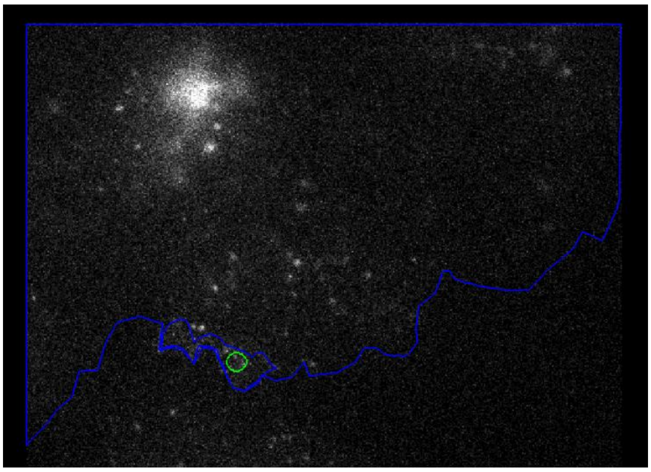

2) Check the live PAF experiment

Go to the LIVE section to visualize the molecules and check the simulation parameters which should be filled if you opened the PAF example, or enter them yourself one by one. These parameters correspond to the situation which has been used to fit our PAF experiments, and are: Number of steps = 1300; Simulation Time Step= 0.1 s; Copy Number = 150,000 molecules (with contact) and 72,000 (no contact); Diffusion Outside = 0.18 $\mu m^2/s$; Diffusion Inside = 100%; Crossing Probability = 0.25; Binding rate = 0.15 s^{-1}; Unbinding rate = 0.015 s^{-1}; Diffusion Trapped = 0.04 $\mu m^2/s$, Immobile Fraction = 0%, No fixation; Switch-on Rate = 0 s^{-1}; Switch-off Rate = 0 s^{-1}; Motif: POINT; Color: WHITE. A Steady State Evaluation has been performed by default, resulting in a ~3-fold molecular enrichment in the cell-cell contact. If you defined the project manually, click now on the Evaluate Steady State button. To put all molecules in a non-fluorescent state initially, increase the Switch-off Rate to 10 s^{-1} for a few seconds after pressing the Play button, then set it back to 0 s^{-1} and stop the simulation: the whole cell should be dark. A graph showing the Intensity in Region2 over time should appear on the left corner, otherwise select Window > ShowPlot. Under Live Measurements, select Illumination Type: PhotoActivation and PhotoActivated Rgn: Region2. Click on the Play button to start the simulation. By pressing the button Start PhotoActivation located at the bottom of the parameter panel, the fluorescence should increase in one frame in the circular region (as shown below), then slowly decay.

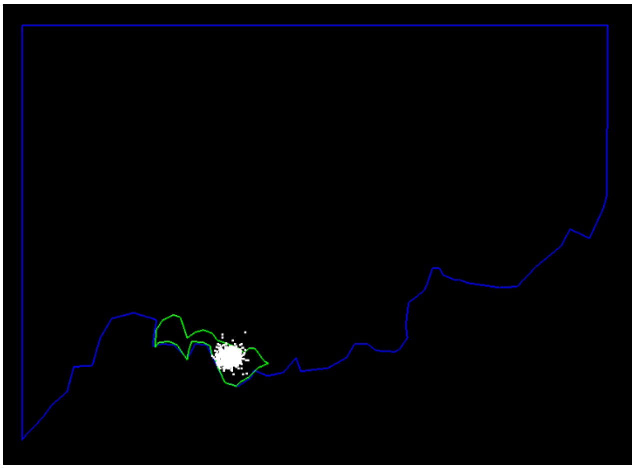

3) Simulate a PAF experiment

After sliding again the Switch-off Rate to 10 s^{-1} and back to 0 s^{-1} in the Play mode, go to the EXPERIMENT section. If you defined the project manually, select the following General Parameters: Experiment PAF, Acquisition Mode STREAM, Presequence 100 frames; Duration 1200 frames, Repetition 1. In the PAF parameters, select Activation Starting Frame 25; Activation Number Frames 3 (total 300 ms) and Activation rate 1.0 s^{-1}. Also add Region 2 in the Measured Regions and the Photoactivated Regions tab. You can now launch the simulation by clicking on the play button. At the end of the simulation, the text file "averageIntensity_rgnRegion2" will be exported in the destination folder "Simulation Outputs".

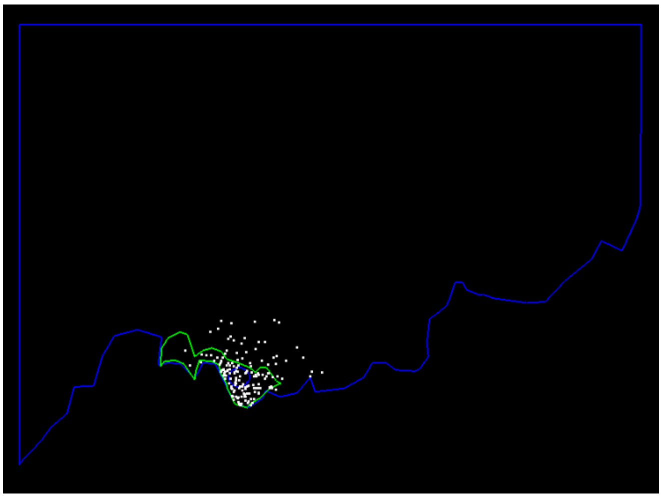

You can then open the simulation output graph in an Excel file. The x axis is the time in sec, and the y axis is the fluorescence intensity (number of molecules divided by the surface area in px^2 of the recorded region).

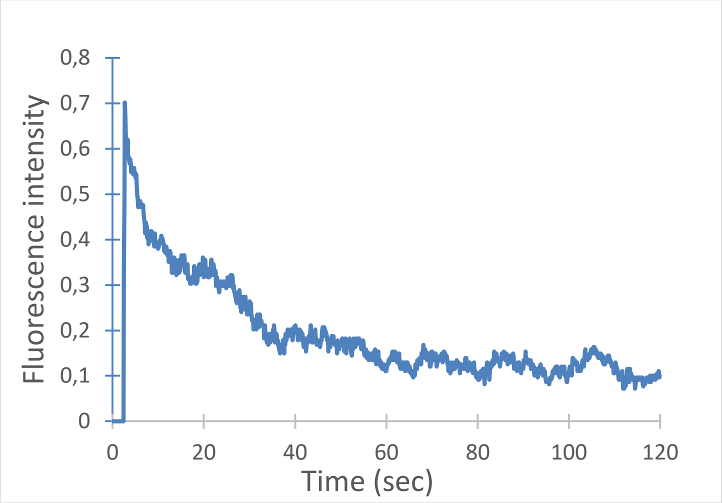

Alternate take: Simulate a PAF experiment using the Stack Export option. Instead of setting the fluorescence level to zero before photoactivation, one might want to take into account the background signal due to cell auto-fluorescence or residual emission of PA-GFP before photoactivation, that can be used to normalize the fluorescence signal after photoactivation. In that case, it is interesting to introduce some noise in the sensor, and represent molecules by their Gaussian intensity profiles. To simulate the photoactivation of PAGFP-Nrx1β, we defined a peak intensity of 1000 grey levels (A/D counts), and a spot size of 0.171 µm under LIVE menu \ Rendering Parameters, and in the EXPERIMENT menu\ Image Noise and Export, we introduced a camera offset of 500 grey levels with a Readout noise of 20 A/D counts. With these parameters, we mimicked the 10- or 3-fold increase in fluorescence intensity with respect to background upon photoactivation of PAGFP-Nrx1β in contact or free regions, respectively (the difference between the two situations is due to molecular enrichment in the adhesive contact). Here is below a still image from the Exported stack, later opened in Metamorph.

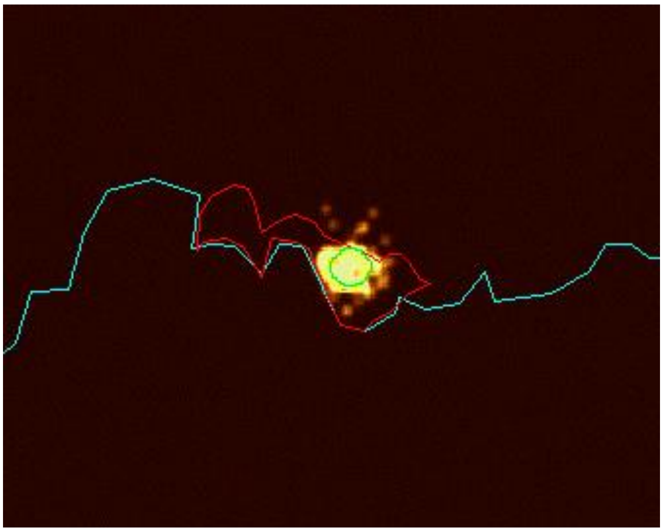

15.5 FCS Experiment

This example demonstrates how to perform the FCS simulation which has been used to fit our experimental data obtained on GFP-Neurexin1β labeled with intermediate dilution of Atto-647N conjugated nanobody, in contact – or not – with Neuroligin-1-mCherry expressing COS-7 cells.

1) Load FCS project

Select the **FCS Example** from the menu **Examples > FCS Experiment > Contact or File > Load Project > Resources > Examples > FCS (in or out)**. The project FCS-in is opened in the Geometry section where regions are displayed. 3 regions appear in the region tab. Region0 is the contour region (C), Region1 is a trapping region (T): the cell-cell contact, and Region3 is used to record the intensity fluctuations and calculate their autocorrelation function. Alternatively, start a new project directly from the GEOMETRY menu by clicking on Import Geometries and choosing the FCS-in.rgn or FCS-out from the directory FluoSim\Resources\Examples. Also import from the same folder the corresponding background image FCS-in.tif or FCS-out and click on the box Display Image. For FCS-in, you should now see the three regions super-imposed on the background image as shown below.

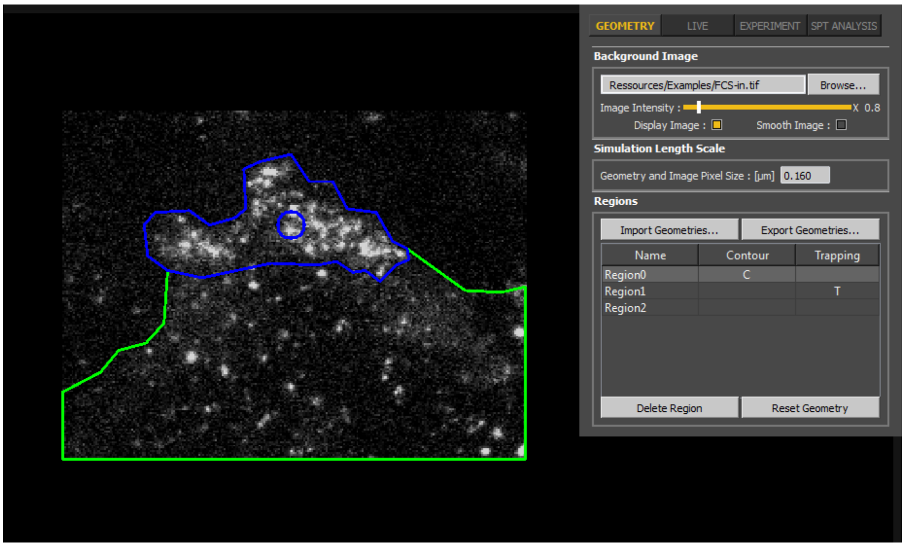

2) FCS parameters

Go to the LIVE section to visualize the molecules and check the simulation parameters which should be filled if you opened the FCS example, or enter them yourself one by one. These parameters correspond to the situation which was used to fit our FCS experiments, and are: Number of steps = 100000; Simulation Time Step= 0.005 s; Copy Number = 200 molecules; Diffusion Outside = 0.3 $\mu m^2/s$; Diffusion Inside = 0.3 $\mu m^2/s$; Crossing Probability = 0.7; Binding rate = 0.15 s^{-1}; Unbinding rate = 0.015 s^{-1}; Diffusion Trapped = 0.04 $\mu m^2/s$, Immobile Fraction = 0%, No fixation; Switch-on Rate = 0 s^{-1}; Switch-off Rate = 0 s^{-1}; Motif: GAUSSIAN; Color: WHITE; Spot size 0.2 µm; Spot intensity: 4000 grey levels. Since the fluctuations have to span a time window of several orders of magnitude (~5 decades), many time frames have to be simulated (here: 100,000). Small time steps are chosen (2 ms for cells showing no adhesive contact, and 5 ms for cells forming adhesive contacts). A Steady State Evaluation has been performed by default, resulting in a ~2-fold molecular enrichment in the cell-cell contact. If you defined the project manually, click now on the Evaluate Steady State button. Play the simulation. A graph showing the Intensity fluctuations in Region2 over time should appear on the left corner, otherwise select Window > ShowPlot.

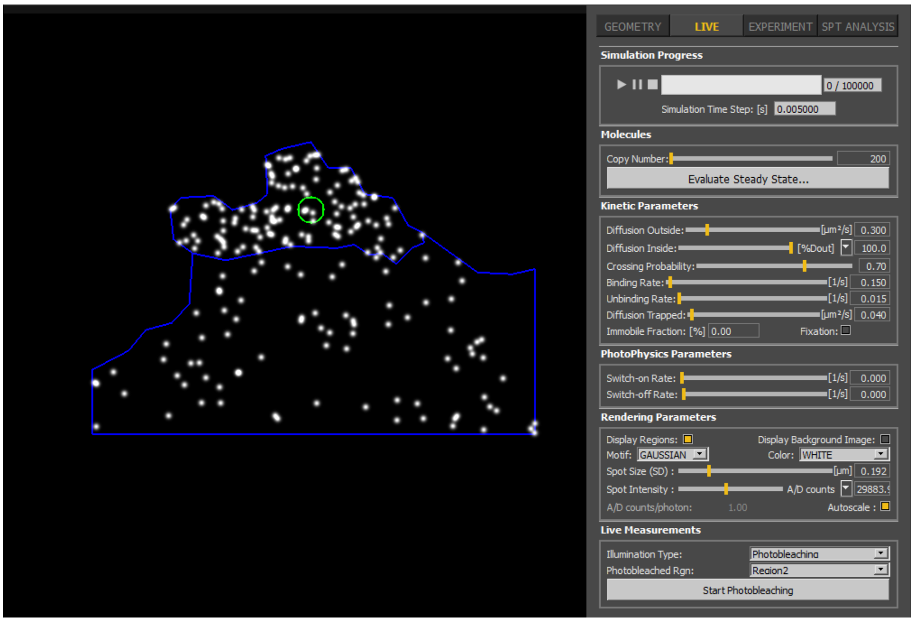

3) Simulate an FCS Experiment

Go to the EXPERIMENT section. If you defined the project manually, select the following parameters: Experiment FCS, Acquisition Mode: STREAM, Presequence: 0 frames; Duration 100,000 frames, Repetition: 1. Also add the Measured Region 2. Since a 647-nm focused laser beam was used experimentally to excite the anti-GFP Nanobody-Atto647 bound to GFP-Nrx1β molecules, the Gaussian Beam option is enabled. Its size has been set to fit the standard deviation of the experimental Gaussian intensity profile (0.255 µm), the Beam maximum intensity was set to 1, and the Beam bleaching rate was set to 0.4 s^{-1}, to mimic the mild photobleaching of molecules staying in the laser beam. Now launch the simulation by clicking on the play button. At the end of the simulation, intensity fluctuations and autocorrelation files will be exported in the folder Simulation Outputs.

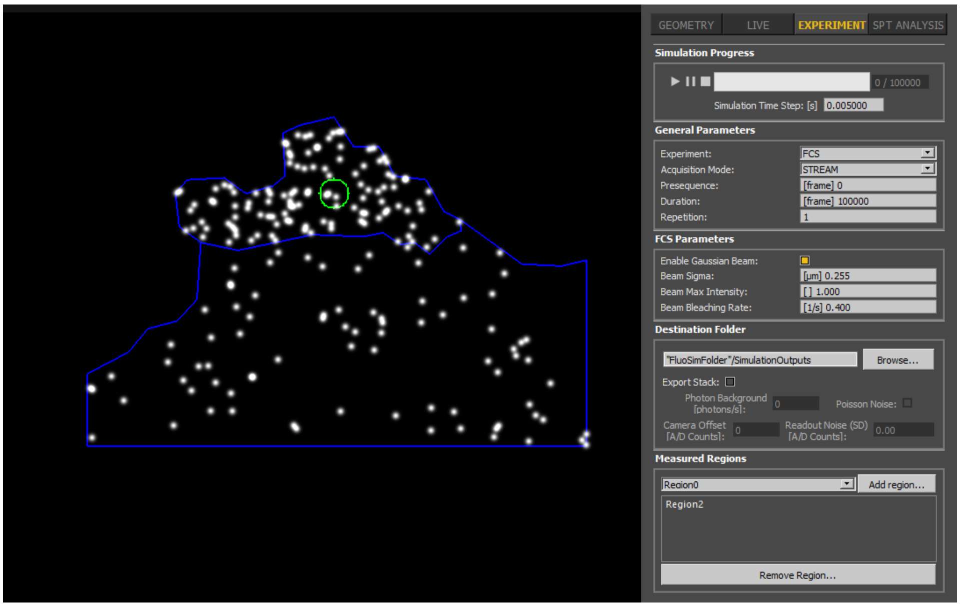

You can then open the simulation output graph in an Excel file. The intensity fluctuations appear as a graph named "*averageIntensity_rgnRegion2_rep0*" and show the number of molecules divided by the region area over time (in sec).

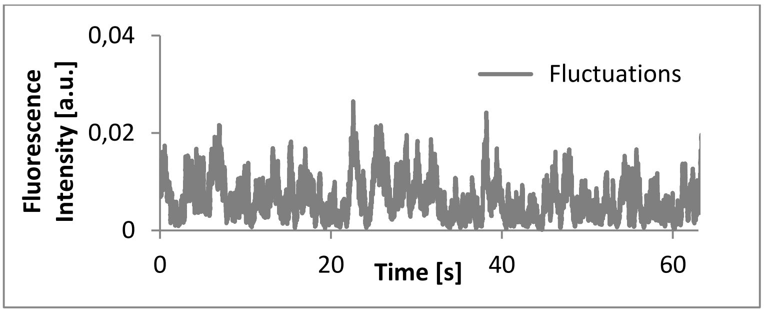

The second graph is named "correlation_rgnRegion2_rep0" x axis is the time in sec in a logarithm scale, and the y axis is the autocorrelation function after normalization with the first 5 values.

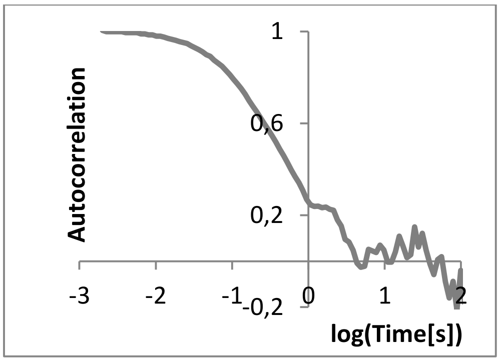

You might want to introduce additional noise in the system (see section 12.2). If so, select the option Export Stack. In the LIVE menu under Rendering Parameters, choose a peak signal in photons/sec with a specific gain in counts/photon. In the EXPERIMENT menu, select Poisson Noise with a background level in Photons/sec. Finally, you can add a camera offset with a readout noise, both in grey levels (A/D counts). Then, manually analyze the intensity fluctuations in the region of interest after opening the Exported stack in an another software.

15.6 SRI Experiment

This example demonstrates how to perform the SRI simulation which has been used to fit the experimental STORM image of the GFP-Neurexin-1β labeled with Nanobody Alexa-647 in contact with NLG1-mcherry.

1) Load SRI project

Select the **SRI Example** from the **Examples > SRI Experiment** or from **File > Load Project > Resources > Examples > SRI**. This project is opened in the Geometry section where the geometry is displayed. 2 regions appear in the region tab. Region0 is the contour region (C), while Region1 is a trapping region (T): the cell-cell contact. Alternatively, start a new project directly from the GEOMETRY menu by clicking on Import Geometries and choosing the SRI.rgn from the directory FluoSim\Resources\Examples. Also import from the same folder the corresponding background image SRI.tif and click on the box Display Image. You should now see the two regions super-imposed on the background image as shown below.

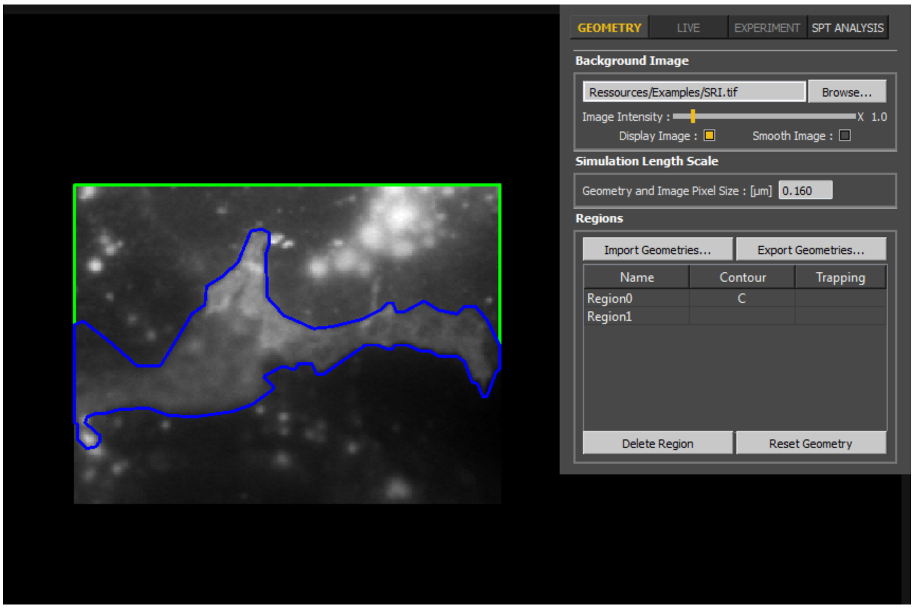

2) SRI parameters

Go to the LIVE section to visualize the molecules and check the simulation parameters which should be filled if you opened the FCS example, or enter them yourself one by one. These parameters correspond to the situation which has been used to fit the STORM experiment and are: Number of steps = 10200; Simulation Time Step= 0.02 s; Copy Number = 70,000 molecules; Diffusion Outside = 0 $\mu m^2/s$; Diffusion Inside = 0 $\mu m^2/s$ (100% x D_{out}); Crossing Probability = 0.36; Binding rate = 0.15 s^{-1}; Unbinding rate = 0.015 s^{-1}; Diffusion Trapped = 0 $\mu m^2/s$, Immobile Fraction = 0%, Fixation: checked; Switch-on Rate = 0.006 s^{-1}; Switch-off Rate = 9.3 s^{-1}; Motif: POINT; Color: WHITE. To mimic fixation, all diffusion coefficients have been set to zero. The number of molecules introduced in the system (70000) has been estimated from the number of detections in the STORM image and the photophysics of the Alexa647 fluorophores. A Steady State Evaluation has been performed by default, resulting in a ~3-fold molecular enrichment in the cell-cell contact. If you defined the project manually, click now on the Evaluate Steady State button. Play the simulation, you should see a rapid fluorescence loss, followed by the blinking of fixed fluorophores.

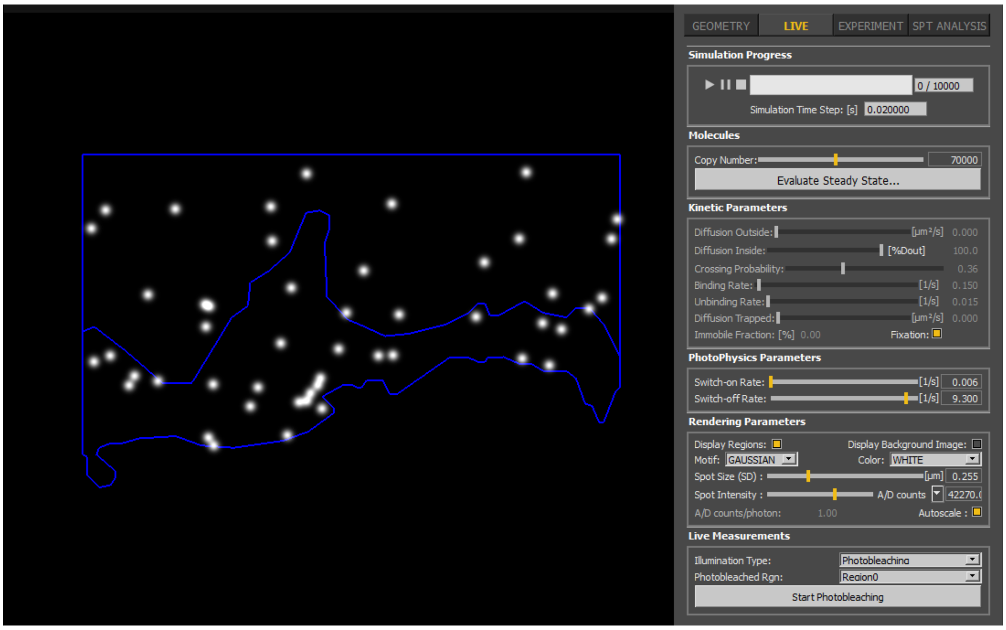

3) SRI Experiment

Go to the EXPERIMENT section. If you defined the project manually, select the following parameters: Experiment SRI, Presequence: 200 frames; Duration 10200 frames, Repetition: 1. A large number of frames (10,000) has been introduced to match the long experimental acquisition sequence (performed at 50 Hz, i.e. Δt = 0.02 s). Launch the simulation by clicking on the play button. An image should appear which gets progressively populated with single molecule localizations. The image is zoomed 5-fold compared to the original low resolution image. The localization precision (0.025 µm) corresponds to the standard deviation (SD) of the Gaussian spatial distribution of the detections around the molecule actual position and is used to disperse the detections around the positions of the fixed molecules. The intensity of each detection is set to 1, but one can increase it to 10 or 100, it will just enhance the total grey levels of the generated 16-bit image. At the end of the SRI simulation, a super-resolved TIFF image named SRI_screen_capture_rep0 is generated and saved in the Simulation Outputs folder.

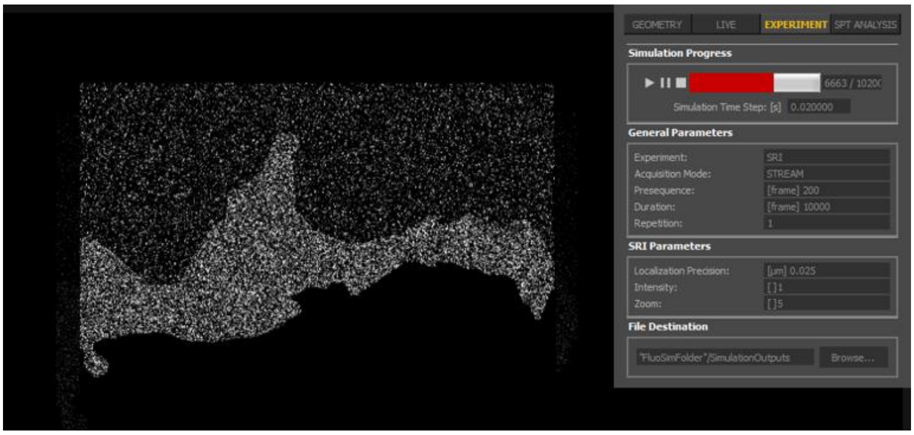

15.7 Single molecule pull-down

This example demonstrates how to perform the Single Molecule Pull-down simulation which was used to fit the experimental data obtained on GFP-neurexin1β immobilized on a glass substrate and thereby validate the different noise levels introduced in the simulated images.

1) Load the Single Pull project

Select the **Single Pull Example** from the menu **Examples > SPT Experiment or File > Load Project > CellSamples > Single Pull**. This project opens in the Geometry section where a single square region of 256 x 256 pixels representing the camera chip is displayed in blue (pixel size = 0.25 µm). The contour region (Region0) contains 100 immobilized molecules placed at random and considered immobile. Alternatively, start a new project directly from the GEOMETRY menu by clicking on Import Geometries and choosing the Single-Pull.rgn from the directory FluoSim\Resources\Examples. Also import from the same folder the corresponding background image Single Pull.tif and click on the box Display Image. You should now see the region super-imposed on a black background image.

2) Simulation parameters live

Go to the LIVE section to visualize the molecules and check the simulation parameters which should be filled if you opened the Single Pull example, or enter them yourself one by one. The parameters corresponding to the situation that best fit our Single Molecule Pull-down experiments, are: Number of steps = 500; Simulation Time Step= 0.03 s; Copy Number = 100 molecules; Diffusion Outside = 0 $\mu m^2/s$; Diffusion Inside = 0 $\mu m^2/s$; Crossing Probability = 1; Binding rate = 0 s^{-1}; Unbinding rate = 0 s^{-1}; Diffusion Trapped = 0 $\mu m^2/s$, Immobile Fraction = 0 %, No fixation. Alternatively, you can click on the Fixation box, which will set all diffusion coefficients to zero. The photophysics parameters are: Switch-on Rate = 0 s^{-1}; Switch-off Rate = 0.28 s^{-1} (corresponding to the 488-nm laser illumination setting that will photobleach single GFP molecules). The rendering parameters are: Motif: GAUSSIAN; Color: WHITE; Spot size = 0.171 µm; Spot Intensity = 210 grey levels (A/D counts) or 350 photons/sec with a gain of 20 (A/D counts/photon). Click on the Play button, you should see the molecules photobleach one by one. You can reset the simulation by clicking on Evaluate Steady State, which will introduce 100 new molecules at random position in the system, or slide the Copy Number to zero, then back to 100.

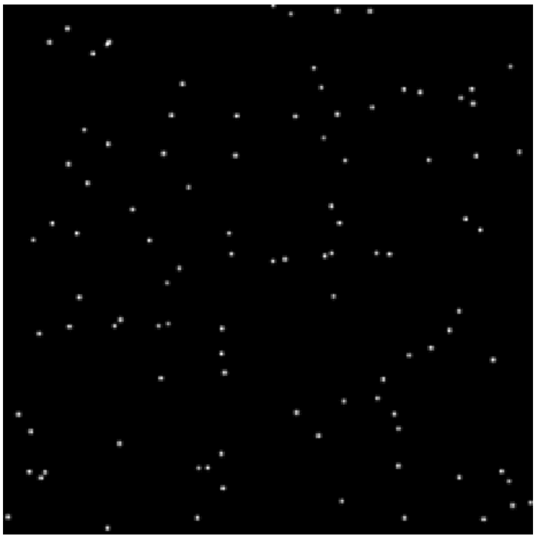

3) Simulate a Single Molecule Pull-down Experiment

Go to the EXPERIMENT section. If you defined the project manually, select the following General parameters: Experiment FRAP, Acquisition Mode STREAM, Presequence 0 frames, Duration 500 frames, Repetition 1. Do not pay attention to the FRAP Parameters, since this mode is chosen here to be able to measure molecular density in given regions, an option which is not accessible in SPT which computes only trajectories. Click on the box Export Stack. Add Region0 in the Measured Regions (but not in the Photobleached or PhotoActivated regions, otherwise the parameters might be altered during the simulation). Then launch the simulation by clicking on the play button. In the default destination folder "Simulation Outputs" contained in the installation directory, there should be a file named averageIntensity_rgnRegion0.txt, and a multipage file named Stack.tiff. The graph below shows the average intensity in the whole image, divided by the surface area in px^2, over time. The curve corresponds to the progressive photobleaching of all GFP molecules, and can be fitted by a single exponentially decreasing function with rate constant k_{off}^{Fluo}.

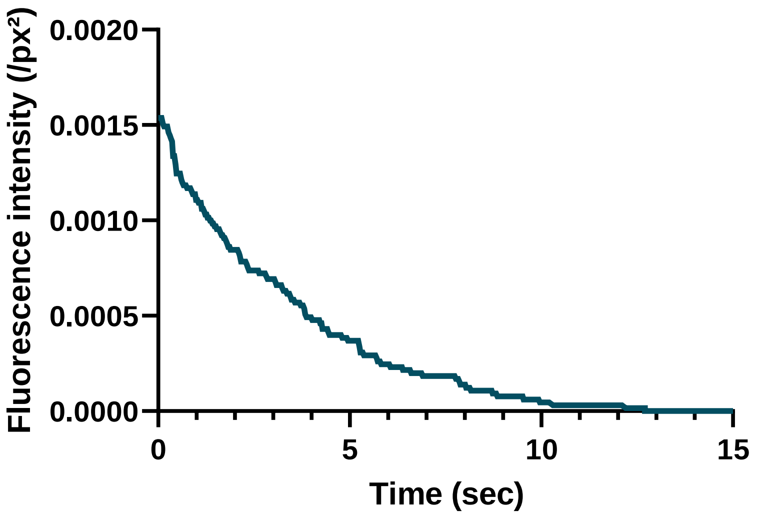

At the single molecule level, molecules exhibit single step photobleaching, as shown in the graph below.

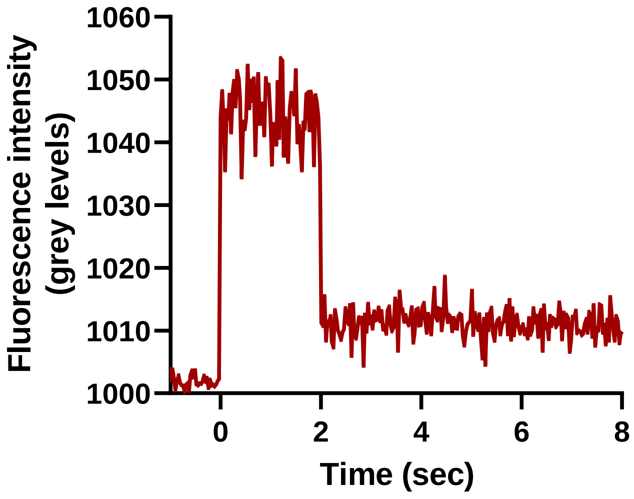

To obtain this simulation, we added different levels of noise to the image. In the LIVE menu under Rendering Parameters, we chose a peak signal of 350 photons/sec with a gain of 20 counts/photon. In the EXPERIMENT menu, we selected Poisson Noise with a background of 20 Photons/sec. Finally, we imposed a camera offset of 1000 grey levels, with a readout noise of 5 A/D counts. Then we opened the exported stack in Metamorph and measured the intensity for each single molecule. The different intensity levels and their fluctuations are very close to those obtained experimentally.

15.8 LTP

Open the long term potentiation example (LTP) by loading the project from CelllSamples\LTP.pro.

GEOMETRY menu
A rectangular geometry will appear, representing a dendritic segment of 2 x 10 µm populated with 500 AMPA-type glutamate receptors, and containing five regularly spaced post-synaptic densities (squares of 0.3 x 0.3 µm) acting as trapping elements. The pixel size is 0.05 µm.

LIVE menu
The basal dynamic parameters are taken from literature values: D_{out} = 0.1 µm²/s, D_{in} = 50% D_{out} = 0.05 µm²/s, D_{trap} = 0.006 µm²/s, $P_{crossing}$ = 0.5, k_{on} = 1.2 s^{-1}, and k_{off} = 0.02 s^{-1}. The time increment is set at Δt = 0.1 s. You can play LIVE with the parameter k_{on} and k_{off} to see how this affects the accumulation of receptors in PSDs.

EXPERIMENT menu
LTP is mimicked by a drop of k_{off} to 0.004 s^{-1} in one frame after a 200 s baseline recording, then kept at this value for 1800 s (30 min). This increases the trapping of AMPA receptors for their post-synaptic scaffold and results in a stable 2-fold accumulation of AMPA receptors at synapses.

Simulation results
Choose the regions in which you want to make the measurements. At the end of the simulations, the output files “averageIntensity_rgnRegion” for each region (1-5), are saved in the destination folder Simulation Outputs. The graph below shows the mean density of AMPA receptors in five synapses over time in the LTP example. A post-synaptic density being defined as a square of 6 x 6 pixels, there are around 1.7 x 36 px² = 60 AMPA receptors per synapse at baseline, and 112 receptors after LTP induction.

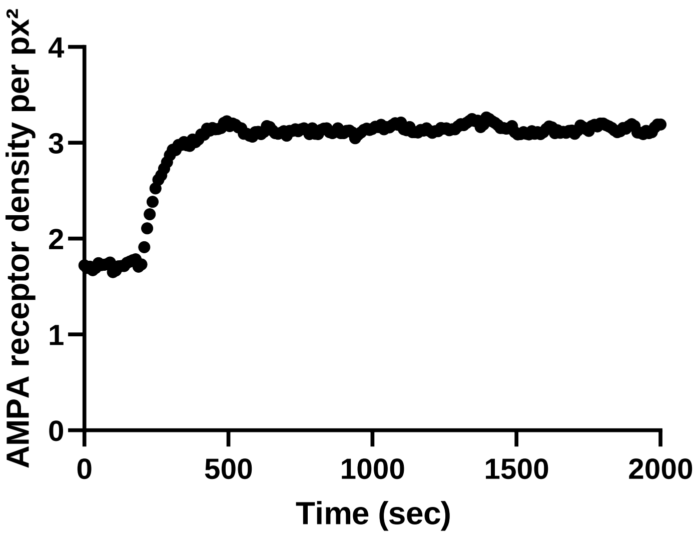

Exported stacks

Alternatively, you can export an image stack and make the measurements offline with another software. Below are two representative images in false color showing the distribution of AMPA receptors in the 2 x 10 µm dendritic segment, before and after LTP induction as being mimicked by a drop in k_{off}. Note that the synaptic receptors are pumped from extra-synaptic pools.

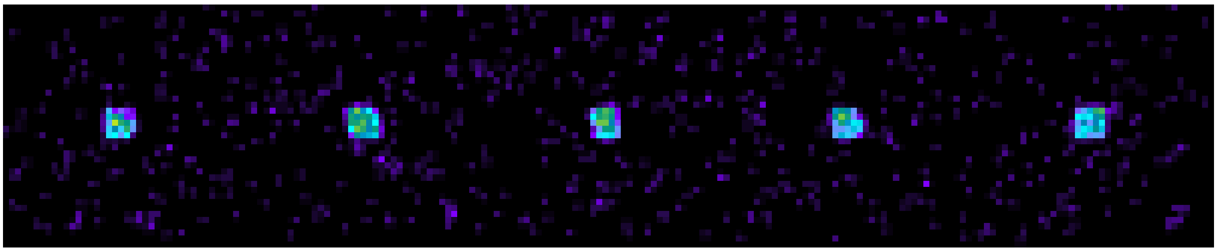

Time 0 (baseline)

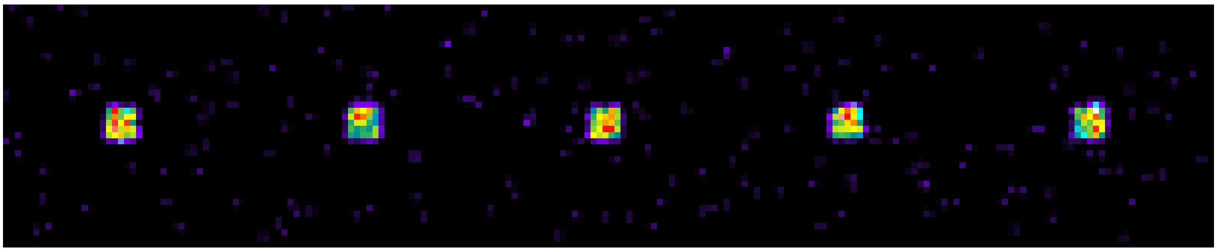

Time 30 min after LTP induction.

Alternatives

On the other hand, one can mimic long term depression (LTD) experiments by decreasing k_{on} or increasing k_{off}, both under the DRUG mode.

16. Rights to use and code accessibility

The software FluoSim (v1.0) is released under a GNU GPL v3 license as supplementary material accompanying this manuscript, and as an archive that can be downloaded at https://www.iins.u-bordeaux.fr/SOFTWARE. The FluoSim (v1.0) release and its source code are available at https://github.com/mlagardere/FluoSim under the same license.

FluoSim is a free software: you can redistribute it and/or modify it under the terms of the GNU General Public License as published by the Free Software Foundation, either version 3 of the License, or (at your option) any later version.

This program is distributed in the hope that it will be useful, but WITHOUT ANY WARRANTY; without even the implied warranty of MERCHANTABILITY or FITNESS FOR A PARTICULAR PURPOSE. See the GNU General Public License for more details.

You should have received a copy of the GNU General Public License along with this program. If not, see <https://www.gnu.org/licenses/>.

FluoSim copyright 2020 Matthieu Lagardère
