# Supplementary material for: FluoSim: simulator of single molecule dynamics for fluorescence live-cell and super-resolution imaging of membrane proteins
Source: Sci Rep. 2020 Nov 17;10:19954. doi: 10.1038/s41598-020-75814-y (PMC7672080; doi:10.1038/s41598-020-75814-y)
Supplement: Supplementary file 2 — Supplementary Information 2. [file 41598_2020_75814_MOESM2_ESM.pdf]

Title: FluoSim: simulator of single molecule dynamics for fluorescence live-cell and super-resolution imaging of membrane proteins

Authors: Matthieu Lagardère[1], Ingrid Chamma[1], Emmanuel Bouilhol[2], Macha Nikolski[2], Olivier Thoumine[1]*

*Corresponding author: olivier.thoumine@u-bordeaux.fr

Affiliations:

1. Univ. Bordeaux, CNRS, Interdisciplinary Institute for Neuroscience, IINS, UMR 5297, F-33000 Bordeaux, France
2. Univ. Bordeaux, CNRS, IBGC, UMR 5095, F-33000 Bordeaux, France

Supplementary text

General description of FluoSim

Programing details

The FluoSim source code was written with the C++ programming language using the ISO/IEC 14882:2011 standard (C++11 standard). The FluoSim program and its related libraries were compiled with the 32bit MinGW compiler (MinGW 4.8 32bit) and can be executed on 32-bit and 64-bit Windows operating systems. The FluoSim project has been developed using the Integrated Development Environment QtCreator 3.0.1.

Libraries

The C++ standard library was used extensively to write the FluoSim source code. The Qt library (Qt 5.2.1) has been used to implement the Graphical User Interface. To allow live rendering of the simulations, FluoSim benefits from hardware acceleration through the OpenGL library (OpenGL 3.2). OpenGL extensions have been loaded with the glew library. Vectors and matrices manipulations utilized in FluoSim calculations are implemented in the glm library. The lmfit library which contains functions to perform non-linear fitting has been used during MSD fitting. Qt, OpenGL, glew and glm libraries are dynamically linked to FluoSim while the lmfit library has been directly integrated in the source code. The program is provided as an executable file within a folder containing the necessary .dll files to operate properly (see the user manual for a description of how to run the program).

Code availability

The software FluoSim (v1.0) is released under a GNU GPL v3 license as supplementary material accompanying this manuscript, and as an archive that can be downloaded at https://www.iins.u-bordeaux.fr/SOFTWARE. The FluoSim (v1.0) release and its source code are available at https://github.com/mlagardere/FluoSim under the same license.

General algorithm

Our computational approach is based on previously reported frameworks to describe AMPA receptor trafficking at synapses [1] and actin retrograde flow in growth cones [2]. However, whereas in previous programs the simulations were run one by one, and later visualized using a commercial image analysis software (Metamorph, Molecular Devices), FluoSim is a stand-alone program that allows the fast calculation of thousands of single molecule positions and intensities in parallel, thereby compatible with live image rendering. A further important improvement over previous approaches is that the working space is now determined from an imported microscopy image with potentially complex shapes. The cell outline is imported as a region file previously made in Metamorph or Image J, or directly drawn on the screen using a toolbox. This internal space is randomly populated by a given number of molecules (1-

150,000), GFP-Nrx1β in our case. Those molecules are kept within the cell boundaries by rebound conditions. An individual molecule is characterized by its 2D coordinates x and y over time t, and its intensity. The total duration of the simulations (typically 5 s - 10 min) is set according to the experiment to model. The time step of the simulations Δt is varied between 1-100 ms, corresponding to typical detector frame rates in FCS and SPT experiments, respectively. The initial position of a freely diffusing molecule is defined by $x(0) = x_0$ and $y(0) = y_0$, taken as random numbers to fall within the cell boundaries. The diffusion coefficient outside the contact area (D_{out}) is chosen around 0.3 µm²/s, based on SPT data, while the contact area can be characterized by a lower diffusion coefficient (D_{in} in the range of 0.1-0.3 µm²/s), owing to molecular crowding and steric hindrance. An additional coefficient called crossing probability ($P_{crossing}$ between 0 and 1) describes the potentially limited penetrability of molecules into the contact. A small fraction of immobile Nrx1β molecules was observed (~5% with $D < 10^{-3}$ µm²/s), which might be due to non-specific adhesion or endocytosis[3], and introduced in the program at random positions with zero diffusion coefficient. In the contact area, surface-diffusing Nrx1β and Nlg1 molecules are allowed to bind reversibly, with first order binding and unbinding rates k_{on} and k_{off}, respectively (both in s^{-1}). The k_{off} value was taken from surface plasmon resonance data obtained on purified extracellular domains of Nrx1β and Nlg1 [4] (0.015 s^{-1}), while k_{on} was inferred from previous experiments of Nrx1β-coated Quantum dots interacting with neurons expressing Nlg1 [5] (0.15 s^{-1}). Bound complexes were allowed to diffuse at a lower diffusion coefficient D_{trap} = 0.04 µm²/s, reflecting their slow movement within the cell-cell contact. The number of Nlg1-mCherry binding sites is assumed to be in excess (consistently with high expression levels in COS-7 cells), such that the binding rate k_{on} is maintained constant throughout the simulations, i.e. it does not depend on the number of Nrx1β-Nlg1 complexes formed over time. We further consider a uniform (i.e. not discrete) distribution of binding sites in the trapping area, also consistent with a high density of Nlg1 molecules.

Calculation of positions

At each time step, the (x,y) coordinates of each molecule are incremented by the distances (Δx, Δy), which depend on whether the molecule is outside or inside the contact area, or in an adhesive complex. If the molecule is outside the contact area, it follows a random walk with diffusion coefficient D_{out}. The positions x(t) and y(t) are then incremented at each time step by $n_x(2D\Delta t)^{1/2}$ and $n_y(2D\Delta t)^{1/2}$, respectively, where n_x and n_y are random numbers generated from a normal distribution with 0 mean and variance unity, to account for the stochastic nature of diffusion. This ensures that the mean square displacement stays proportional to time, i.e. $<x^2 + y^2> = 4D_{out}t$. If the adhesion molecule reaches a contact area, it is set to diffuse with a lower diffusion coefficient D_{in}, with increments $n_x(2D_{in}\Delta t)^{1/2}$ and $n_y(2D_{in}\Delta t)^{1/2}$. Whenever the molecule resides in the contact area, it is allowed to bind to its counter-receptor only if the probability of coupling in this time interval, $P_{coupl} = k_{on}\Delta t$**,** is greater than a random number N between 0 and 1 generated from a uniform distribution. If this is not the case, the molecule continues to diffuse until both conditions are met, i.e. the

molecule remains in the contact area and the probability of binding is greater than the random number N, chosen different at each time increment. Upon binding, the adhesive complex is set to diffuse with a slow diffusion coefficient D_{trap}, thus the positions x(t) and y(t) are incremented by $n_x(2D_{trap}\Delta t)^{1/2}$ and $n_y(2D_{trap}\Delta t)^{1/2}$, respectively. The complex stays bound until the probability for dissociation $P_{detach} = k_{off}\Delta t$, exceeds another random number N'. It then binds again or escapes into the contact or outside space. Starting with random positions, it can take a relatively long time before molecules reach a steady-state distribution. Yet, it is necessary that the molecular system is at steady-state before recording a given simulation. To accelerate this process, an option is proposed in FluoSim to theoretically estimate the steady-state, by placing more molecules in the membrane compartments, considering both slower diffusion and adhesion. The molecular enrichment was then given by the formula ($P_{crossing}$ D_{out}/D_{in}) $(1+k_{on}/k_{off})$. Using those dynamic coefficients, we chose the value of $P_{crossing}$ for each type of simulation (0.25-0.7), to match the experimental enrichment of GFP-Nrx1β in the cell contact normalized to outside areas.

Molecule size, intensity, and photophysics

In addition to its position, each molecule is defined by its size and fluorescence intensity over time. Single molecules are represented either by a discrete point of intensity 1, or by a Gaussian intensity profile with a peak value directly coded on a 16-bit grey scale (0-65535 levels), or expressed in photons/sec associated with a conversion rate, or gain, which gives the number of grey levels read on the virtual camera chip per incoming photon. The Gaussian representation comprises an adjustable width σ in the order of λ/(2 x N.A.), where λ is the emission wavelength of the fluorophore, and N.A. is the numerical aperture of the objective (1.49 in our set-up). The corresponding FWHM is then equal to 2σ √(2.ln2)[6]. In our experiments, we used EGFP-Nrx1β: σ_{GFP} = 510/(2 x 1.49) = 171 nm, and $FWHM_{GFP}$ = 470 nm, and Atto647N-conjugated Nanobody: $\sigma_{Atto647N}$ = 670/(2 x 1.49) = 225 nm and $FWHM_{Atto647N}$ = 529 nm.

Transitions between ON/OFF intensity values are set by two photo-physical parameters: the switch-on rate (k_{on}^{Fluo}) and the switch-off rate (k_{off}^{Fluo}). These rates are in units of sec^{-1} and represent the probabilities per unit of time that a molecule will switch from a state where it emits fluorescence, to a state where it does not emit fluorescence, and vice versa. The rates are specific for each fluorophore (GFP, PAGFP, mCherry, mEos2, Atto dyes) and strongly depend on the laser powers used to image them. By playing on these two rates, many types of experiments can be mimicked. For example, to model a PALM or STORM experiment, one sets a low switch-on rate to induce sparse stochastic emission and a high switch-off rate to induce rapid extinction of fluorescence. In a PAINT experiment, k_{on}^{Fluo} represents instead the rate of binding of fluorescent ligands in solution to receptors on the cell surface, which spontaneously appear in the oblique illumination plane, whereas k_{off}^{Fluo} combines fluorophore photo-bleaching and probe detachment from the cell surface. To mimic a FRAP experiment, k_{off}^{Fluo} is set to a high level in a given ROI to quickly and irreversibly photo-bleach

fluorophores, then monitor recovery. Conversely, in a Photo-Activation of Fluorescence (PAF) experiment, k_{on}^{Fluo} is set to a high value in the ROI to be activated, and the fluorescence redistribution is followed. In FCS experiments, a low value of k_{off}^{Fluo} can be introduced to reproduce observational photo-bleaching.

Running simulations and data export

Once realistic parameters have been tested in live mode, simulations can be generated, and results are exported in various forms. For SPT simulations, .trc files containing the spatial positions and intensity of each molecule over time are saved, and can be loaded later for offline visualization and analysis (menu SPT Analysis). For FRAP and PAF simulations, average intensities over time in defined ROIs are saved as txt files. For FCS simulations, both intensity fluctuations and autocorrelation values over time are exported as .txt files. For SRI simulations, a single super-resolved image integrating all single molecule localizations is exported as a TIFF file. An option is also given to export simulated data as multi-TIFF image stacks. In this mode, single molecule intensity values can be super-imposed to various sources of noise, including a Poisson shot noise applied to the signal and background to mimic the stochastic photon emission of the fluorophores, as well as a Gaussian readout noise applied to a camera offset. By adjusting those various parameters, one can generate images with realistic signal-to-noise rendering.

List of simulations performed

SPT simulations

To mimic the sparse density of GFP-Nrx1β bound to Nanobody-Atto647N as used in uPAINT experiments, a low number of molecules were introduced in the model cell (250 molecules corresponding to a surface density of 0.43 mol/µm²). The length of the simulated trajectories was adjusted to the experimental one by choosing k_{off}^{Fluo} = 3 s^{-1} corresponding to a mean trajectory duration of 340 ms (i.e. 17 frames of 20 ms each). The parameter k_{on}^{Fluo} which determines the number of fluorescent molecules was set to 1 s^{-1}, so as to yield approximately the same density of visible molecules per surface area as in the experiments (0.1 mol/µm²). Sequences of 4,000 frames were generated as in the experiments, and only trajectories longer than 20 frames were selected (total 2244 trajectories). The diffusion coefficient, *D*, was calculated for each trajectory, from linear fits of the first 4 points of the MSD function versus time. Five independent simulations were run for each set of parameters, allowing the construction of histograms of diffusion coefficients directly comparable to SPT experiments.

FRAP simulations

To match the very dense distribution of GFP-Nrx1β molecules that characterize FRAP experiments, a large number of molecules was introduced in the virtual cell (150'000

molecules corresponding to a surface density of ~200 mol/µm²). Simulations of 1,000 frames, including a baseline of 100 frames, were generated with a time step of 100 ms (total duration 100 s). Two areas were recorded, one within the adhesive contact, the other outside (bleached diameter = 2.8 µm). The photo-activation rate was set to a maximal value (k_{on}^{Fluo} = 5 s^{-1}), i.e. all molecules are initially fluorescent, while the photo-bleaching rate is set to zero during baseline and recovery acquisition (i.e. observational photo-bleaching is neglected here). During the short photo-bleaching period (400 ms), the photo-bleaching rate is set to k_{off}^{Bleach} = 4.25 s^{-1} for 4 frames, to precisely match the initial drop of fluorescence observed experimentally (~75%). The number of molecules in the photo-bleached areas was computed over time, and normalized between 1 (baseline number of fluorescent molecules before photo-bleaching) and zero (number of fluorescent molecules right after photo-bleaching). FRAP simulations were repeated 30 times, and the corresponding curves were averaged.

PAF simulations

Although initially non-fluorescent, PAGFP-Nrx1β molecules are likely expressed at the same levels as GFP-Nrx1β (since the two constructs bear the same promoter), thus a large number of molecules was also introduced in the virtual cell (respectively 150'000 or 72'000 molecules for contact and no contact experiments, both corresponding to a surface density of ~40 mol/µm²). Simulations of 1,200 frames, including a baseline of 25 frames, were generated with a time step of 100 ms (total duration 120 s). Two areas were recorded, one within the adhesive contact, the other outside (photo-activated diameter = 2.8 µm). Under the LIVE menu, the photo-activation rate k_{on}^{Fluo} is set to zero while the photo-bleaching rate k_{off}^{Fluo} is tuned to its maximal value (10 s^{-1}), until all molecules are non-fluorescent. Then k_{off}^{Fluo} is set back to 0 during baseline and recovery acquisition (i.e. observational photo-bleaching is neglected). An offset of 500 grey levels with a Gaussian noise of 10 grey levels was chosen to match the cell autofluorescence level before photo-activation. During the short photo-activation period (300 ms), the photo-activation rate is set to k_{on}^{Fluo} = 1 s^{-1} for 3 frames, so as to match the initial increase of fluorescence observed experimentally (x 3 for no-contact areas and or x 10 for contact regions). The results of the simulations were exported as multi-TIFF image stacks, that were subsequently opened and analyzed in Metamorph. The average intensity in the photo-activated areas was computed over time, and normalized between 0 (background before photo-activation) and 1 (number of fluorescent molecules right after photo-activation). PAF simulations were repeated 20 times, and the corresponding curves were averaged.

FCS simulations

To mimic the intermediate densities of GFP-Nrx1β bound to Nanobody-Atto647N used in FCS experiments, 200 or 2500 molecules (corresponding to 0.27 and 2.8 mol/µm²) were entered in the program, to simulate cells forming or not forming adhesive contacts, respectively. These values roughly correspond to the experimental labeling densities, and ensure large enough fluctuations to calculate a reliable autocorrelation function. Simulations of 500,000

or 1,000,000 frames with time steps of 5 ms were generated for the two conditions, respectively. The diffraction-limited laser spot is defined by a normalized Gaussian intensity profile with a full width at half maximum (FWHM) of 0.6 µm, and maximal value of 1. A molecule which reaches the ROI containing the virtual laser spot, is counted with an intensity equal to the value of this Gaussian function, at the location *r* from the center of the laser spot. The resulting intensity fluctuations over time were analyzed by computing the autocorrelation function. To mimic the impact of photo-bleaching in the probing area, we introduced in the simulations a small photo-bleaching rate, proportional to the local laser intensity. Hence, the photo-bleaching rate also follows a Gaussian distribution with a maximal rate k_{off}^{Bleach} at the beam center (k_{off}^{Bleach} = 0.4 s^{-1}, 10 times less than for FRAP). Photo-bleaching had more impact on the autocorrelation function when calculated for the slower molecules in the adhesive contact. FCS simulations were repeated 10 times, and the corresponding autocorrelation functions normalized to their initial value, were averaged.

SRI simulations

To mimic STORM experiments that rely on the dense labeling of GFP-Nrx1β bound to Nanobody-Alexa647, a large number of molecules were introduced in the virtual cell (70,000 corresponding to 125 mol/µm²). After the diffusion/trapping steady-state has been reached or imposed, the simulation is paused and all diffusion coefficients are set to zero to mimic cell fixation. This procedure accelerates the calculator which does not have to compute new positions at each time frame and just updates fluorescence states. Alternatively, to take into account the fact that a fraction of transmembrane molecules may still be mobile even after fixation with aldehydes[7], one can impose slow diffusion coefficients. The switch-on rate k_{on}^{Fluo} at which fluorescent dyes spontaneously emit light was determined by measuring the fluorescence intensity collected from single Alexa647-conjugated GFP Nanobody molecules bound to the glass coverslip during a STORM sequence, and counting the number of peaks (mean ± sem = 2.5 ± 0.3 peaks over a time period of 420 sec, n = 20 molecules analyzed, giving k_{on}^{Fluo} = 0.006 s^{-1}). The switch-off rate k_{off}^{Fluo} was determined by taking the inverse of the number of time frames during which single Alexa647-conjugated GFP Nanobodies emitted light before entering again the non-emitting state (5.4 ± 0.5 frames of 20 ms, 87 events analyzed), giving a value of k_{off}^{Fluo} = 9.3 s^{-1}. The on-off duty cycle $\delta = k_{on}^{Fluo}/(k_{on}^{Fluo} + k_{off}^{Fluo})$ is the fraction of time that fluorophores spend in the light-emitting state, and equals here 0.00064, very close to reported values for single Alexa647 dyes in MEA-based STORM buffer [8]. The number of detected molecules per plane in the field of view was around N = 45, corresponding to a total number N/δ = 70,300 actual molecules in the cell geometry that was imaged. Then, simulations were run for 80,000 frames of 20 ms each (total time of 1,600 sec), and a single 16-bit image was generated which contained the integration of all molecule localizations throughout time. To generate a higher number of detected molecules for CNN applications, the parameter k_{on}^{Fluo} was multiplied by a factor of 5 (to 0.03 s^{-1}) to mimic the increase in fluorescence emission induced by the 405 nm laser. Three parameters are used to render the super resolution image: the intensity associated with a single detection; the zoom

factor which is the ratio between the pixel sizes of the super-resolved image and the low resolution reference picture (a 5-fold zoom corresponds to a pixel size of 32 nm in the high resolution image); and the localization precision, which corresponds to the standard deviation of the Gaussian distribution used to spread detections around the theoretical position of the molecule (σ = 25 nm, FWHM = 58 nm). The SRI still image is saved as a TIFF file.

Use of FluoSim to train deep CNNs for fluorescence image reconstruction

Deep-STORM training procedure

To assess the ability of FluoSim to train deep learning algorithms, we used Deep-STORM [9], a Convolutional Neural Network (CNN) designed to localize the positions of fluorescence emitters from dense labeling microscopy images to produce super-resolved images. Deep-STORM is trained with several thousand image pairs: a low resolution fluorescence picture and its associated super-resolved image. The ImageJ plugin ThunderSTORM [10] was previously used to generate hundreds of simulated images (of size 64 x 64 pixels) containing randomly positioned emitters, together with a text file containing their positions [9]. These simulated images depend on several parameters such as camera specifications, point spread function (PSF) of a single emitter, signal-to-noise ratio, and density of emitters. The images and the localization files together with a scaling factor were processed in the MATLAB® (MathWorks) script provided by the authors (*GenerateTrainingExamples.m*) to format and expand the training set, resulting in several thousand pairs of low- and high-resolution images. Each pair contains a randomly selected cropped image of 26x26 pixels and its high resolution counterpart scaled by a zoom factor (typically 4 or 8). These pairs, exported in a single file, are given to the Python script (*Training.py*) to train the network, which provides as output two files containing the network weights and the mean and standard deviation of each image of the training data set, respectively. The weights can ultimately be provided to the testing Python script (*Testing.py*) to create the Deep-STORM network used to reconstruct super-resolution images. Both Python and MATLAB scripts are freely accessible from the Deep-STORM project web page: https://github.com/EliasNehme/Deep-STORM. Deep-STORM and its dependencies were installed on a 64 bits Ubuntu (18.04.2 LTS) workstation, equipped with an Intel Xeon CPU (E5-1607 @ 3.00 GHz x4) together with 40 GB RAM and a 24 GB memory Nvidia P6000 graphics card.

Reconstruction of simulated microtubules

To validate the procedure, we first trained a Deep-STORM network using ThunderSTORM, with simulated microtubule datasets that were generated for a contest to evaluate software packages for single molecule localization microscopy [11], and are available on the EPFL website: http://bigwww.epfl.ch/smlm/challenge2013/index.html. We generated with ThunderSTORM 200 images of 64 x 64 pixels containing randomly positioned emitters (density = 0.5 μm^{-2}). Each emitter was set to produce a Gaussian diffraction spot in the simulated images, with a

standard deviation ranging between 115 nm and 180 nm and a peak intensity ranging from 110 to 440 grey levels. No intensity offset was added but a Poisson noise was applied to each pixel. A zoom factor of 8 was set in the MALTAB script to expand the training dataset producing the pairs of low/high-resolution images used to train the Deep-STORM network. This network was able to reconstruct a high resolution image of the simulated microtubules, as described [9].

Another Deep-STORM network was then trained with simulated data generated by FluoSim. A square region of 64 x 64 pixels with a 100 nm pixel size was defined, and populated with 20 emitters to reach an emitter density of ~0.5 μm^{-2}. To randomize the positions of the emitters between consecutive frames, the diffusion coefficient of the emitters (D = 2 $\mu m^2/s$) and the simulation time step (δt = 1 s) were chosen so that the displacements were of the same magnitude as the region length, i.e. $\sqrt{4D\delta t} \approx 6.4$ µm. Each emitter was set to produce a Gaussian PSF of 120 nm standard deviation and of peak intensity equal to 110 grey levels in the recorded frames. Here, no intensity offset was added and no Poisson noise was applied to the simulated images. An SPT simulation of 200 steps was performed with the Stack Export option enabled. The resulting image stack and localization file were then processed by the MATLAB script to produce 5,000 pairs of low/high resolution images (Zoom Factor = 8), which were used to train the Deep-STORM network. The resulting network was tested on the simulated microtubules and compared to the ground truth and the image reconstructed with the ThunderSTORM-based network.

Reconstruction of real microtubules and Nrx1β-Nlg1 cell-cell contact

To reconstruct our own STORM experiments using Deep-STORM networks, we had to generate specific training sets which reproduce our experimental conditions. Two networks were educated, one trained with ThunderSTORM and one with FluoSim. In both cases 200 square images of 64x64 pixels at pixel size = 160 nm were produced. Each frame contained 20 emitters resulting in an emitter density of 0.5 μm^{-2}; for the FluoSim simulation, we followed the same procedure to randomize the positions as for the simulated microtubules. Each emitter was set to produce a Gaussian PSF with standard deviation ranging from 170 nm to 212 nm in the ThunderSTORM simulation, and with a standard deviation equal to 192 nm in FluoSim. In both situations, the PSF maximum was set to 110 grey levels, an intensity offset equal to 30 was added to the images, and a Poisson noise was applied so that each resulting pixel value was randomly taken in a Poisson probability distribution of mean (and hence variance) equal to the sum of the raw pixel intensity value and the intensity offset. The generated low resolution image stacks and localization files were processed by the MATLAB script to produce 5000 pairs of low/high resolution images (Zoom Factor = 4). The Zoom Factor was set to 4 to limit the amount of GPU memory needed to reconstruct the large STORM experiment images (118 x 284 pixels for the Real MT and 168x288 pixels for the Cell Cell contact). Two Deep-STORM networks were finally trained using the data originating from both ThunderSTORM and FluoSim simulations, and were used to reconstruct real microtubules and the Nrx1β-Nlg1 cell-cell contact from STORM experiments in both low and

high emitters density situations. STORM experiments in the low density regime resulted in a large amount of images (~50,000) which could not be processed directly with the provided Python script, because of saturation in computer memory. To overcome this problem, the STORM images were reconstructed by batches of 200 images, each batch giving a single super-resolved image. Once reconstructed, the single super-resolved images were summed up to produce the final STORM image.

Supplementary references

1. Czöndör, K. *et al.* Unified quantitative model of AMPA receptor trafficking at synapses. *Proc. Natl. Acad. Sci. U. S. A.* **109**, 3522–7 (2012).

2. Garcia, M. *et al.* Two-tiered coupling between flowing actin and immobilized N -cadherin/catenin complexes in neuronal growth cones. *Proc. Natl. Acad. Sci.* **112**, 6997–7002 (2015).

3. Savas, J. N. *et al.* The Sorting Receptor SorCS1 Regulates Trafficking of Neurexin and AMPA Receptors. *Neuron* **87**, 764–780 (2015).

4. Comoletti, D. *et al.* Characterization of the Interaction of a Recombinant Soluble Neuroligin-1 with Neurexin-1β. *J. Biol. Chem.* **278**, 50497–50505 (2003).

5. Saint-Michel, E., Giannone, G., Choquet, D. & Thoumine, O. Neurexin/neuroligin interaction kinetics characterized by counting single cell-surface attached quantum dots. *Biophys. J.* **97**, 480–9 (2009).

6. Deschout, H. *et al.* Precisely and accurately localizing single emitters in fluorescence microscopy. *Nat. Methods* **11**, 253–266 (2014).

7. Tanaka, K. A. K. *et al.* Membrane molecules mobile even after chemical fixation. *Nat. Methods* **7**, 865–866 (2010).

8. Dempsey, G. T., Vaughan, J. C., Chen, K. H., Bates, M. & Zhuang, X. Evaluation of fluorophores for optimal performance in localization-based super-resolution imaging. *Nat. Methods* **8**, 1027–1040 (2011).

9. Nehme, E., Weiss, L., Michaeli, T. & Shechtman, Y. Deep-STORM: super-resolution single-molecule microscopy by deep learning. *Optica* **18**, 2334–2536 (2018).

10. Ovesný, M., Křížek, P., Borkovec, J., Švindrych, Z. & Hagen, G. M. ThunderSTORM: A comprehensive ImageJ plug-in for PALM and STORM data analysis and super-resolution imaging. *Bioinformatics* **30**, 2389–2390 (2014).

11. Sage, D. *et al.* Quantitative evaluation of software packages for single-molecule localization microscopy. *Nat. Methods* **12**, 717–24 (2015).

12. Kerr, R. A. *et al.* Fast Monte Carlo simulation methods for biological reaction-diffusion systems in solution and on surfaces. *SIAM J. Sci. Comput.* **30**, 3126 (2008).

13. Tapia, J.-J. *et al.* MCell-R: A Particle-Resolution Network-Free Spatial Modeling Framework. *Methods Mol. Biol.* **1945**, 203–229 (2019).

14. Tolle, D. P. & Le Novère, N. Meredys, a multi-compartment reaction-diffusion simulator using multistate realistic molecular complexes. *BMC Syst. Biol.* **4**, 24 (2010).

15. Ibrahim, B. *et al.* Spatial Rule-Based Modeling: A Method and Its Application to the Human Mitotic Kinetochore. *Cells* **2**, 506–544 (2013).

16. Angiolini, J., Plachta, N., Mocskos, E. & Levi, V. Exploring the Dynamics of Cell Processes through Simulations of Fluorescence Microscopy Experiments. *Biophys. J.* **108**, 2613–2618 (2015).

17. Bläßle, A. *et al.* Quantitative diffusion measurements using the open-source software PyFRAP. *Nat. Commun.* **9**, 1–14 (2018).

18. Venkataramani, V., Herrmannsdörfer, F., Heilemann, M. & Kuner, T. SuReSim: Simulating localization microscopy experiments from ground truth models. *Nat. Methods* **13**, 319–321 (2016).

Supplementary Table 1. Comparison of FluoSim with other packages

Software	**2D/3D**	**Modalities**	**Real-time**	**Fluorescence**	**Reference**
FluoSim	2D	SPT, FRAP, PAF, FCS, PALM, STORM, uPAINT	Yes	Yes	This study
MCell	3D	Diffusion, multi-state reactions	No	No	[12,13]
Meredys	3D	Diffusion, multi-state reactions	No	No	[14]
SRSim	3D	Diffusion, multi-state reactions	No	No	[15]
FERNET	3D	FCS	No	Yes	[16]
pyFRAP	3D	FRAP	No	Yes	[17]
SuReSim	3D	SRI	No	Yes	[18]

Table Footnotes

Only particle-based software including spatial information is cited here.

Supplementary Figures 1-5

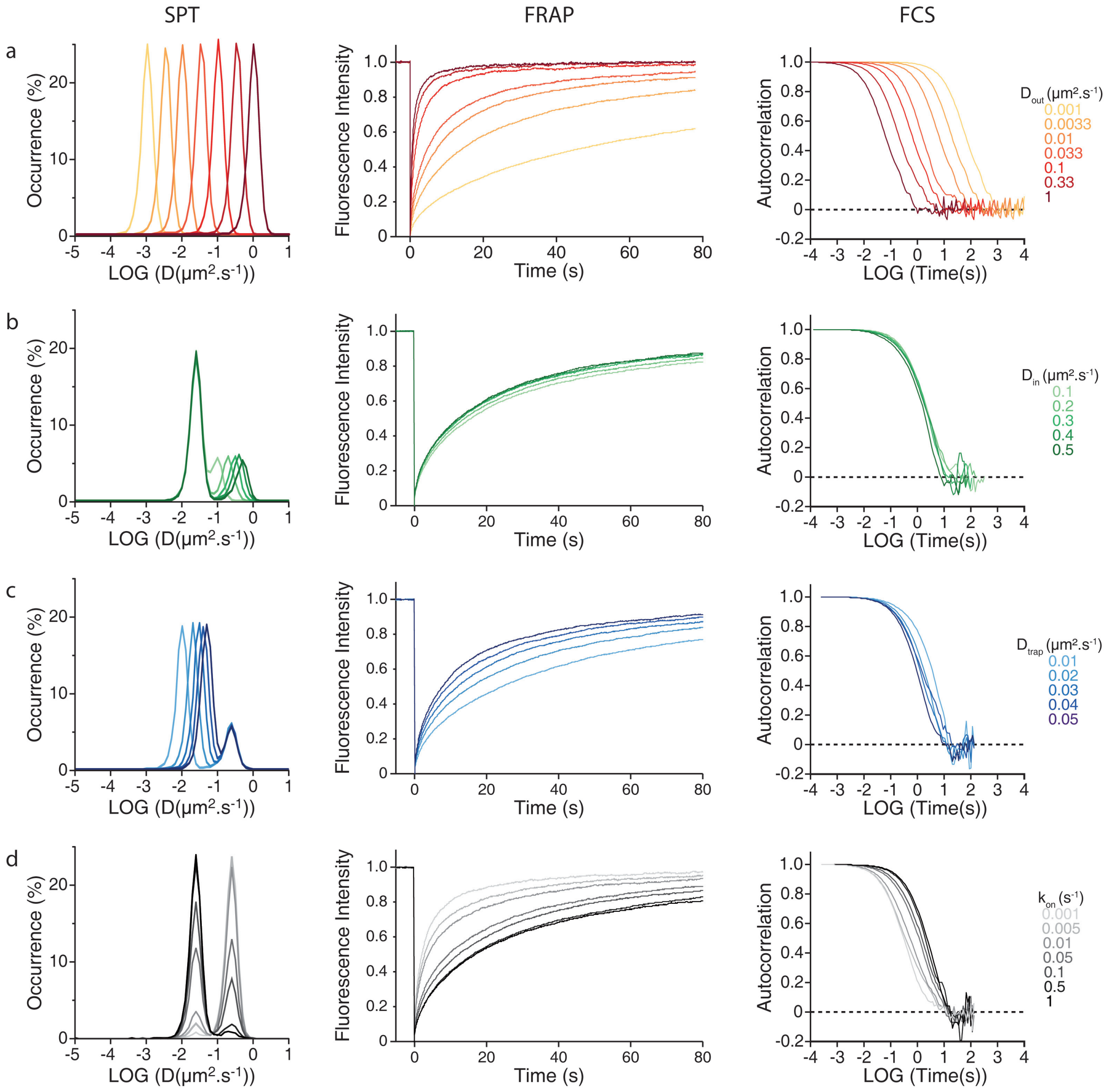

Figure S1. Effect of model parameters on SPT, FRAP and FCS simulations.

(**a**) Effect of varying D_{out} (from 0.001 to 1 µm².s^{-1}) on SPT, FRAP and FCS simulations outside the adhesive contact. Note the right shift of the distribution of diffusion coefficients in SPT, the increase in fluorescence recovery for FRAP, and the left shift of the autocorrelation function in FCS, as D_{out} increases. **(b**) Effect of varying D_{in} (from 0.1 to 0.5 µm².s^{-1}) on the SPT, FRAP and FCS simulations inside the adhesive contact, the other parameters being held constant (D_{out} = 0.25 µm².s^{-1}, D_{trap} = 0.025 µm².s^{-1}, k_{on} = 0.15 s^{-1}, k_{off} = 0.015 s^{-1}). Note the modest effect of D_{in} on the FRAP and FCS types of curves, which is due to the fact that only a small proportion of molecules diffuse freely at D_{in} within the contact (i.e. most molecules are trapped, due to the high ratio k_{on}/k_{off} = 10). In SPT, the small peak of diffusing molecules gradually shifts to the left as D_{in} decreases from D_{out} to D_{trap}. **(c)** Effect of varying D_{trap} (from 0.01 to 0.05 µm².s^{-1}) on SPT, FRAP, and FCS simulations inside the adhesive contact (D_{out} = D_{in} = 0.25 µm².s^{-1}, k_{on} = 0.15 s^{-1}, k_{off} = 0.015 s^{-1}). Note the left shift of the distribution of diffusion coefficients in SPT, the decrease in fluorescence recovery for FRAP, and the right shift of the autocorrelation function in FCS, as D_{trap} decreases. **(d)** Effect of varying k_{on} on SPT, FRAP, and FCS simulations inside the adhesive contact (D_{out} = D_{in} = 0.25 µm².s^{-1}, D_{trap} = 0.025 µm².s^{-1}, k_{off} = 0.15 s^{-1}). Note that in SPT the slowly moving population centered at D_{trap} increases with k_{on}, while the highly mobile population centered at D_{out} decreases concomitantly. Increasing k_{on} also slows down the fluorescence recovery in FRAP, and induces a right shift of the FCS curve.

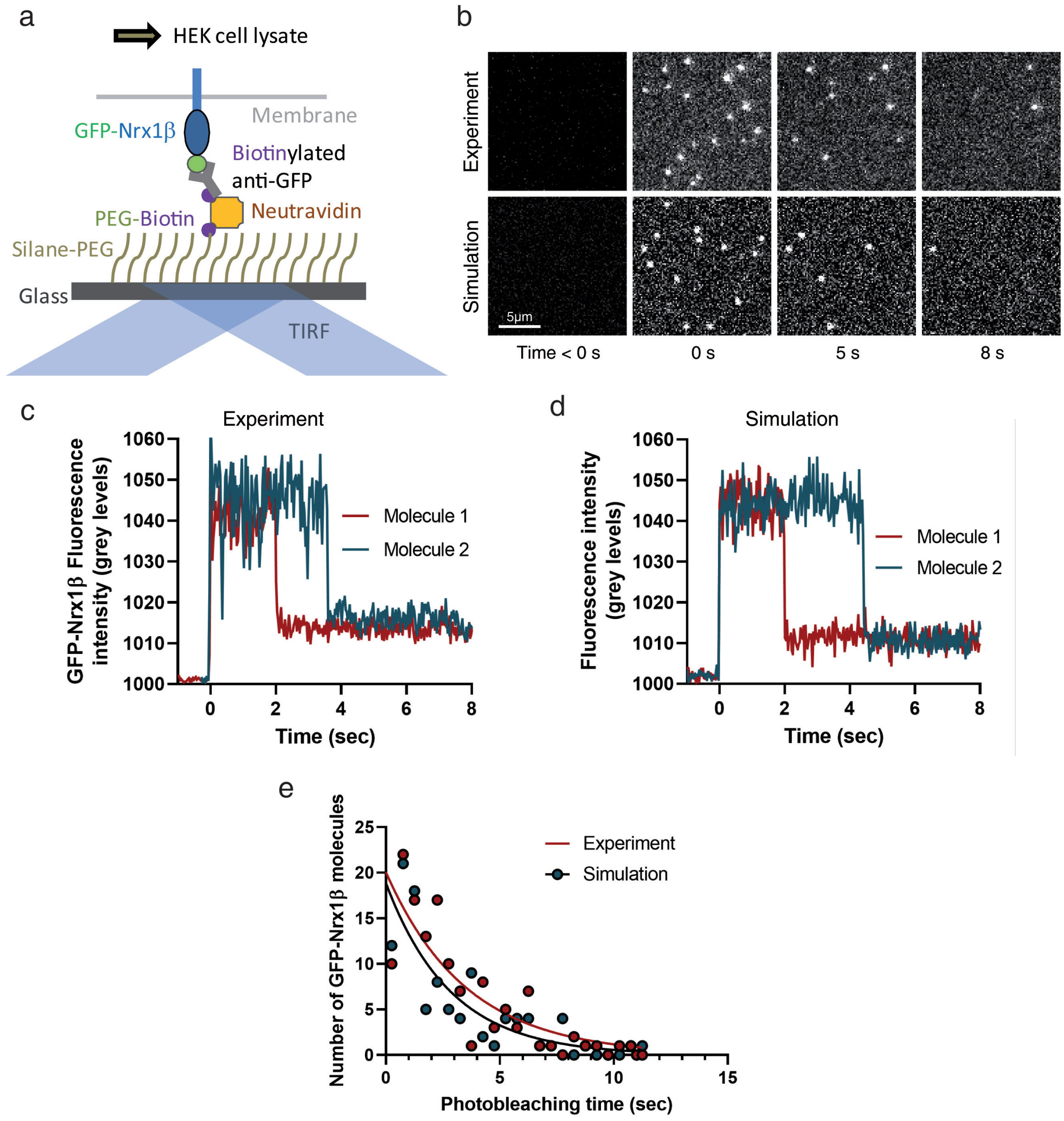
a
HEK cell lysate
Membrane
GFP-Nrx1β
Biotinylated anti-GFP
Neutravidin
PEG-Biotin
Silane-PEG
Glass
TIRF
b
Experiment
Simulation
5μm
Time < 0 s
0 s
5 s
8 s
c
Experiment
GFP-Nrx1β Fluorescence intensity (grey levels)
Molecule 1
Molecule 2
Time (sec)
d
Simulation
Fluorescence intensity (grey levels)
Molecule 1
Molecule 2
Time (sec)
e
Number of GFP-Nrx1β molecules
Experiment
Simulation
Photobleaching time (sec)

Figure S2. Introduction of noise in images generated by FluoSim and comparison to single molecule pull-down data.

(a) Schematic of the single molecule pull-down experiment. Individual GFP-Nrx1β molecules expressed in HEK cells are immobilized on glass substrates and imaged by TIRF microscopy. **(b)** Representative time lapse sequences of the experiment, and corresponding simulations. The 488-nm laser is turned on at time 0, resulting in progressive photobleaching of the GFP molecules within 10 sec. Before the laser is turned on, the only signal comes from the camera offset and noise. **(c)** Graph showing the fluorescence intensity over time of two representative GFP-Nrx1β molecules, which photobleach at different times. Most molecules photobleached in one step, consistent with the fact that Nrx1β is a monomer. **(d)** Graph showing the simulated fluorescence signal over time of two virtual GFP molecules, obtained with the following parameters: peak signal = 350 photons/s; gain = 20; background = 20 photons/s; Poisson noise applied to both signal and background; camera offset = 1000 grey levels, readout noise = 5 grey levels; and k_{off}^{Fluo} = 0.30 sec^{-1}. Note the good agreement in signal and noise levels between experiments and simulations. **(e)** Graph showing the distribution of the photobleaching times calculated for 131 GFP-Nrx1β molecules, and 106 simulated molecules, respectively. The lines are decreasing exponential fits, giving the value of the photobleaching rates k_{off}^{Fluo} = 0.28 sec^{-1} and 0.35 sec^{-1} for experiments and simulations, respectively.

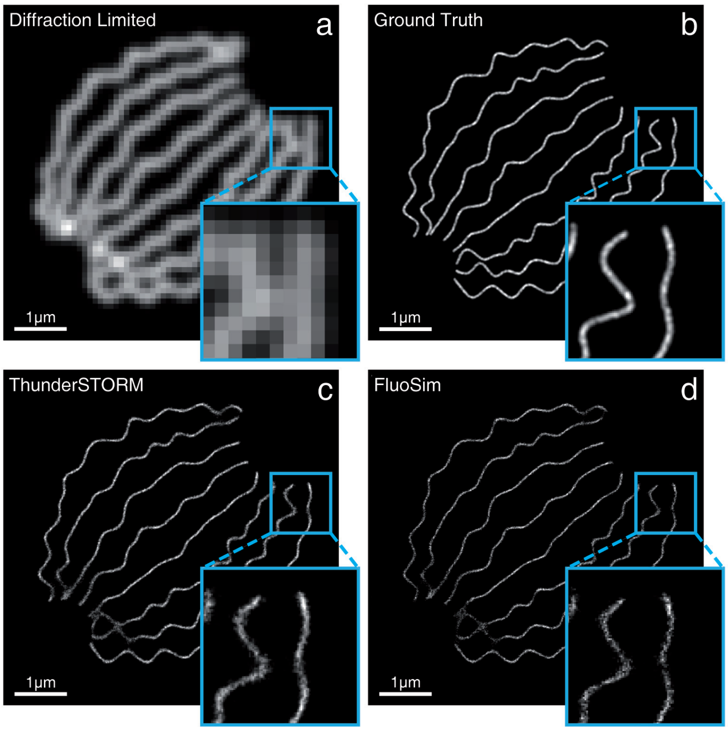

Figure S3. Reconstruction of super-resolved images of simulated microtubules using deep CNN trained with FluoSim.

(a) Maximal intensity projection image of 350 frames of simulated single molecules randomly placed at high density along virtual microtubules. **(b)** Ground truth image of simulated microtubules taken from the EPFL website [11]. **(c)** Image reconstructed by the CNN from the single molecule microtubule stack, after training with ThunderSTORM. **(d)** Image reconstructed by the CNN from the single molecule microtubule stack, after training with FluoSim. Note that the images reconstructed by the CNN are close to the ground truth, using either ThunderSTORM or FluoSim training. Insets show zooms on several microtubule ends to highlight the reconstruction accuracy.

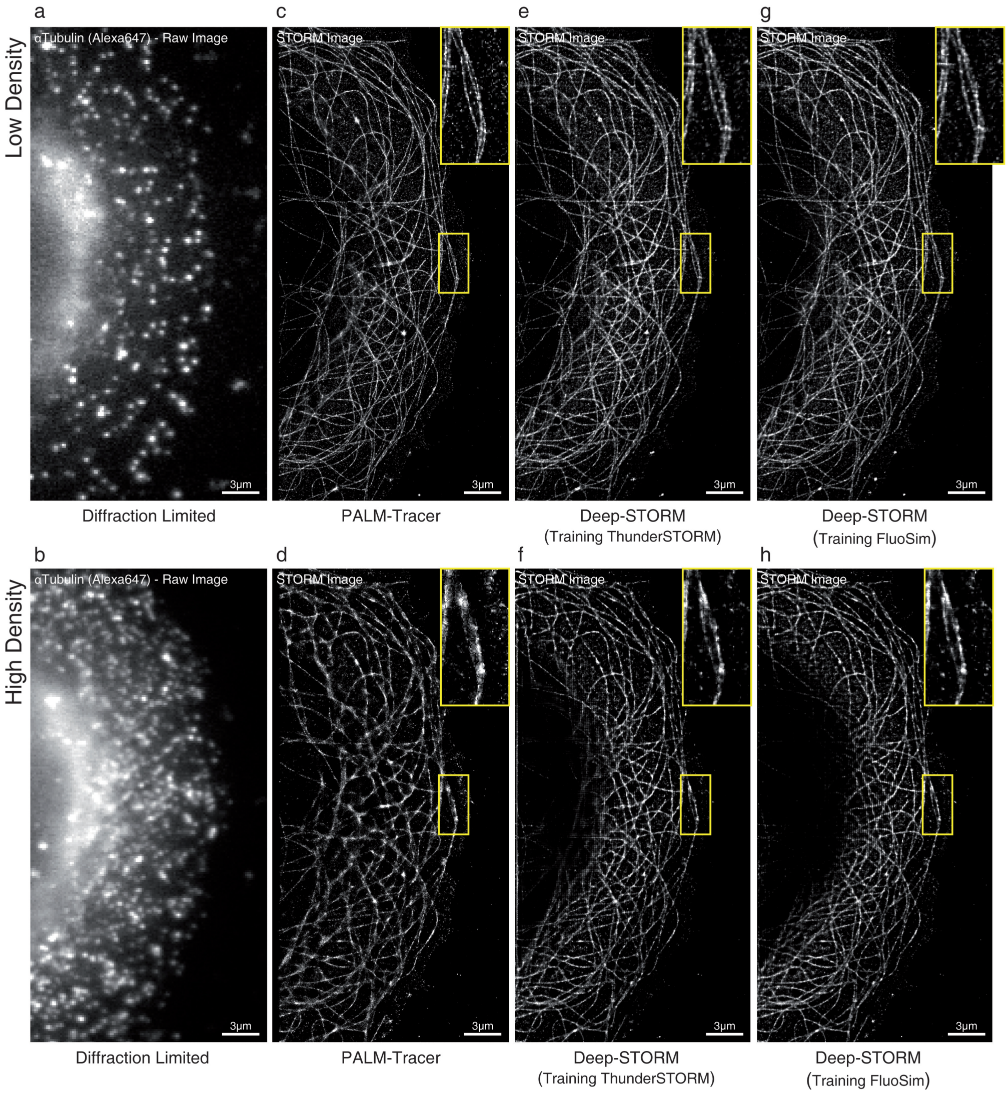

Figure S4. Reconstruction of super-resolved images of real microtubules using deep CNN trained with FluoSim.

(a, b) Single plane images of microtubules in COS-7 cells, labeled with primary α-tubulin antibody followed by Alexa647-conjugated secondary antibody, and acquired under STORM conditions at low or high density, respectively (obtained for two different values of 405 nm laser power). **(c, d)** Images of microtubules reconstructed using PALM-Tracer from a stack of 48,000 frames at low molecule density, or a stack of 4,000 frames at high molecule density, respectively. Note that the image resolution is degraded at high molecule density because single molecules are too close to one another for proper centroid determination. **(e, f)** Images of microtubules reconstructed by the CNN trained with ThunderSTORM, from low and high molecule density stacks, respectively. **(g, h)** Images of microtubules reconstructed by the CNN trained with FluoSim, from low and high molecule density stacks, respectively. Note that the CNN performs well at both low and high molecule density, thereby offering a significant temporal gain in the image acquisition process.

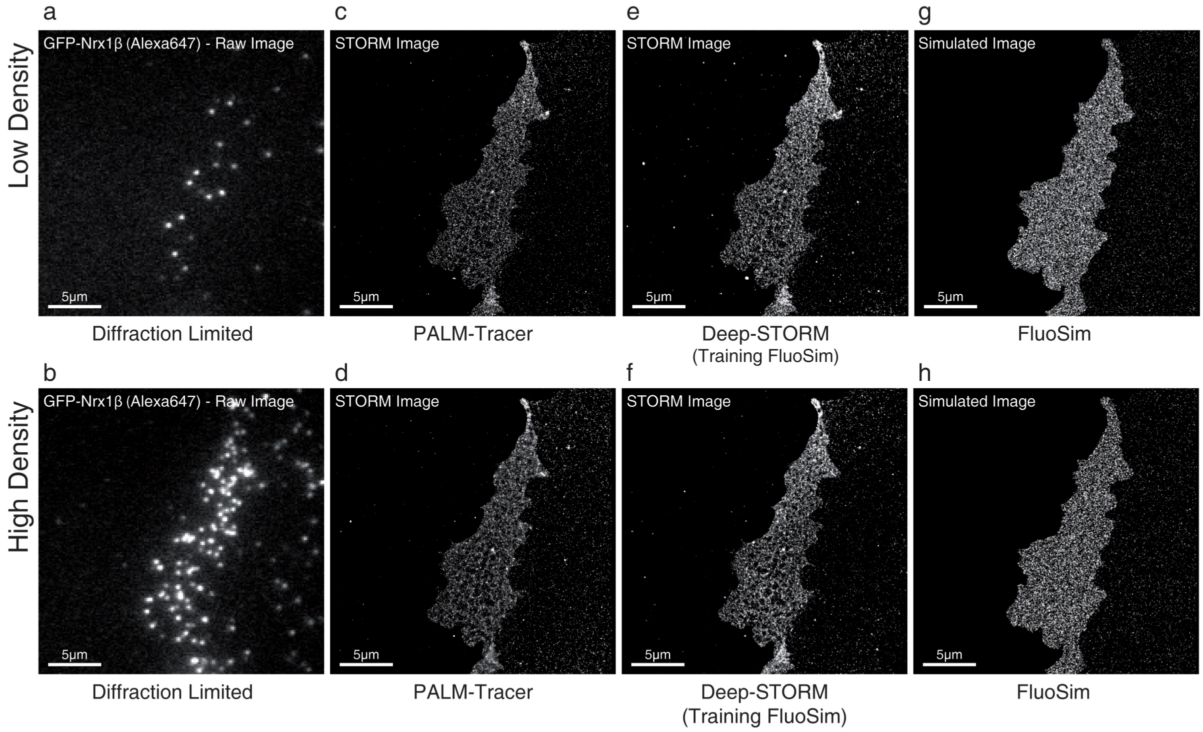

Figure S5. Reconstruction of super-resolved images of Nrx1β-Nlg1 cell contacts using deep CNN trained with FluoSim.

(a, b) Single plane images of GFP-Nrx1β in COS-7 cells labeled with Alexa647-conjugated GFP nanobody and acquired under STORM conditions, at low or high density, respectively. **(c, d)** Images of Nrx1β-Nlg1 adhesive contacts reconstructed using PALM-Tracer from a stack of 48,000 frames at low molecular density (~30 molecules per frame in the field of view), or a stack of 4,000 frames at high molecular density (~150 molecules per frame in the field of view), respectively. **(e, f)** Images of Nrx1β-Nlg1 contacts reconstructed by the CNN trained with FluoSim, from low and high molecule density stacks, respectively. **(g, h)** SRI images were directly generated by FluoSim at low and high molecule density, respectively. 47,000 molecules were entered in the simulator for a cell surface area of 544 µm², with parameters k_{on} = 0.15 s^{-1}, k_{off} = 0.015 s^{-1}, $P_{crossing}$ = 0.81, k_{on}^{Fluo} = 0.006 s^{-1} (low density) or 0.03 s^{-1} (high density), and k_{off}^{Fluo} = 9.3 s^{-1}. The total number of detections at low versus high molecular density was 1,436,260 vs 462,607 for experiments and 1,434,610 vs 615,262 for simulations.
