# Supplementary material for: FluoSim: simulator of single molecule dynamics for fluorescence live-cell and super-resolution imaging of membrane proteins
Source: Sci Rep. 2020 Nov 17;10:19954. doi: 10.1038/s41598-020-75814-y (PMC7672080; doi:10.1038/s41598-020-75814-y)
Supplement: Supplementary file 3 — Supplementary Information 3. [file 41598_2020_75814_MOESM3_ESM.docx]

**Title: FluoSim: simulator of single molecule dynamics for fluorescence live-cell and super-resolution imaging of membrane proteins**

**Authors:** Matthieu Lagardère^1^, Ingrid Chamma^1^, Emmanuel Bouilhol^2^, Macha Nikolski^2^, Olivier Thoumine^1^*

*Corresponding author: [olivier.thoumine@u-bordeaux.fr](mailto:olivier.thoumine@u-bordeaux.fr)

**Affiliations:**

1. Univ. Bordeaux, CNRS, Interdisciplinary Institute for Neuroscience, IINS, UMR 5297, F-33000 Bordeaux, France

2. Univ. Bordeaux, CNRS, IBGC, UMR 5095, F-33000 Bordeaux, France

**List of supplementary material**

**Supplementary text**

General description of Fluosim

List of simulations performed

**Supplementary References**

**Supplementary Table 1.** Comparison of FluoSim with other packages

**Supplementary Figures 1-5**

**Figure S1 and legend**

**Figure S2 and legend**

**Figure S3 and legend**

**Figure S4 and legend**

**Figure S5 and legend**

**Supplementary Software: FluoSim.zip**

The FluoSim software is provided as a compressed .zip file to be installed on a computer equipped with Windows operating system.

**Supplementary File: FluoSim user manual**

The FluoSim User manual describes step-by-step how to use the software. It includes examples of simulations that were used to fit the experimental data contained in the manuscript.

**Supplementary Movie: FluoSim demonstration**

To visualize a high definition movie demonstrating the various imaging modalities of FluoSim, please go to the following website: <http://www.iins.u-bordeaux.fr/SOFTWARE-285?lang=en>
