# Supplementary figures and images for: FluoSim: simulator of single molecule dynamics for fluorescence live-cell and super-resolution imaging of membrane proteins
Source: Sci Rep. 2020 Nov 17;10:19954. doi: 10.1038/s41598-020-75814-y (PMC7672080; doi:10.1038/s41598-020-75814-y)

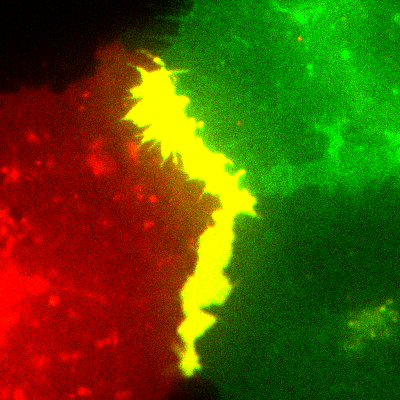

Supplement: Supplementary file 4 — Supplementary Information 4. [file 41598_2020_75814_MOESM4_ESM.zip › FluoSim/CellSamples/GFP-Nrx1b-Nlg1mcherry-merge.bmp]

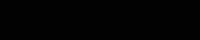

Supplement: Supplementary file 4 — Supplementary Information 4. [file 41598_2020_75814_MOESM4_ESM.zip › FluoSim/CellSamples/LTP.tif]

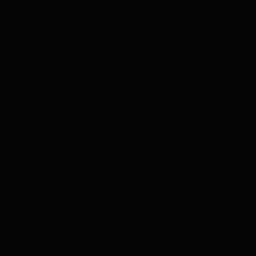

Supplement: Supplementary file 4 — Supplementary Information 4. [file 41598_2020_75814_MOESM4_ESM.zip › FluoSim/CellSamples/Single Pull.tif]

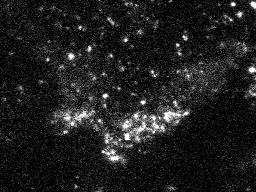

Supplement: Supplementary file 4 — Supplementary Information 4. [file 41598_2020_75814_MOESM4_ESM.zip › FluoSim/Resources/Examples/FCS-in.tif]

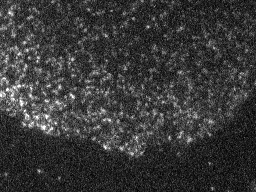

Supplement: Supplementary file 4 — Supplementary Information 4. [file 41598_2020_75814_MOESM4_ESM.zip › FluoSim/Resources/Examples/FCS-out.tif]

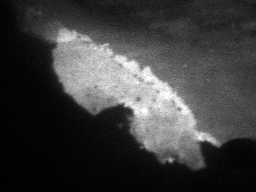

Supplement: Supplementary file 4 — Supplementary Information 4. [file 41598_2020_75814_MOESM4_ESM.zip › FluoSim/Resources/Examples/FRAP-in.tif]

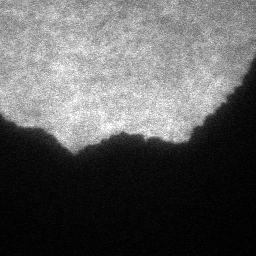

Supplement: Supplementary file 4 — Supplementary Information 4. [file 41598_2020_75814_MOESM4_ESM.zip › FluoSim/Resources/Examples/FRAP-out.tif]

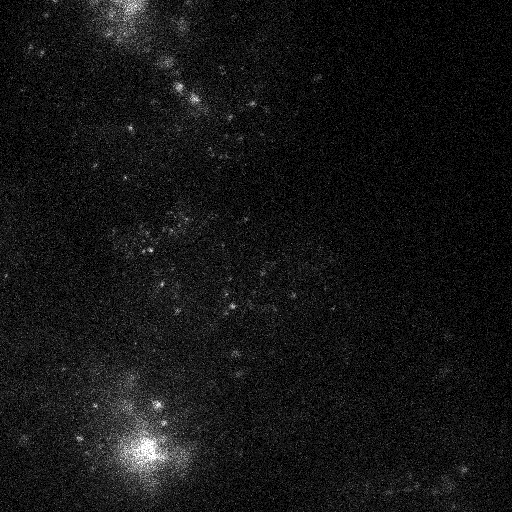

Supplement: Supplementary file 4 — Supplementary Information 4. [file 41598_2020_75814_MOESM4_ESM.zip › FluoSim/Resources/Examples/PAF-in.bmp]

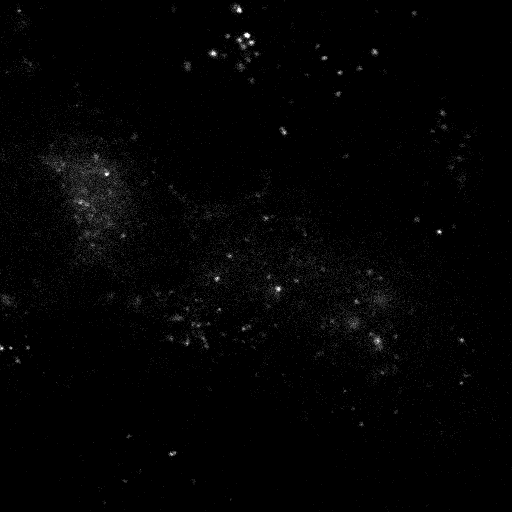

Supplement: Supplementary file 4 — Supplementary Information 4. [file 41598_2020_75814_MOESM4_ESM.zip › FluoSim/Resources/Examples/PAF-out.bmp]

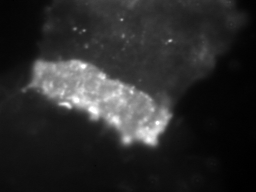

Supplement: Supplementary file 4 — Supplementary Information 4. [file 41598_2020_75814_MOESM4_ESM.zip › FluoSim/Resources/Examples/SPT.tif]

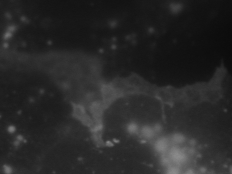

Supplement: Supplementary file 4 — Supplementary Information 4. [file 41598_2020_75814_MOESM4_ESM.zip › FluoSim/Resources/Examples/SRI.tif]

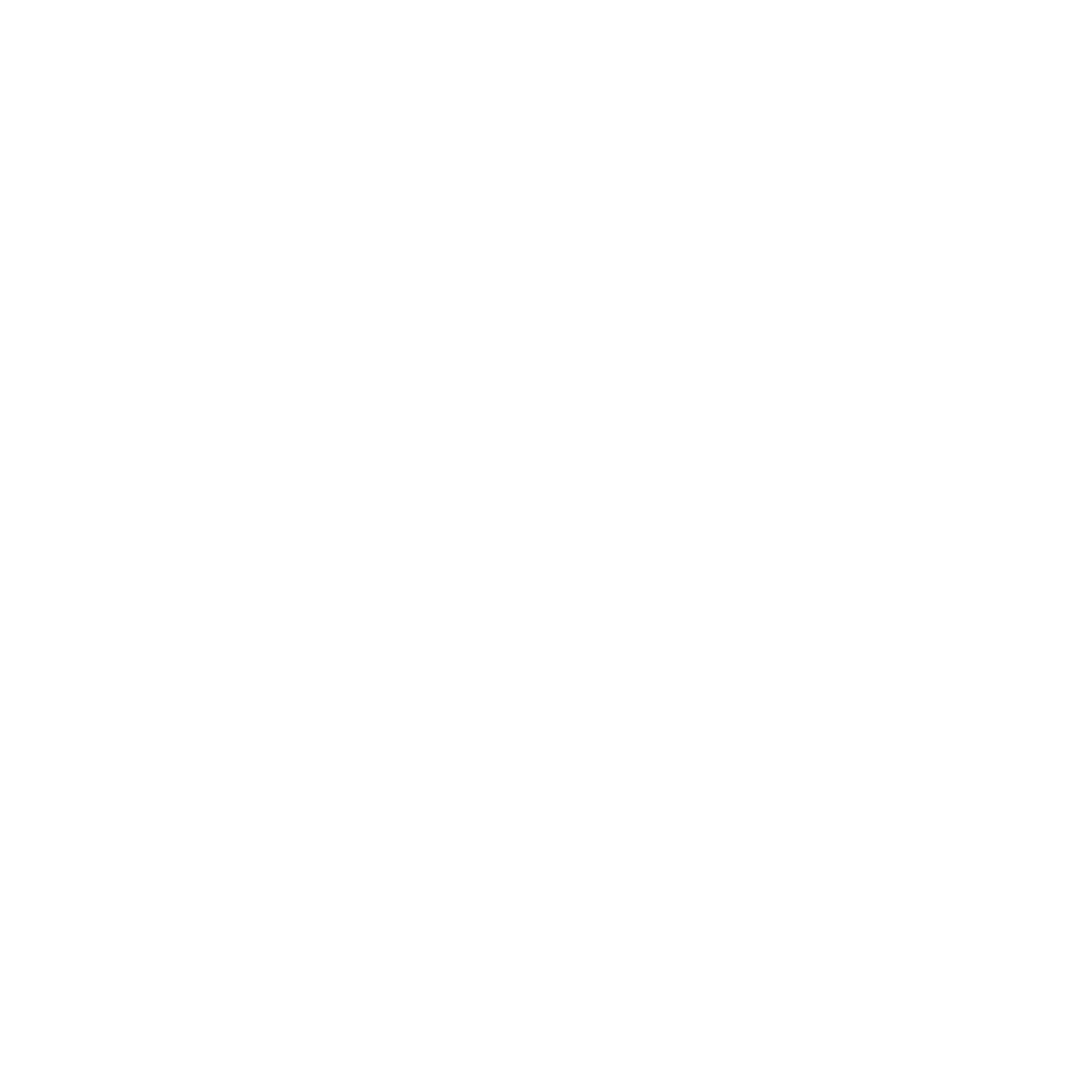

Supplement: Supplementary file 4 — Supplementary Information 4. [file 41598_2020_75814_MOESM4_ESM.zip › FluoSim/Resources/Fonts/font_glWord.png]

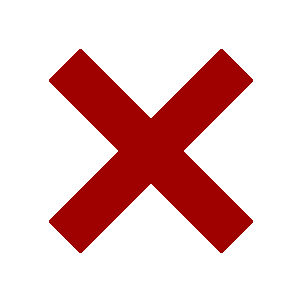

Supplement: Supplementary file 4 — Supplementary Information 4. [file 41598_2020_75814_MOESM4_ESM.zip › FluoSim/Resources/Icons/deleteButton.png]

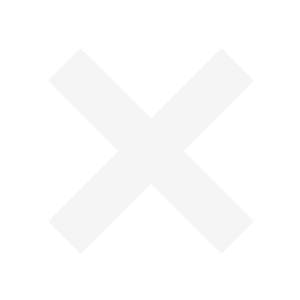

Supplement: Supplementary file 4 — Supplementary Information 4. [file 41598_2020_75814_MOESM4_ESM.zip › FluoSim/Resources/Icons/deleteButton_hover.png]

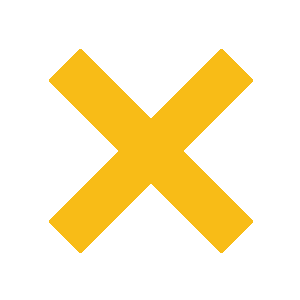

Supplement: Supplementary file 4 — Supplementary Information 4. [file 41598_2020_75814_MOESM4_ESM.zip › FluoSim/Resources/Icons/deleteButton_pressed.png]

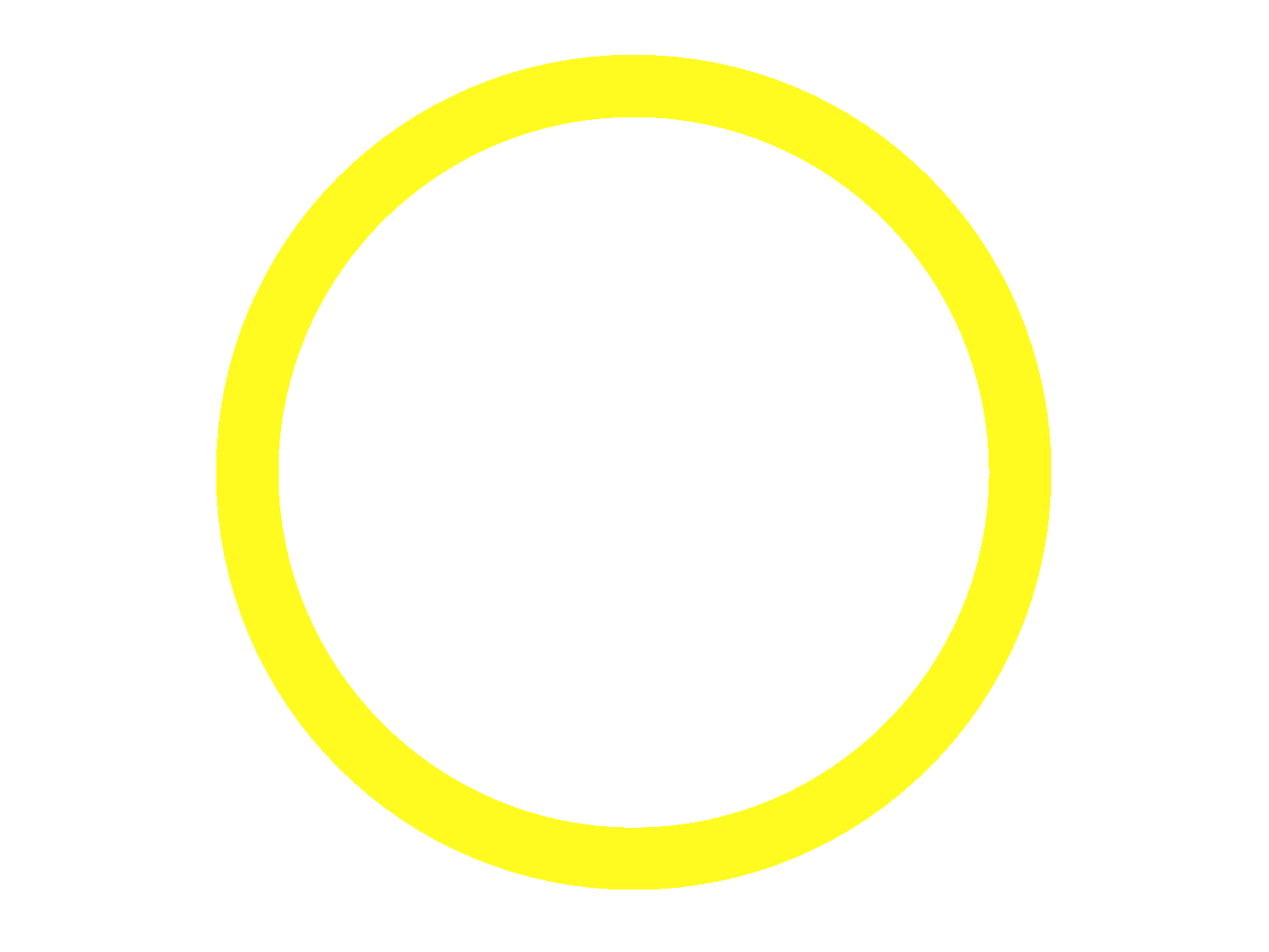

Supplement: Supplementary file 4 — Supplementary Information 4. [file 41598_2020_75814_MOESM4_ESM.zip › FluoSim/Resources/Icons/drawCircle-yellow_icon.png]

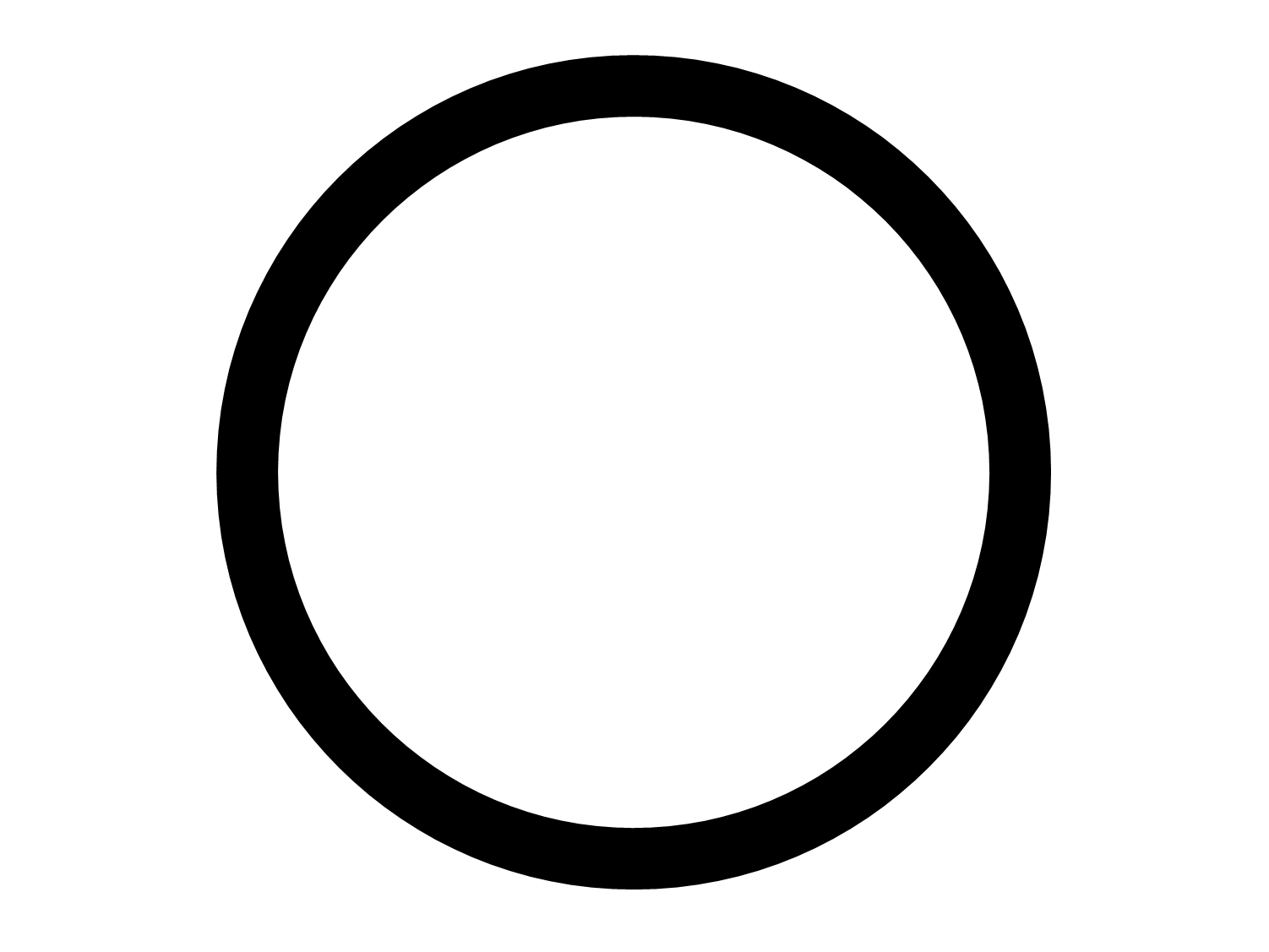

Supplement: Supplementary file 4 — Supplementary Information 4. [file 41598_2020_75814_MOESM4_ESM.zip › FluoSim/Resources/Icons/drawCircle_icon.png]

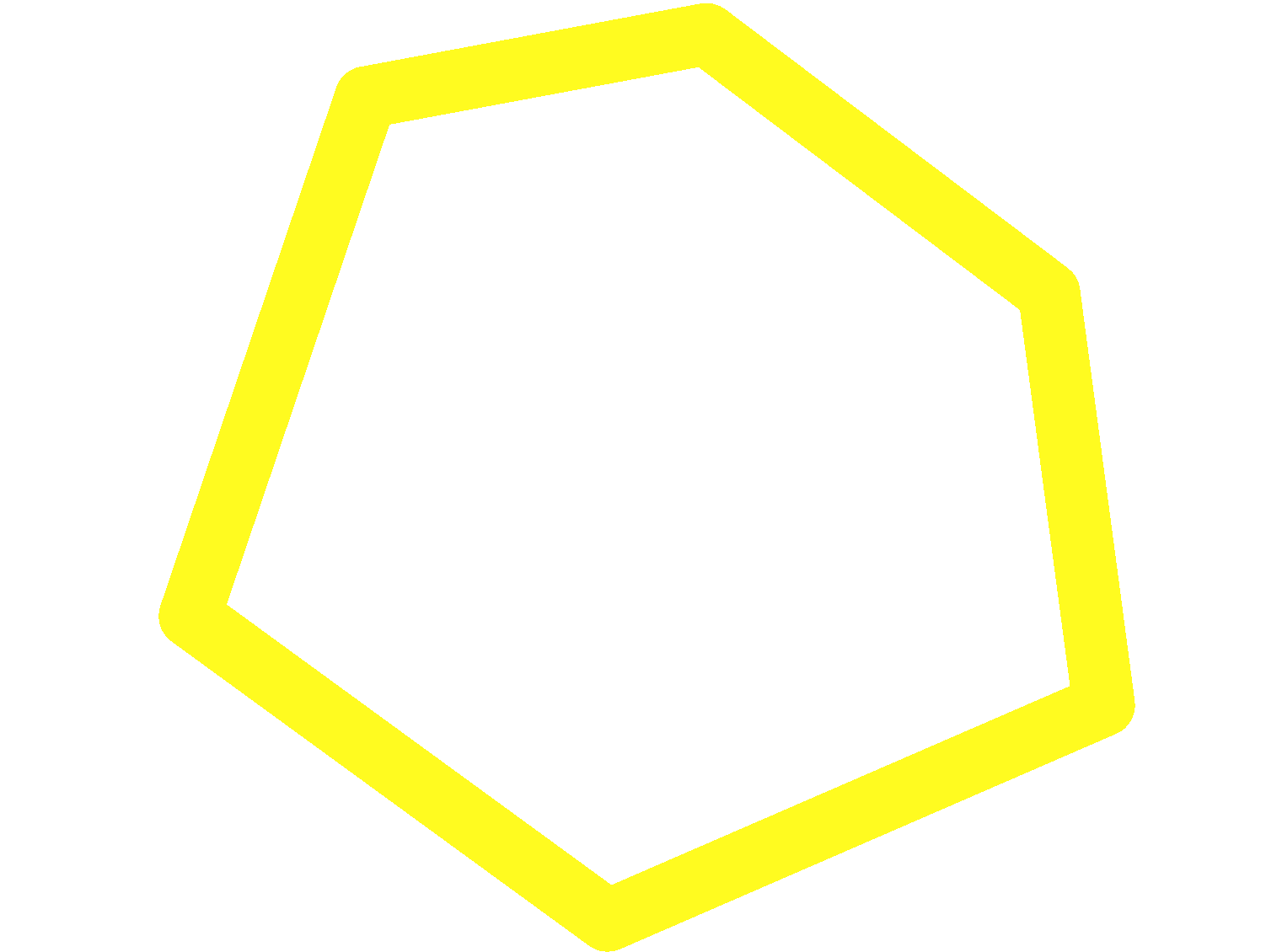

Supplement: Supplementary file 4 — Supplementary Information 4. [file 41598_2020_75814_MOESM4_ESM.zip › FluoSim/Resources/Icons/drawPolygon-yellow_icon.png]

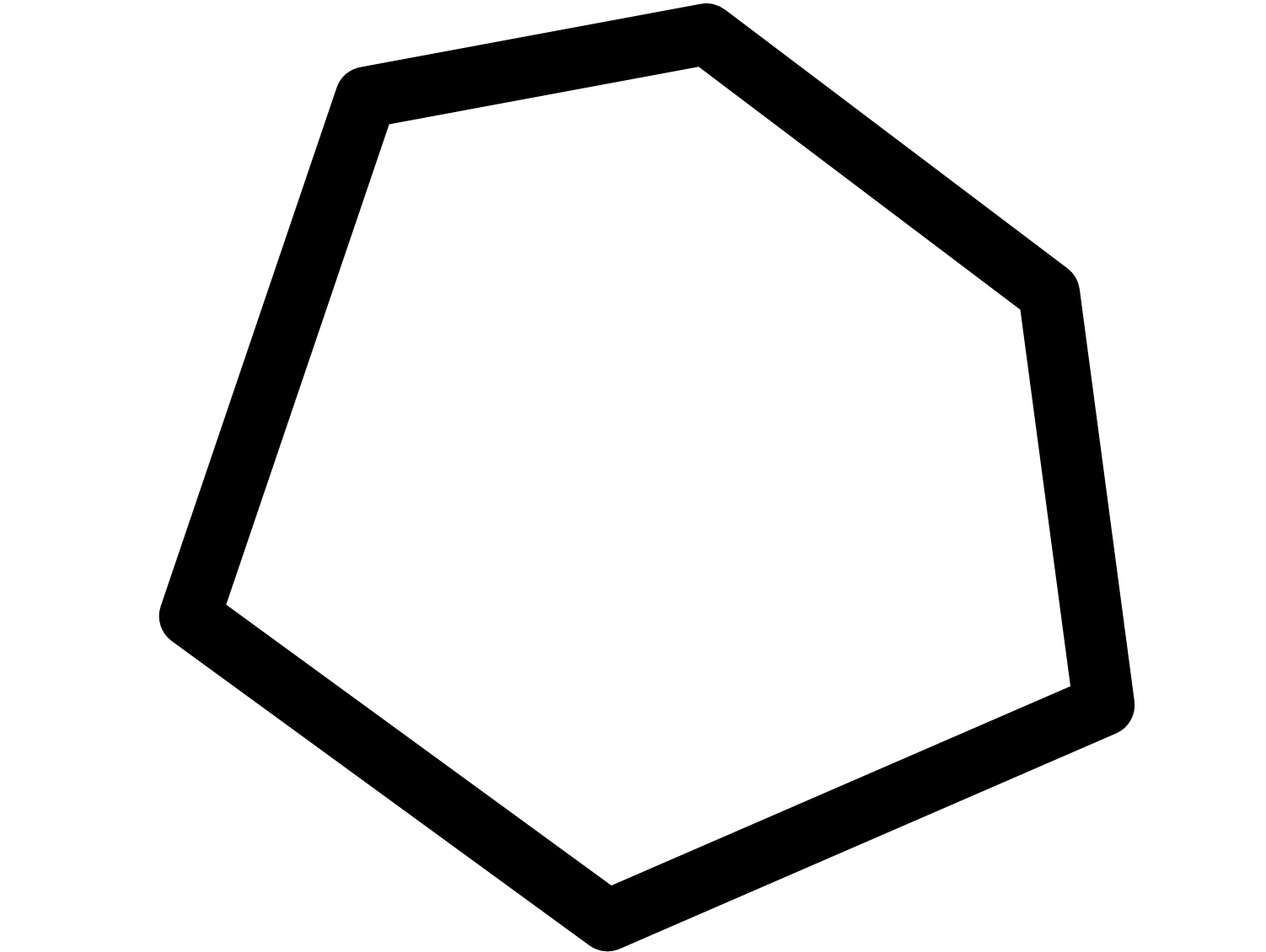

Supplement: Supplementary file 4 — Supplementary Information 4. [file 41598_2020_75814_MOESM4_ESM.zip › FluoSim/Resources/Icons/drawPolygon_icon.png]

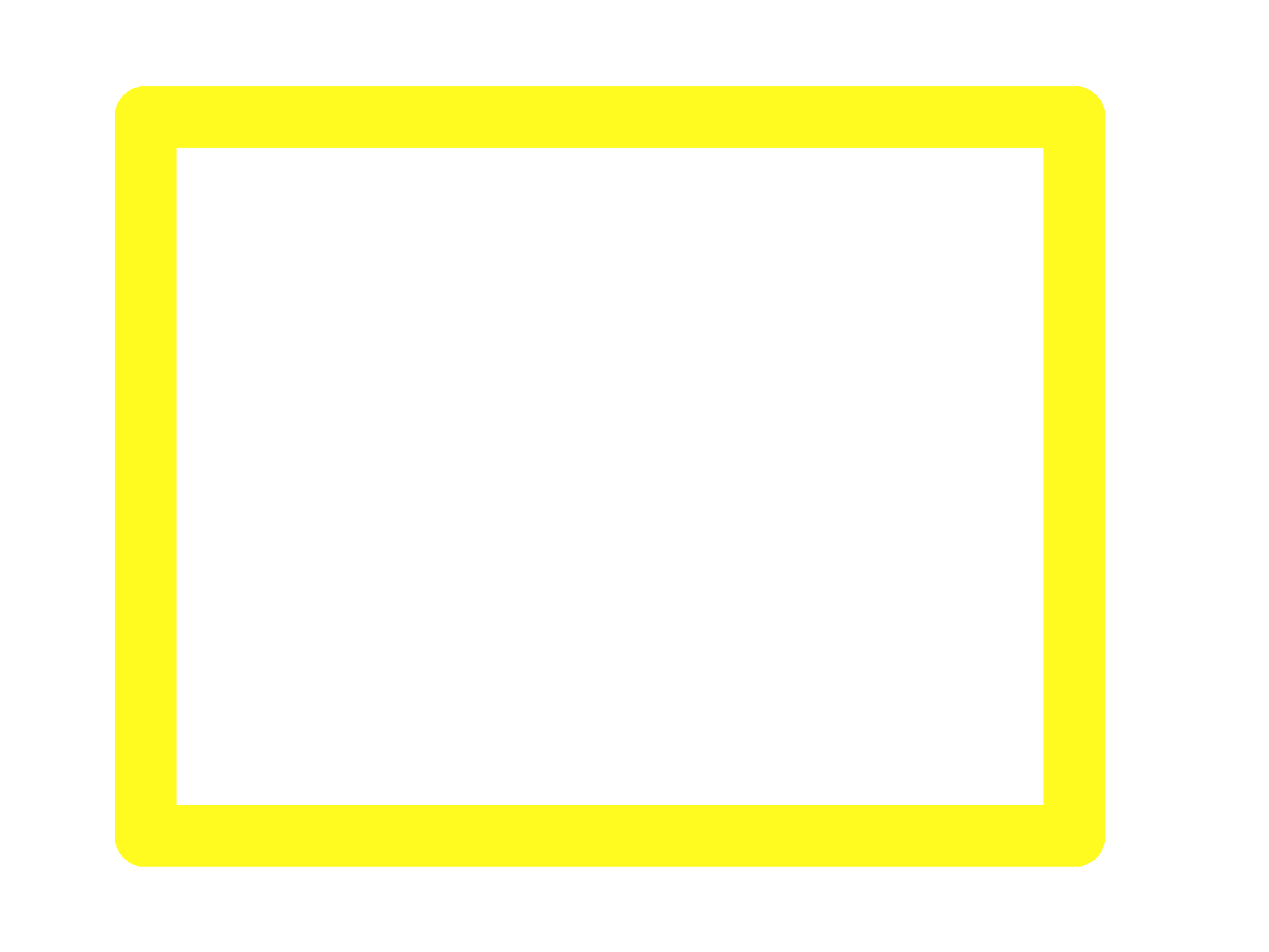

Supplement: Supplementary file 4 — Supplementary Information 4. [file 41598_2020_75814_MOESM4_ESM.zip › FluoSim/Resources/Icons/drawRect-yellow_icon.png]

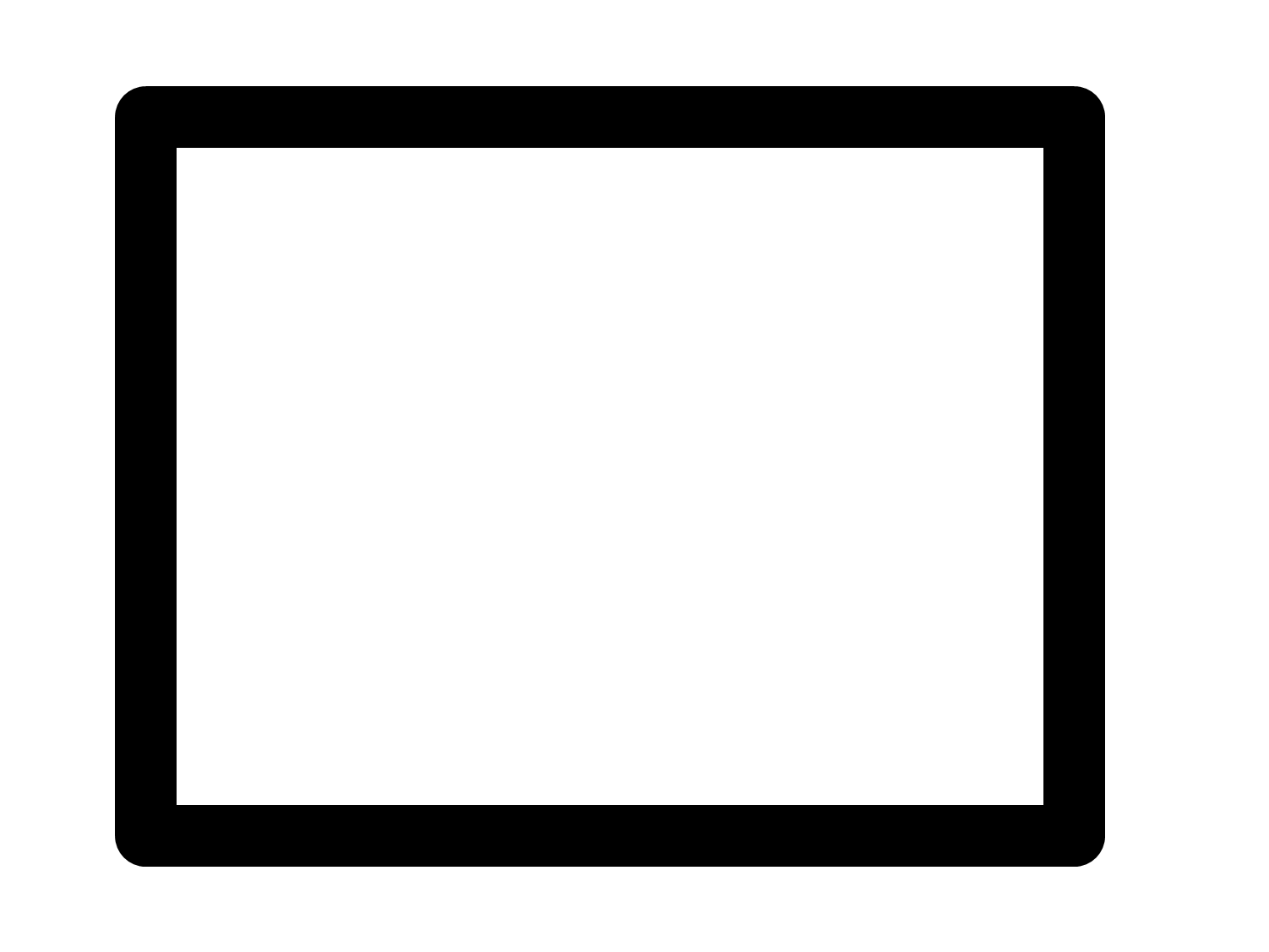

Supplement: Supplementary file 4 — Supplementary Information 4. [file 41598_2020_75814_MOESM4_ESM.zip › FluoSim/Resources/Icons/drawRect_icon.png]

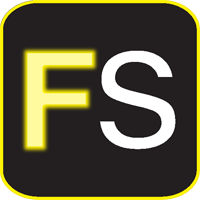

Supplement: Supplementary file 4 — Supplementary Information 4. [file 41598_2020_75814_MOESM4_ESM.zip › FluoSim/Resources/Icons/logo.png]

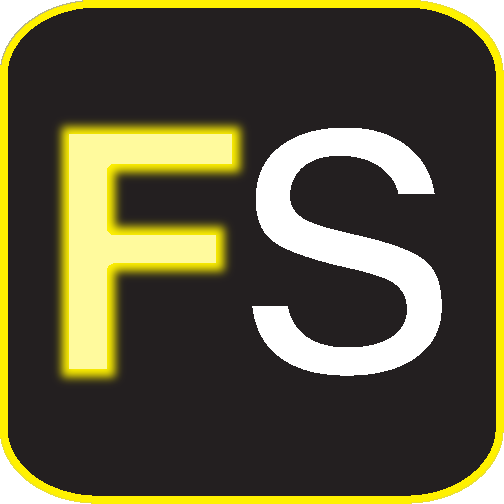

Supplement: Supplementary file 4 — Supplementary Information 4. [file 41598_2020_75814_MOESM4_ESM.zip › FluoSim/Resources/Icons/logo.tif]

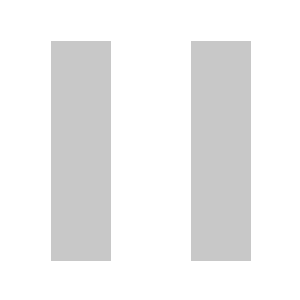

Supplement: Supplementary file 4 — Supplementary Information 4. [file 41598_2020_75814_MOESM4_ESM.zip › FluoSim/Resources/Icons/pauseButton.png]

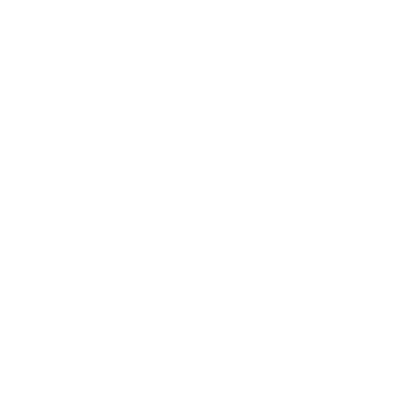

Supplement: Supplementary file 4 — Supplementary Information 4. [file 41598_2020_75814_MOESM4_ESM.zip › FluoSim/Resources/Icons/pauseButton_hover.png]

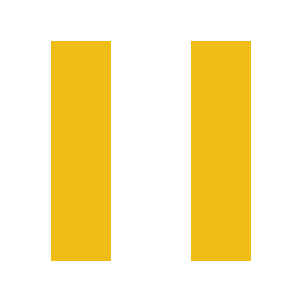

Supplement: Supplementary file 4 — Supplementary Information 4. [file 41598_2020_75814_MOESM4_ESM.zip › FluoSim/Resources/Icons/pauseButton_pressed.png]

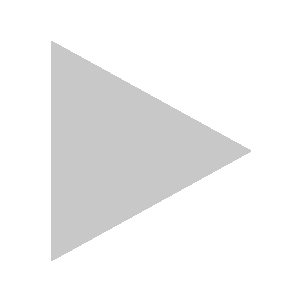

Supplement: Supplementary file 4 — Supplementary Information 4. [file 41598_2020_75814_MOESM4_ESM.zip › FluoSim/Resources/Icons/playButton.png]

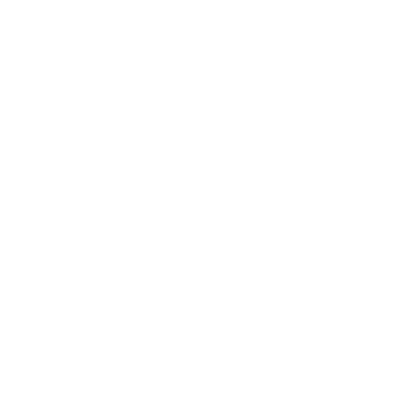

Supplement: Supplementary file 4 — Supplementary Information 4. [file 41598_2020_75814_MOESM4_ESM.zip › FluoSim/Resources/Icons/playButton_hover.png]

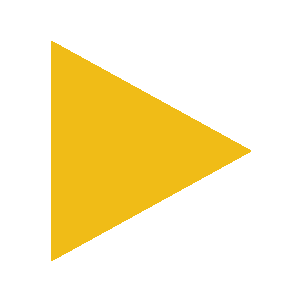

Supplement: Supplementary file 4 — Supplementary Information 4. [file 41598_2020_75814_MOESM4_ESM.zip › FluoSim/Resources/Icons/playButton_pressed.png]

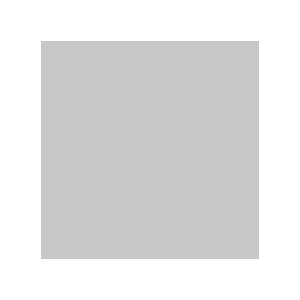

Supplement: Supplementary file 4 — Supplementary Information 4. [file 41598_2020_75814_MOESM4_ESM.zip › FluoSim/Resources/Icons/stopButton.png]

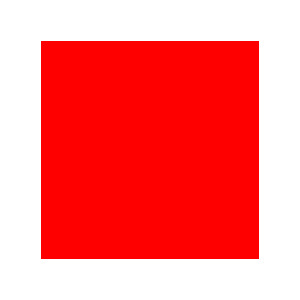

Supplement: Supplementary file 4 — Supplementary Information 4. [file 41598_2020_75814_MOESM4_ESM.zip › FluoSim/Resources/Icons/stopButton_disabled.png]

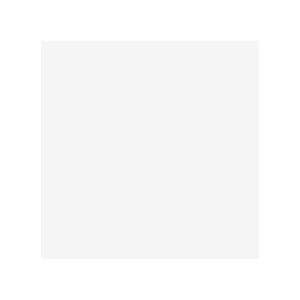

Supplement: Supplementary file 4 — Supplementary Information 4. [file 41598_2020_75814_MOESM4_ESM.zip › FluoSim/Resources/Icons/stopButton_hover.png]

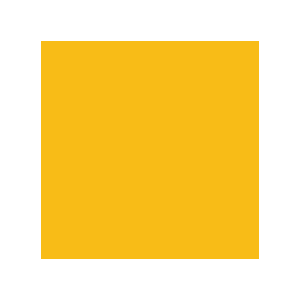

Supplement: Supplementary file 4 — Supplementary Information 4. [file 41598_2020_75814_MOESM4_ESM.zip › FluoSim/Resources/Icons/stopButton_pressed.png]

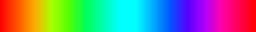

Supplement: Supplementary file 4 — Supplementary Information 4. [file 41598_2020_75814_MOESM4_ESM.zip › FluoSim/Resources/Luts/hsv.png]

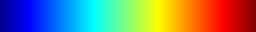

Supplement: Supplementary file 4 — Supplementary Information 4. [file 41598_2020_75814_MOESM4_ESM.zip › FluoSim/Resources/Luts/jet.png]

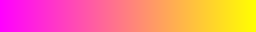

Supplement: Supplementary file 4 — Supplementary Information 4. [file 41598_2020_75814_MOESM4_ESM.zip › FluoSim/Resources/Luts/spring.png]

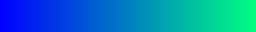

Supplement: Supplementary file 4 — Supplementary Information 4. [file 41598_2020_75814_MOESM4_ESM.zip › FluoSim/Resources/Luts/winter.png]
